# *Zanthoxylum-*specific whole genome duplication and recent activity of transposable elements in the highly repetitive paleotetraploid *Z. bungeanum* genome

**DOI:** 10.1038/s41438-021-00665-1

**Published:** 2021-09-03

**Authors:** Shijing Feng, Zhenshan Liu, Jian Cheng, Zihe Li, Lu Tian, Min Liu, Tuxi Yang, Yulin Liu, Yonghong Liu, He Dai, Zujun Yang, Qing Zhang, Gang Wang, Jisen Zhang, Huifeng Jiang, Anzhi Wei

**Affiliations:** 1 College of Forestry, Northwest A&F University, Yangling, Shaanxi China; 2 Research Centre for Engineering and Technology of Zanthoxylum State Forestry Administration, Yangling, Shaanxi China; 3 College of Life Science, Northwest A&F University, Yangling, Shaanxi China; 4 Key Laboratory of Systems Microbial Biotechnology, Tianjin Institute of Industrial Biotechnology, Chinese Academy of Sciences, Tianjin, China; 5 School of Ecology and Environment, Northwestern Polytechnical University, Xi’an, Shanxi China; 6 Biomarker Technologies Corporation, Beijing, China; 7 Center for Information in Biology, College of Life Science and Technology, University of Electronic Science and Technology of China, Chengdu, China; 8 Center for Genomics and Biotechnology, Haixia Institute of Science and Technology, Fujian Provincial Key Laboratory of Haixia Applied Plant Systems Biology, College of Life Sciences, Fujian Agriculture and Forestry University, Fuzhou, China

**Keywords:** Next-generation sequencing, Secondary metabolism

## Abstract

*Zanthoxylum bungeanum* is an important spice and medicinal plant that is unique for its accumulation of abundant secondary metabolites, which create a characteristic aroma and tingling sensation in the mouth. Owing to the high proportion of repetitive sequences, high heterozygosity, and increased chromosome number of *Z. bungeanum*, the assembly of its chromosomal pseudomolecules is extremely challenging. Here, we present a genome sequence for *Z. bungeanum*, with a dramatically expanded size of 4.23 Gb, assembled into 68 chromosomes. This genome is approximately tenfold larger than that of its close relative *Citrus sinensis*. After the divergence of *Zanthoxylum* and *Citrus*, the lineage-specific whole-genome duplication event η-WGD approximately 26.8 million years ago (MYA) and the recent transposable element (TE) burst ~6.41 MYA account for the substantial genome expansion in *Z. bungeanum*. The independent *Zanthoxylum-*specific WGD event was followed by numerous fusion/fission events that shaped the genomic architecture. Integrative genomic and transcriptomic analyses suggested that prominent species-specific gene family expansions and changes in gene expression have shaped the biosynthesis of sanshools, terpenoids, and anthocyanins, which contribute to the special flavor and appearance of *Z. bungeanum*. In summary, the reference genome provides a valuable model for studying the impact of WGDs with recent TE activity on gene gain and loss and genome reconstruction and provides resources to accelerate *Zanthoxylum* improvement.

## Introduction

As close relatives of *Citrus* in the Rutaceae family, plants of the genus *Zanthoxylum* generate strong tingling and numbing sensations in the mouth, which together with the pungent taste of hot chili form the spicy-hot flavor of Asian cuisine. This genus contains ~250 species native to tropical and subtropical regions worldwide, including Asia, America, and Africa^1^. Plants of this genus are well known for their ability to biosynthesize abundant important secondary metabolites, including flavonoids^2^, terpenoids^3^, and olefinic alkamides^4–6^. In particular, the tingling sensation caused by *Zanthoxylum bungeanum* is due to the accumulation of sanshools, a group of alkaloids that are unique to the genus *Zanthoxylum*^7,8^. Research findings have also indicated that secondary metabolites from *Zanthoxylum* species exhibited anticancer^9^, anesthetic^10^, analgesic^11^, antiwrinkle^12^, anti-inflammatory and other biological activities, suggesting great potential of these chemicals in the development of new drugs. Therefore, this genus has been widely used in the food industry^3,13^, cosmetics industry^12^, and traditional medicines^14–16^. The identification and utilization of critical medicinal and agrochemical compounds from *Zanthoxylum* plants have significant economic value and have thus attracted increasing research interest from plant biologists.

*Zanthoxylum bungeanum* (common name: *HuaJiao*), one of the earliest domesticated crops in this genus, has been cultivated for the last 2,000 years in southwest China^2^, which is thought to be the center of origin of *Zanthoxylum*. This region harbors 36 of the 41 Chinese *Zanthoxylum* species^17^. Ancient Chinese people regard the fruits of *Z. bungeanum* as a symbol of fertility, wealth, and longevity. Evidence for the medicinal use of *Z. bungeanum* can be traced back to the earliest traditional Chinese medicine monograph *ShenNongBenCaoJing* (The Divine Farmer’s Classic of Materia Medica), written during the Han Dynasty (206 BC–220 AD). Since then, *Z. bungeanum* has been included in prescriptions for the treatment of numerous diseases^18–20^. However, this plant was not used as a major spice until the Three Kingdoms period (3rd century AD). This timescale is much earlier than the introduction of hot chili to China. Currently, *Z. bungeanum* is still one of the major native spices widely consumed in China, with a cultivation area of 1.7 million hectares that accounts for an economic value of 4.0 billion USD (Supplemental Text). To date, multiple landraces and elite cultivars of *Z. bungeanum* have been developed through long-term conventional selective breeding efforts^2^.

Despite its importance as a native spice crop, *Z. bungeanum*-related genetic research is almost nonexistent. The availability of whole genome sequences for Rutaceae has been limited to *Citrus*^21–23^. This shortcoming impedes our understanding of the genome evolution and regulation of metabolic pathways for major characteristic constituents. Here, we present a reference genome of *Z. bungeanum*, employing a combination of three different sequencing technologies. The availability of the *Zanthoxylum* genome and transcriptome data not only highlights the unique evolutionary trajectory of the *Zanthoxylum* genome but also aids in deciphering the mechanisms of evolutionary regulation of metabolic pathways for alkamides, flavonoids, and terpenoids. Furthermore, the *Zanthoxylum* genome provides a good baseline for future comparative genomics in Rutaceae.

## Results

### Large genome assembly and annotation

**Fig. 1:**
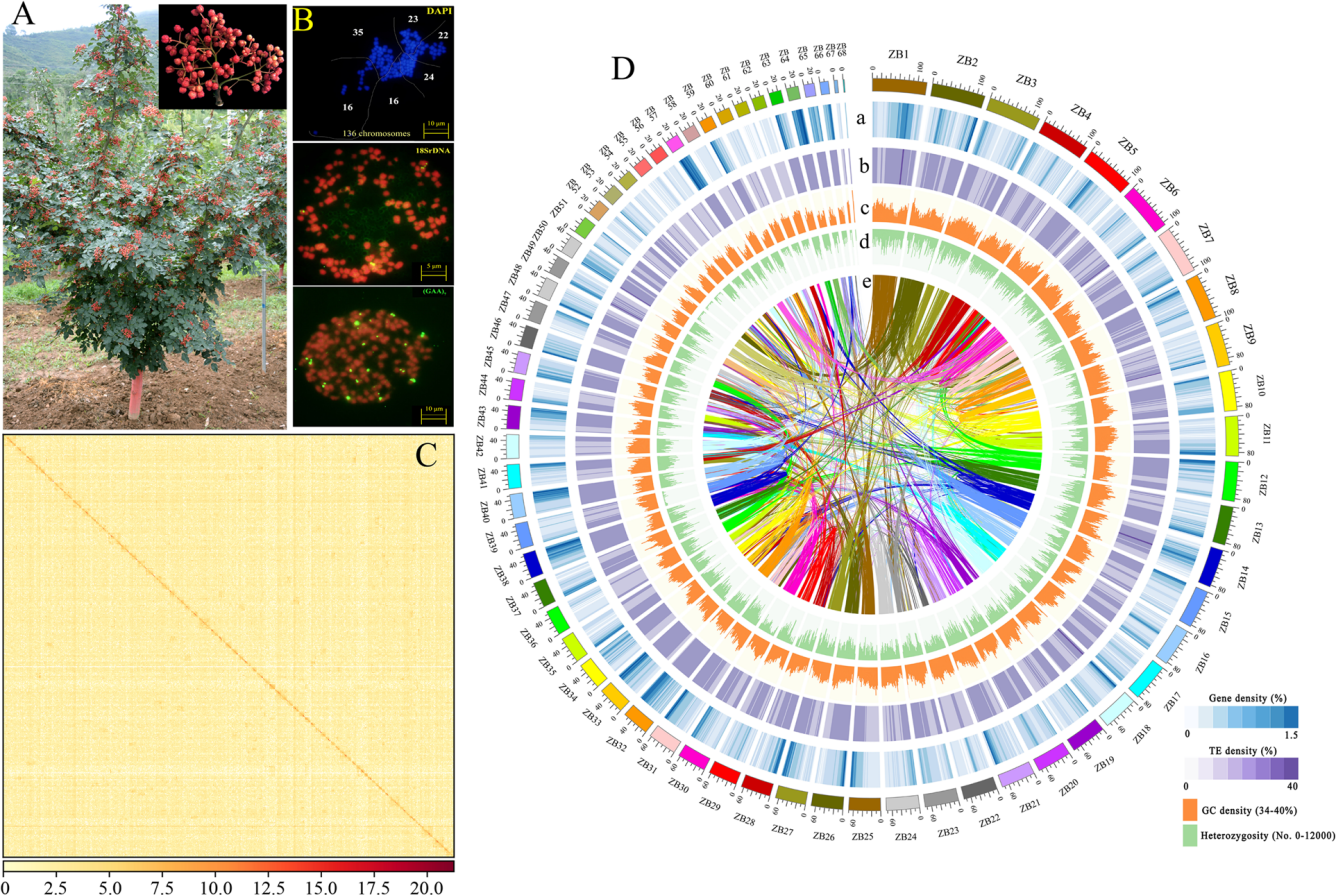
Morphological characteristics and genomic landscape of *Z. bungeanum*. **A** The whole tree and fruit features of *Z. bungeanum*. **B** Karyograms of the *Z. bungeanum* chromosomes based on FISH analysis with 18SrDNA and (GAA)_7_ probes (green signals). All of the chromosomes were stained with DAPI (blue). Bar is 5 μm. **C** Heatmap showing Hi-C interactions with a resolution of 500 kb. **D** Characteristics of 68 chromosomes of *Z. bungeanum*. Tracks a‒d show the distribution of gene density, transposable element (TE) density, GC density, and heterozygosity rate, with densities calculated in 2 Mb windows. Track e shows syntenic blocks

Due to its commercial and genetic importance, we selected the widely cultivated *Z. bungeanum* ‘DaHongPao’ (2*n* = 136) for genome sequencing (Fig. 1A, B; Figs. S1 and S2). We performed whole-genome sequencing analysis using the PacBio Sequel platform and Illumina HiSeq 2500 platform from seven paired-end libraries, which yielded 430 Gb long PacBio single-molecule real-time (SMRT) reads (Table S1) and 214 Gb Illumina reads (Table S2) for genome assembly. We preliminarily obtained a raw assembled genome of 5.25 Gb. After polishing by NextPolish^24^ and purging the haplotigs and error fragments by purge_dups, we obtained the final genome assembly with a length of 4.23 Gb and contig N50 of 410 kb, representing 95.5% of the estimated genome size by flow cytometry (Table 1, Fig. S3). However, this assembly was slightly larger than the estimated genome size by 21 kmer (4.11 Gb), which may be due to the high heterozygosity of *Z. bungeanum* (~2.87%, estimated by *k*-mer frequency, Fig. S4), as reported in pistachio^25^ and *Dendrobium officinale*^26^. We further scaffolded the *Z. bungeanum* genome to the chromosome scale using Hi-C scaffolding technologies. A total of 255.77 million valid Hi-C read pairs were mapped onto the draft assembly contigs using ALLHiC^27,28^. Finally, we obtained a genome with a total size of 4.12 Gb (98% of the primary assembly), containing 68 pseudochromosomes with a scaffold N50 of 74.18 Mb and the longest scaffold of 119.5 Mb (Fig. 1C, D, Table 1, Fig. S5, Table S3).

**Table 1:** Summary of the assembly and annotation of the *Z. bungeanum* genome.

Assembly	Statistics
Genome-sequencing depth (×)	100
Estimated genome size (Gb, by flow cytometry)	4.43
Assembly length (Gb)	4.23
Total number of contigs	16,879
Maximum contig length (Mb)	4.15
Minimum contig length (kb)	10.0
N50 contig length (kb)	410.07
Total number of scaffolds	332
N50 scaffold length (Mb)	74.18
Longest scaffold (Mb)	119.53
Assembly % of genome	98.44
GC content (%)	36.81
Heterozygosity rate (%)	2.87
**Annotation**	**Number**
No. of genes	74,307
Average coding sequence length (kb)	3725
Percentage of gene length in the genome (%)	6.53
Repeat region ratio of assembly (%)	89.14
No. of exons	397,906
Average exon sequence length (bp)	237.62
No. of introns	397,905
Average intron sequence length (bp)	392.83
miRNA	422
rRNA	454
tRNA	1406

Putative protein-coding and microRNA genes were annotated based on a comprehensive strategy combining ab initio prediction, homology gene modeling, and transcriptional evidence obtained in this study (Fig. S6, Table S4). A total of 74,307 protein-coding genes were predicted from this assembly (Tables 1), 99.09% (73,633) of which were supported by the presence of homology to known proteins, the existence of known functional domains, or the presence of expressed transcripts (Table S5). Additionally, 2,282 noncoding RNA sequences were identified and annotated, including 422 microRNAs (miRNAs), 454 ribosomal RNAs (rRNAs), and 1,406 transfer RNAs (tRNAs) (Table 1). To assess the genome quality and annotation completeness, we checked the core gene statistics using Benchmarking Universal Single-Copy Orthologs (BUSCO) and Conserved Core Eukaryotic Gene Mapping Approach (CEGMA), which suggested that 97.59% (2,270 of 2,326) and 97.82% (448 of 458) of the genes were recovered, respectively (Tables S6 and S7). In addition, our assembled genome obtained a relatively high long terminal repeat (LTR) assembly index (LAI) score (15.36). Taken together, these comparisons indicated that our genome assembly attained reference-level quality.

### The genome evolution of *Z. bungeanum*

**Fig. 2:**
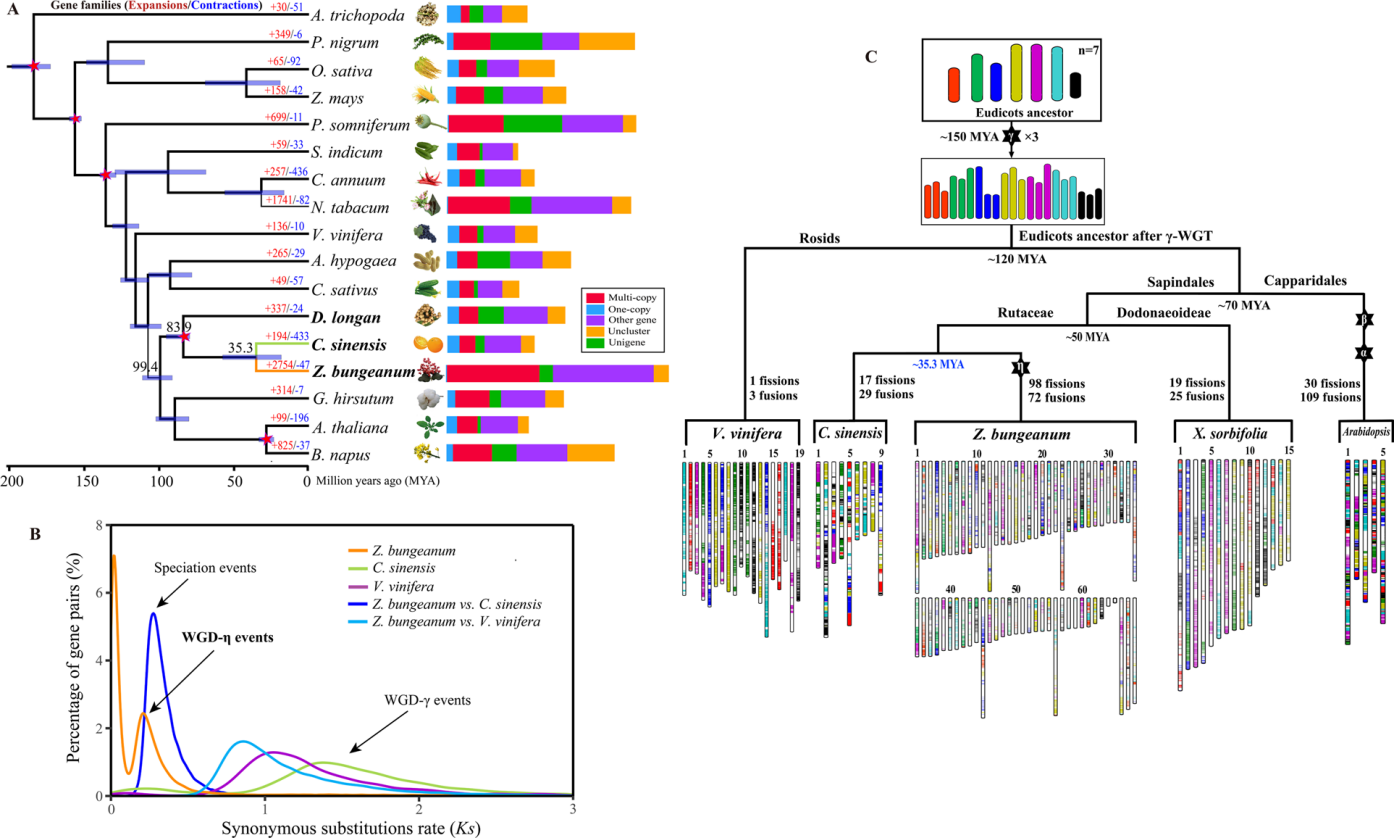
The evolutionary history of *Z. bungeanum*. **A** Phylogenetic tree based on 659 single-copy genes from 17 species: *Z. bungeanum*, *A. trichopoda*, *P. nigrum*, *Z. mays*, *O. sativa*, *P. somniferum*, *V. vinifera*, *D. longan*, *A. thaliana*, *B. napus*, *G. hirsutum*, *A. hypogaea*, *C. sativus*, *S. indicum*, *C. annuum*, and *N. tabacum*. Numbers over the branches indicate the number of expansions (red) and contractions (blue) of gene families in different plants. Light blue bars at the internodes indicate divergence times with 95% confidence intervals (CIs). The red pentagrams on the nodes represent five calibration points. The histogram shows the clusters of orthologous and paralogous gene families in the 17 species identified by OrthoMCL. One copy: only one gene coming from one species within a family (the family includes 17 species); Multicopy: at least two genes coming from one species within a family; Unigene: genes within a family only coming from one species (the family includes one species); Other gene: one or more genes coming from one species within a family (the family includes less than 17 species); UnCluster: genes not involved in clustering. **B** Distribution of synonymous substitution rates (*Ks*) for paralogs in *Z. bungeanum*. Gene duplication analysis in comparison to grape indicated that one recent WGD event occurred in the *Z. bungeanum* genome. **C** Evolutionary scenario of chromosome numbers in *Z. bungeanum* from 21 (post-γ-WGT) chromosomes and 7 (pre-γ-WGT) protochromosomes. The modern genomes of *V. vinifera*, *C. sinensis*, *Z. bungeanum*, *X. sorbifolia*, and *A. thaliana* are presented at the bottom with different colors to illustrate the evolution of segments from the common ancestor with 7 chromosomes (top). Polyploidization events are indicated by stars (γ-WGT; α-, β-, ρ-WGD), along with shuffling events (fusions and fissions)

The evolution of gene families was analyzed by comparing the *Z. bungeanum* genome with that of 16 other plant species, including *Amborella trichopoda*, *Piper nigrum*, *Zea mays*, *Oryza sativa*, *Papaver somniferum*, *Vitis vinifera*, *Dimocarpus longan*, *Arabidopsis thaliana*, *Brassica napus*, *Gossypium hirsutum*, *Arachis hypogaea*, *Cucumis sativus*, *Sesamum indicum*, *Capsicum annuum*, *Citrus sinensis*, and *Nicotiana tabacum*. In total, 577,729 genes were clustered into 52,558 orthologous gene families, of which 5,664 gene families were shared by all 17 species, representing the ancestral gene families, and 532 gene families were specific to Rutaceae plants (Fig. S7, for clarity, only *Z. bungeanum*, *C. annuum*, *P. nigrum*, *D. longan*, and *C. sinensis* are shown). We found a total of 1,693 *Z. bungeanum*-specific gene families consisting of 4,498 genes (Table S8), which were enriched in genes associated with C5-branched dibasic acid metabolism, terpenoid backbone biosynthesis, unsaturated fatty acid biosynthesis, and valine, leucine, and isoleucine biosynthesis, among others (Table S9). We also identified a total of 2,754 gene families that were significantly (*P* < 0.05) expanded in *Z. bungeanum* and 47 gene families that were significantly contracted since the split from the common ancestor with *C. sinensis*. However, *C. sinensis* showed fewer gene family expansions and more gene family contractions than other species in the order Sapindales (Fig. 2A). Based on the Kyoto Encyclopedia of Genes and Genomes (KEGG) annotations, expanded gene families were highly enriched in various secondary metabolites, including sesquiterpenoid and triterpenoid biosynthesis, flavonoid biosynthesis, phenylpropanoid biosynthesis, linoleic acid metabolism, phenylalanine metabolism, and anthocyanin biosynthesis (Table S10).

To investigate the evolution of *Zanthoxylum*, we derived 659 single-copy genes from the 17 species for phylogenetic analysis (Table S11). The resulting phylogeny indicated that *Z. bungeanum* was most closely related to *C. sinensis*, as expected, and that these two species formed the Sapindales clade along with *D. longan*. Molecular dating, derived using five calibration points, suggested that *Z. bungeanum* diverged from the most recent common ancestor of *C. sinensis* approximately 35.3 million years ago (MYA; 95% confidence interval [CI]: 18.47–57.67 MYA) (Fig. 2A). The families Rutaceae and Sapindaceae (*D. longan*) shared a common ancestor approximately 83.9 MYA (Fig. 2A).

There were significantly more multicopied gene families in *Z. bungeanum* than in other rosids (Fig. 2A, stack bar and Table S8), which is suggestive of at least one recent whole-genome duplication (WGD) event in the *Zanthoxylum* lineage. The distributions of synonymous substitutions per synonymous site (*K*_*S*_) of paralogous genes in the *Z. bungeanum* genome showed a single peak at approximately 0.21, but no similar peak was identified in *C. sinensis* (Fig. 2B), suggesting the occurrence of a recent WGD event experienced by *Zanthoxylum* (η-WGD) that was not shared among other Rutaceae members. These results combined with the phylogenetic analysis (Fig. 2A) indicated that the η-WGD of *Z. bungeanum* occurred after the divergence of *Citrus* and *Zanthoxylum*. To investigate WGD in the *Z. bungeanum* genome, we performed a comparative genomic analysis of *Z. bungeanum* with *C. sinensis* and *V. vinifera*. We identified a 2:1 syntenic depth ratio in both *Z. bungeanum*-*C. sinensis* and *Z. bungeanum*-*V. vinifera* comparisons, and these syntenic blocks contained 6,258 and 5,578 pairs of gene models in the *Z. bungeanum* genome, respectively (Fig. S8). Genomic collinearity of *Z. bungeanum* with itself identified 2.50 G intragenomic blocks, including 50,631 gene pairs derived from the η-WGD event. Therefore, we concluded that a single *Zanthoxylum* lineage-specific η-WGD event occurred after the divergence between *Zanthoxylum* and *Citrus*. According to the divergence rate between *Z. bungeanum* and *C. sinensis*, the η-WGD event occurred approximately 26.8 MYA (Fig. 2A, B), which is much later than the ancient γ-WGD event (~120 MYA) that occurred in the ancestors of core eudicots. Additionally, we performed KEGG enrichment on the duplicated genes generated by η-WGD and found that most of them are involved in the proteasome, mRNA surveillance pathway, carbon fixation in photosynthetic organisms, plant hormone signal transduction, and some secondary metabolites, such as fatty acid metabolism, unsaturated fatty acid biosynthesis, pyruvate metabolism, and terpenoid backbone biosynthesis (Table S12).

The high number of chromosomes is an important feature of the *Z. bungeanum* genome. To assess the chromosome evolution of *Zanthoxylum*, we placed the 68 extant chromosomes into major groups, corresponding to regions most clearly identifiable as originating from one of the seven chromosomes that existed before the core eudicot triplication (γ-WGT, Fig. 2C). The 19 grape chromosomes were postulated to be the closest modern representative of the ancestral eudicot karyotype^29^. The genome of *A. thaliana* supported two recent whole-genome duplication events (α-WGD and β-WGD) and one triplication event (γ-WGT) that gave rise to much of the eudicot clade^30^. At least 109 fission/fusion events occurred in the five chromosomes of *A. thaliana* that evolved from the proposed paleohexaploid ancestor. A minimum of 17 chromosomal fissions and 29 chromosomal fusions were necessary for *C. sinensis* to reach its current structure of nine chromosomes, and 19 fissions and 25 fusions were necessary for *Xanthoceras sorbifolia* to reach 15 modern chromosomes. However, *Z. bungeanum* experienced a much more complex evolutionary history with a lineage-specific WGD (η-WGD, Fig. 2B), in addition to the shared ancestral γ-WGT. We speculated that *Z. bungeanum* might have experienced at least 98 chromosomal fissions and a minimum of 72 chromosomal fusions to reach its present karyotype of 68 chromosomes (Fig. 2C), indicating a high level of genome reconstruction in *Z. bungeanum*.

### Repetitive sequence expansions led to the large genome size in *Z. bungeanum*

**Fig. 3:**
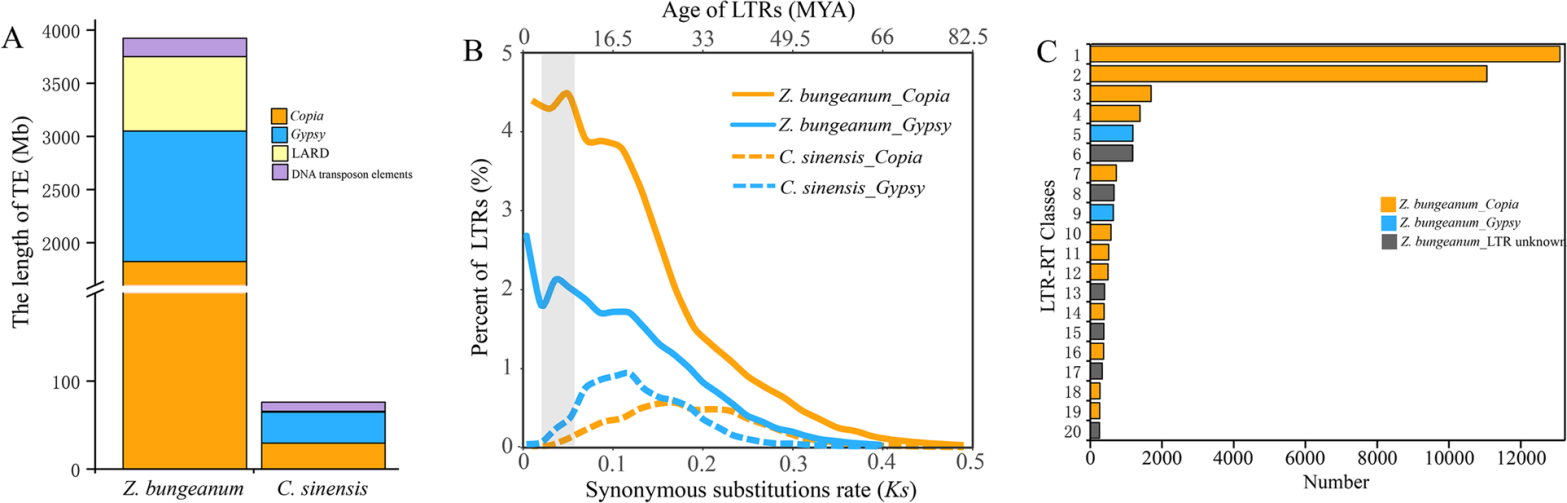
Comparisons of transposable element (TE) compositions in the *Z. bungeanum* and *C. sinensis* genomes. **A** The proportions of TEs in *Z. bungeanum* genome. **B** Age distribution of *Copia* and *Gypsy* insertions in *Z. bungeanum* and *C. sinensis*. A substitution rate of 3.92 × 10^−9^ per site per year was used to calculate the insertion times. The vertical gray bar indicated the time of TE bursts (~6.41 MYA). **C** Copy numbers for different long terminal repeat (LTR) families. The top twenty LTRs are presented, which indicate that two types of *Copia* elements are predominant

The assembled genome size of *Z. bungeanum* (4.23 Gb) is approximately tenfold larger than that of its close relative *C. sinensis* (~0.38 Gb), despite sharing considerably conserved syntenic blocks (Fig. S8, Table S13). In fact, the size of the *Z. bungeanum* genome is the third largest among sequenced dicots thus far and is only smaller than that of tobacco^31^ and chickpea^32^ (Fig. S9). We identified and masked 3.78 Gb of the assembly as repetitive elements, which constituted ~89% of the *Z. bungeanum* genome. Among these elements, LTR retrotransposons were the most abundant transposable elements (TEs), of which *Copia* elements (43.04%) were a relatively larger component of the repeat landscape than *Gypsy* elements (29%) (Fig. 3A, Table S14).

Similar to other plants, the majority (97.4%) of TEs were located in intergenic regions rather than in exons and introns (Fig. S10). To trace the evolutionary dynamics of TEs, we investigated the insertion dates of *Copia* and *Gypsy* elements in *Z. bungeanum* and *C. sinensis*. A peak of increased insertion activity for both *Copia* and *Gypsy* appeared at ~6.41 MYA (Fig. 3B). Specifically, two types of *Copia* elements were dominant and contributed the most to *Z. bungeanum* genome expansion (Fig. 3C, Fig. S11). Compared with *C. sinensis*, a number of diverse and young LTR subfamilies were present in the *Z. bungeanum* genome (Fig. 3B), along with numerous species-specific LTRs (Fig. S11B, C). Of the identified TEs, only 19.59% were inherited from ancestral repeats, whereas 71.25% of the lineage-specific TEs emerged during genome expansion (Fig. S12).

### Genomic basis of the fruit quality of *Z. bungeanum*

The quality of *Z. bungeanum* fruit is determined by the numbing and tingling taste, fragrance, and appearance, corresponding to three major characteristic constituents: alkamides, terpenes, and anthocyanidins. Here, we investigated potential molecular mechanisms associated with *Z. bungeanum* fruit traits through a comprehensive comparative transcriptome analysis at different fruit development stages.

### Insights into sanshool biosynthesis

**Fig. 4:**
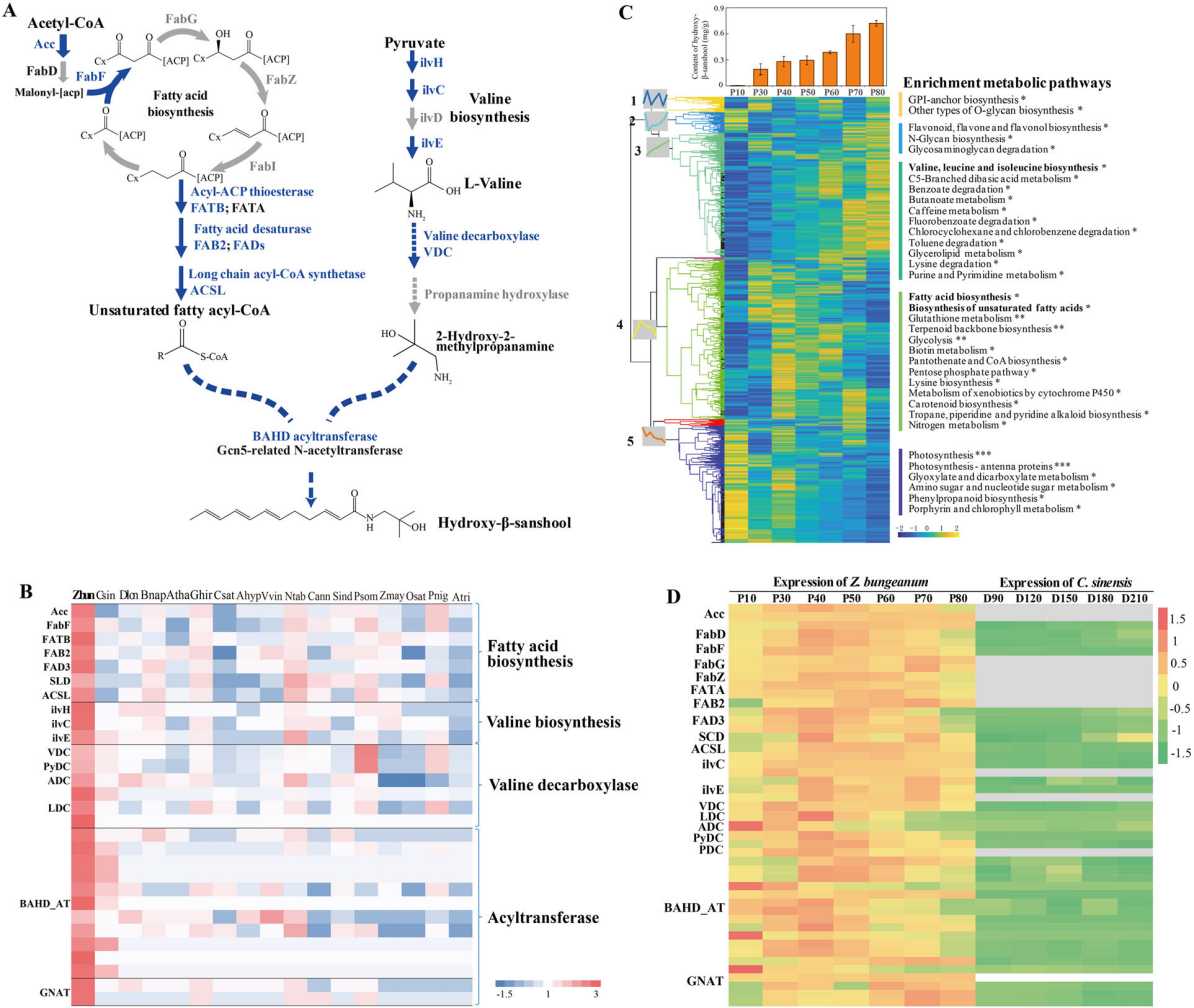
The metabolic pathways and protein families for sanshool biosynthesis. **A** Schematic representation of sanshool biosynthesis pathways. The solid lines indicate genes catalyzing major reactions that were characterized. The dotted lines indicate unclear pathways. The blue color indicates the species-specific expanded genes in *Z. bungeanum*. **B** Expansion of gene families involved in sanshool biosynthesis pathways. **C** Global heatmap (left heatmap) showing transcripts in the pericarp at seven developmental stages corresponding to 2752 metabolic genes. The genes were clustered by MATLAB based on the Spearman correlation of the expression profile. The right panel represents the KEGG metabolic pathway enrichment, and the asterisks *, **, and *** represent the *p* values ≤ 1e-2, 1e-6, and 1e-10, respectively. The top histogram represents the content of hydroxy-β-sanshool (mg/g) during different fruit development periods (10, 30, 40, 50, 60, 70, and 80 days postanthesis). **D** Comparison of the expression level of genes involved in sanshool biosynthesis pathways between *Z. bungeanum* and *Citrus sinensis*. Only the orthologs with FPKM > 5 in *Z. bungeanum* or *C. sinensis* are shown in the heatmaps. Gray color represents a gene present in *Z. bungeanum* but not in *C. sinensis*. The transcriptome of *C. sinensis* was referenced from a previous study^96^, and comparison of the transcriptomes between the two species is described in the Methods section in more detail.

Sanshools are synthesized from two direct precursor substrates, an unsaturated fatty acid moiety and propanamine^4,33^, in a reaction catalyzed by a potential acetyltransferase (NAF) (Fig. 4A). We identified 24 sanshool-like compounds from the pericarp of *Z. bungeanum*, 13 of which were recently discovered (Fig. S13, Table S15)^34,35^, and we found that the fatty acid moiety is often a 12 C or 14 C unsaturated fatty acyl-CoA (Fig. S13), which is biosynthesized by acyl-ACP thioesterase and fatty acid desaturase (Fig. 4A). The amines are biosynthesized in two steps: a valine decarboxylation reaction through branched chain amino acid (BCAA) decarboxylase and a hydroxylation reaction to produce 2-hydroxy-2-methylpropanamine through a cytochrome P450 hydroxylase (Fig. 4A).

Gene family expansion may be involved in the biosynthesis of a large number of different sanshools. We found seven expansive gene families involved in the biosynthesis of unsaturated fatty acyl-CoA, which included acetyl-CoA carboxylase carboxyl transferase subunit alpha (AccA, 16 genes), 3-oxoacyl-[acyl-carrier-protein] synthase II (FabF, 21 genes), fatty acyl-ACP thioesterase B (FATB, 22 genes), soluble fatty acid desaturase (FAB2, 14 genes), two classes of membrane-bound fatty acid desaturases (FADs, 24 genes), and long chain acyl-CoA synthetase (ACSL, 28 genes) (Fig. 4B). The abundant acyl-ACP thioesterase B in *Z. bungeanum*, which is approximately 13-fold higher than that of *C. sinensis*, could provide a variety of fatty acid precursors for sanshool biosynthesis (Fig. S14, Table S16).

Regarding the biosynthesis of propanamine, three of the four gene families involved in valine biosynthesis were significantly expanded; however, the mechanism of action of valine decarboxylase and propanamine hydroxylase in *Z. bungeanum* is still unclear. We analyzed all possible amino acid decarboxylases and found that 6 out of 17 gene families identified in *Z. bungeanum* were significantly expanded. Among them, a gene family annotated as group II pyridoxal-dependent decarboxylase is the ortholog of the verified VDC in *Echinacea purpurea*^33^. We analyzed two kinds of *N*-acetyltransferases, BAHD acyltransferase and Gcn5-related *N*-acetyltransferase, and found that there were 11 gene families of BAHD acyltransferases (158 genes) that were significantly expanded in *Z. bungeanum* compared to only two expanded gene families of Gcn5-related *N*-acetyltransferase (12 genes). This result implied that the potential *N*-acetyltransferase for sanshool biosynthesis may be a BAHD acyltransferase (Fig. S15), similar to capsaicin synthase in *Capsicum annuum*^36^.

The abundance of sanshools gradually increased in the pericarp during postanthesis^37^. As expected, the level of the typical hydroxy-β-sanshool also increased with fruit development (Fig. 4C, top histogram). To examine the correlation between the gene expression and abundance of sanshools, we constructed a coexpression network using RNA-Seq data from seven fruit development stages. Gene expression profiles for 2,752 metabolic genes were clustered into five modules (Fig. 4C, left heatmap, Fig. S16, Table S17). Furthermore, KEGG metabolic pathway enrichment analysis was performed for each module (Fig. 4C, right panel, Table S18). Both the fatty acid pathway and branched chain amino acid pathway were observed to be involved in the biosynthesis of sanshools. We observed that there was an enrichment of saturated and unsaturated fatty acid biosynthesis in module 4, with an increase in gene expression in the early stages but a reduction in the later stages. These results demonstrated that fatty acids were biosynthesized mainly during the intermediate stage of pericarp development. We also found that valine, leucine, and isoleucine biosynthesis was significantly enriched in module 3, in which gene expression increased throughout pericarp development (Fig. 4C). The reinforced biosynthesis of branched-chain amino acids can afford amine precursors for the synthesis of sanshools.

We further examined the gene expression profile involved in sanshool biosynthesis and their orthologous genes in *Citrus*, which does not produce a tingling sensation. We found 23,603 orthologous pairs between *Z. bungeanum* and *C. sinensis*, of which 2,874 pairs showed significantly higher expression levels in *Z. bungeanum* pericarps than in *C. sinensis*. Among these, 38 of 193 pairs related to the sanshool biosynthesis pathway showed significantly higher expression levels in the pericarp of *Z. bungeanum* (Fig. 4D, Fig. S17), and the proportion was significantly higher than that in the background (*P* = 0.002). The enrichment of highly expressed genes involved in sanshool biosynthesis not only indicates the underlying genetic basis for the accumulation of sanshools in *Z. bungeanum* but also provides a potential gene set for the identification of undetermined steps in its biosynthesis pathway.

### Characteristics of anthocyanidin synthase (ANS) in *Z. bungeanum*

**Fig. 5:**
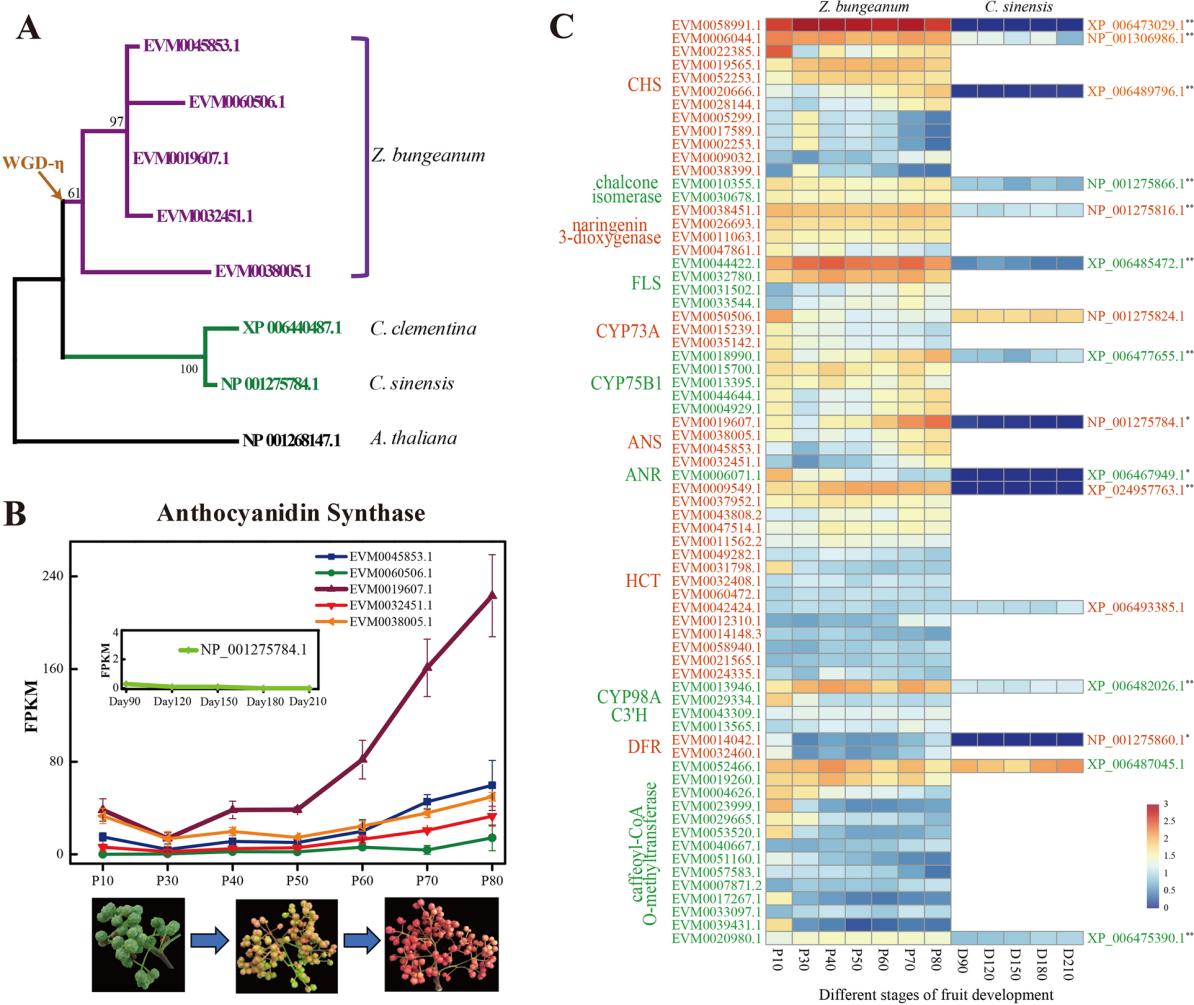
Evolutionary analysis and differentially expressed genes involved in the anthocyanidin synthase (ANS) pathway at seven fruit developmental stages in *Z. bungeanum*. **A** Gene expansion of the ANS genes in *Z. bungeanum* (purple) compared to *Citrus sinensis* (green) and *A. thaliana* (black). **B** Expression levels of five ANS genes at seven fruit developmental periods. The gene expression of NP_001275784.1 in *C. sinensis* was referenced from a previous study^96^. **C** Comparative analysis of gene expression involved in flavonoid biosynthesis between *Z. bungeanum* and *C. sinensis*. CHS: chalcone synthase, FLS: flavonol synthase, CYP73A: trans-cinnamate 4-monooxygenase, CYP75B1: flavonoid 3’-monooxygenase, ANS: anthocyanidin synthase, ANR: anthocyanidin reductase, HCT: shikimate O-hydroxycinnamoyltransferase, CYP98A, C3’H: coumaroylquinate (coumaroylshikimate) 3’-monooxygenase. * and ** represent *p* values ≤ 0.05 and 0.01, respectively

The *Z. bungeanum* cultivar ‘DaHongPao’ is renowned for its characteristic bright red pericarp during fruit maturation. Previous studies have suggested that flavonoids, such as anthocyanins, might be involved in the production of red pigments^38^. A single copy of anthocyanidin synthase (ANS), which catalyzes the key step in anthocyanin biosynthesis, was retained in both the *Arabidopsis* and *C. sinensis* genomes, whereas it was expanded to five copies in the *Z. bungeanum* genome (Fig. 5A). The expression levels of the five ANS genes increased continuously during the later stages of fruit development (Fig. 5B). In particular, the expression of EVM0019607.1 was dramatically increased in the last stage of pericarp development and was approximately 28-fold higher than the average expression of all genes. However, the unique ANS in *C. sinensis* was not expressed during pericarp development (Fig. 5B). The key positive regulator of anthocyanin biosynthesis *Ruby*1^39^, which encodes a MYB transcription factor, showed strongly divergent expression in *Z. bungeanum* and *C. sinensis*. Its orthologous genes in *Z. bungeanum* (EVM0033809.1 and EVM0052497.1) showed increased expression at the later stages of pericarp development (Fig. S18) compared with that of *C. sinensis*. Therefore, our integrative genomic and transcriptomic analyses suggested that changes in the gene expression and expansion of anthocyanidin synthase have shaped anthocyanin biosynthesis, resulting in the bright-red appearance of the pericarp during fruit maturation. In addition to anthocyanidin synthase, we also found that approximately 80% of the genes involved in flavonoid biosynthesis showed significantly higher expression in the pericarp of *Z. bungeanum* than in that of *C. sinensis* (Fig. 5C).

### Characteristics of terpene synthases (TPSs) in *Z. bungeanum*

**Fig. 6:**
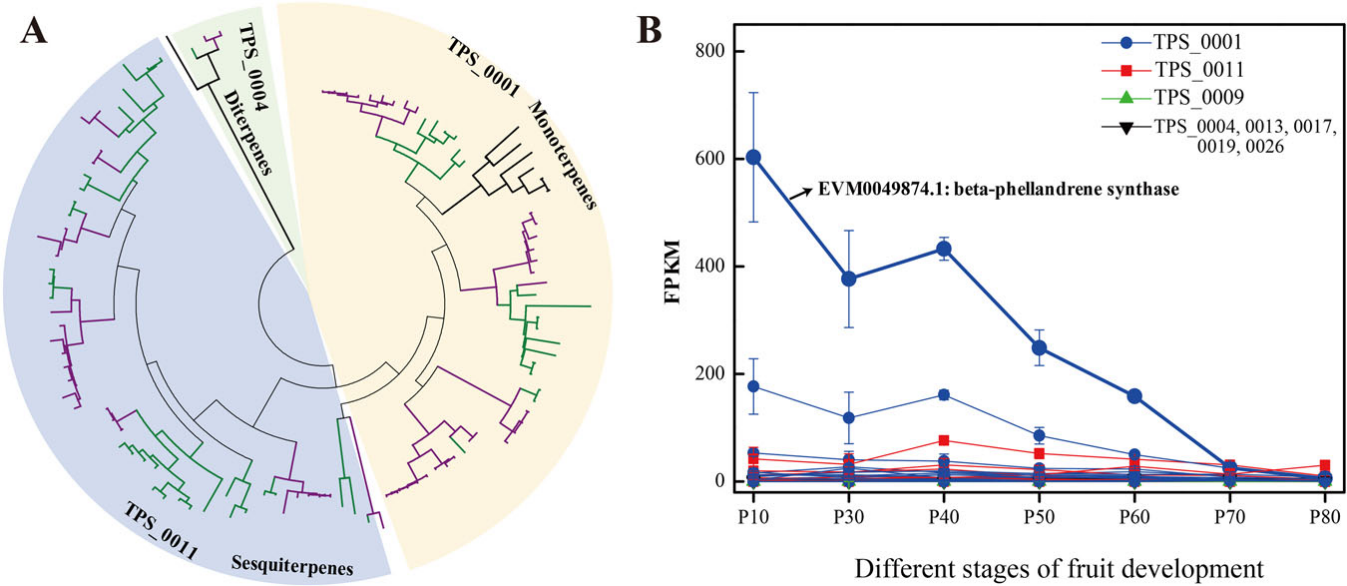
Evolutionary analysis and differentially expressed genes involved in the terpene synthase (TPS) pathway at seven fruit developmental stages in *Z. bungeanum*. **A** Gene expansion of the TPS_0001 and TPS_0011 gene family in *Z. bungeanum* (purple) and *C. sinensis* (green) compared to that in *A. thaliana* (black). The TPS_0004 gene family was used as the outgroup. **B** Expression levels of 70 TPSs in the *Z. bungeanum* genome at seven fruit developmental periods

Volatile oils, such as monoterpenes and sesquiterpenes, contribute to the characteristic aromas of *Zanthoxylum*^3,40^ and *Citrus*^41^ in Rutaceae. Most terpenes are produced by terpene synthases (TPSs). A total of 70 TPS genes, assigned to eight gene families, were identified in the *Z. bungeanum* genome (Fig. S19, Table S19). The families TPS_0001 (producing monoterpenes, 31 genes) and TPS_0011 (producing sesquiterpenes, 23 genes) were significantly expanded in *Z. bungeanum* and *C. sinensis* compared to *Arabidopsis* (Fig. 6A, Tables S20 and S21). Furthermore, expression profile analysis of these 70 TPS genes showed that the expression levels of monoterpenoid synthases (TPS_0001) and sesquiterpenoid synthases (TPS_0011) were obviously higher than those of the other TPSs (Fig. 6B, Table S20). In particular, the gene EVM0049874.1, which was identified as beta-phellandrene synthase in Japanese pepper (*Z. piperitum*)^42^, had the highest expression level among all TPSs (Fig. 6B). This result is consistent with the fact that beta-phellandrene is the major accumulated product in the secretory cavities of the leaf and pericarp. Additionally, a previous study reported that the gene expression of beta-phellandrene synthase was detected only during the early stages of cavity development, while the formation of volatile terpenes occurred at a constant rate throughout the expansion of secretory cavities^42^. Similarly, our study indicated that the expression level of the beta-phellandrene synthase gene was dramatically decreased at the fruit maturation stage (Fig. 6B). A similar pattern was also observed for the monoterpenoid synthases of *C. sinensis* (Fig. S20), which are mainly used to produce *D*-limonene in the pericarp. Previous studies have indicated that the down-regulation of *D*-limonene synthase in orange fruit can induce resistance against fungal diseases^43,44^.

## Discussion

Although several species of *Zanthoxylum* have a long history of cultivation and application in traditional Chinese medicine and are also popular as food additives, scientific research has been hampered by the absence of genetic resources. Here, we present a genome assembly for *Z. bungeanum*, which has a larger genome size than most sequenced plants. Assembling this genome was highly challenging due to its high heterozygosity (2.87%), striking TE expansion (~89%), and dramatically numerous chromosomes (68 chromosomes); nevertheless, our assembly covers 95.5% of the *Z. bungeanum* genome (~4.23 Gb). This assembled *Zanthoxylum* reference genome will reveal novel evolutionary events that have not been uncovered in related plant taxa until now.

The genome size was larger and there were more genes and chromosomes in *Z. bungeanum* than in most sequenced dicots. Phylogenetic analysis indicated that *Z. bungeanum* and *C. sinensis* probably diverged approximately 35.3 MYA, which is consistent with the divergence time, 36.5 to 37.7 MYA, estimated by nuclear and chloroplast genes, respectively^45^. It has been well documented that extensive amplification of TEs and WGD events have resulted in significant genome expansion in plants^46–48^. The *Z. bungeanum* genome underwent a recent lineage-specific η-WGD event at approximately 26.8 MYA, which distinguished the genome of *Zanthoxylum* from its close relative *Citrus*. Following WGD, the return to a genetically diploid state was associated with numerous chromosomal fissions and fusions, finally resulting in 68 structurally diverse chromosomes. However, the rediploidization processes could not conceal the WGD event, and a dosage of duplicated genes was retained. Therefore, this lineage-specific η-WGD event may have been involved in driving genome expansion, the proliferation of TEs, and chromosomal rearrangement.

Similar to garlic, whose percentage of repetitive elements (91.3%) is the highest among all sequenced plant genomes^49^, more than 89% (∼3.8 Gb) of the *Z. bungeanum* genome assembly is composed of different transposable elements (TEs), which is slightly higher than the TEs (∼81% of 3.3 Gb genome size) in hot pepper^50^. Clearly, rapid amplification of retrotransposons contributed much more to the genome expansion in *Z. bungeanum* (72.04%) than that in *C. sinensis* (18%) (Fig. 3B) but paralleled the genomic topology of the maize genome (75%)^48^. In contrast to other dicots^51,52^, *Copia* elements constituted the predominant component of LTR elements. This scenario is quite different from that of most sequenced dicots, such as hot pepper, in which *Gypsy* elements are the predominant components of LTR retrotransposons^36,50^, but is similar to that of *C. sinensis*^21^. Most TEs were greatly expanded in *Z. bungeanum* after the speciation event, and this species-specific process led to the large extant genome size of *Z. bungeanum*. On the other hand, active TEs might have triggered the occurrence of fission and fusion events in *Zanthoxylum* chromosomes^53^. Comparative analysis of *Copia* and *Gypsy* elements between *Z. bungeanum* and *C. sinensis* showed that the LTRs in the former were young and accumulated separately from those of *C. sinensis*, implying that active transposition of LTRs in *Zanthoxylum* occurred specifically after its split from *Citrus* species and that their expansions were also responsible for *Z. bungeanum* genome expansion. Overall, these results showed that a recent η-WGD event occurred, followed by a more recent burst of TE insertions. Therefore, the *Z. bungeanum*-specific WGD event combined with recent TE bursts contributed to the extraordinarily large genome size and the evolution of unique *Zanthoxylum* traits. In addition, frequent fusion/fission events have also destroyed the ancestral genome state, broken the chromosomes, and finally yielded a large number of reconnected chromosomes (Fig. 2C).

Several studies have confirmed that gene expansion can deeply reshape the breadth and abundance of secondary metabolites in plants^50,51^. Evolution of the capsaicinoid biosynthetic pathway in hot pepper involved multiple rounds of unequal duplication of key genes (i.e., capsaicin synthase) along with changes in their expression after speciation^36^, and this pattern also holds true in *Z. bungeanum*. In this study, we identified lineage-specific genes that likely control the quality of *Z. bungeanum*, in particular, genes encoding enzymes relevant to sanshool, anthocyanin, and beta-phellandrene biosynthesis. Our comparative analyses indicated an obvious expansion of genes encoding acyl-ACP thioesterase, NAF, ANS, and TPS, which tend to be coexpressed during fruit development. Therefore, gene expansion and subsequent neofunctionalization in the *Zanthoxylum* genome may be a major driving force for its peculiar biological characteristics. Additionally, by integrating genomic and transcriptomic analyses, we clarified the evolutionary processes of many enzymes involved in the biosynthetic pathways of specific secondary metabolites in *Z. bungeanum*, which are the factors determining the quality of *Z. bungeanum*.

The *Z. bungeanum* reference genome reported here offers unprecedented insights into the genome dynamics of the spice crop and will continue to provide a strong foundation for further studies not only on *Z. bungeanum* but also on other Rutaceae species. A combination of comparative genomics, metabolic engineering, and transgenic approaches will help reveal the molecular mechanisms of secondary metabolites, thereby expediting the processes of crop improvement in the future.

## Experimental procedures

### PacBio sequencing

An improved CTAB method was used to extract genomic DNA. Genomic DNA was sheared into 20 kb fragments using a g-TUBE device (Covaris Inc., Woburn, MO, USA). The sheared DNA was purified and concentrated using Agencourt Ampure XP beads (Beckman Coulter Inc., Pasadena, CA, USA) and further used for single-molecule real-time (SMRT) bell preparation according to the manufacturer’s protocol (Pacific Biosciences, Menlo Park, CA, USA; 20 kb template preparation kit) using the BluePippin size selection protocol (Sagescience, Beverly, MA, USA). After size selection, the isolated SMRT bell fractions were purified using Ampure XP beads, and then they were used for primer (V3) and polymerase (2.0) binding according to the manufacturer’s binding calculator (Pacific Biosciences). Single-molecule sequencing was performed on a PacBio Sequel system, and only the subreads equal to or longer than 500 bp were used for subsequent genome assembly.

### Illumina sequencing

We constructed seven libraries with 270 bp insert fragments for *Z. bungeanum* following Illumina’s protocol (Illumina, San Diego, CA, USA). The sequencing adapters and contaminated reads (mitochondrial, bacterial, and viral sequences) were removed from the raw Illumina reads by alignment to the NCBI-NR database using BWA v0.7.13^54^ with default parameters. FastUniq v1.1^55^ was used to remove the duplicated read pairs, and low-quality reads were filtered satisfying the following conditions: (1) reads with ≥10% unidentified nucleotides (N), (2) reads with >10 nucleotides aligned to the adapter, allowing ≤10% mismatches, and (3) reads with >50% bases having a Phred quality <5.

### Hi-C sequencing

According to the Hi-C procedure, nuclear DNA from the leaves of *Z. bungeanum* was cross-linked and then cut with the restriction enzyme *Dpn* II, leaving pairs of distally located but physically interacting DNA molecules attached to one another. The sticky ends of these digested fragments were biotinylated and then ligated to each other to form chimeric circles. Biotinylated circles, which are chimeras of the physically associated DNA molecules from the original cross-linking, were enriched, sheared, and sequenced using the Illumina HiSeq X Ten platform with 150 bp paired-end reads. As a result, we obtained a total of 486.7 Gb clean Illumina reads.

### Genome assembly

The full PacBio long reads were converted to fasta format. First, we used NextDenovo (v2.3) (https://github.com/Nextomics/NextDenovo) to generate a draft genome assembly with default parameters for PacBio reads only. We then used NextPolish (v2.0)^24^ to polish the draft genome with both long and short reads to obtain the corrected genome. This was followed by processing using purge_dups to purge the haplotigs and error-containing fragments. Subsequently, contigs were clustered with hierarchical clustering of the Hi-C data. To anchor scaffolds onto chromosomes, the Hi-C sequencing data were aligned to the assembly by BWA (aln mode) using the default parameters^54^, and valid contacts were detected. In total, 224,908,615 valid interaction read pairs were used for Hi-C scaffolding. Based on the valid Hi-C interaction read pairs, 16,615 contigs were clustered into 68 pseudochromosomes using ALLHiC^27,28^, of which 16,611 contigs with a total length of 4,124,904,629 bp were ordered and oriented within each group. The gap percentage in the final assembly was only 0.04%.

### Genome quality assessment

The completeness of the assembly was checked by mapping 2,270 benchmarking universal single-copy orthologs (BUSCOs) and 458 core eukaryotic genes (CEGs) to the genomes using BUSCO v3.0.2b^56^ and CEGMA v2.5^57^, respectively. Additionally, we used the LTR assembly index (LAI)^58^ to evaluate the completeness of the assembly.

### Repeated sequence prediction

The repeat components in *Z. bungeanum* assembly were first estimated by building a *de novo* repeat library by employing the programs LTR-FINDER^59^, MITE-Hunter^60^, RepeatScout v1.0.5^61^, and PILER-DF^62^, and the output results were merged together and classified using PASTEClassifier v1.0^63^. This *de novo* constructed database together with the Repbase database v20.01^64^ were used to create the final repeat library. Repeat sequences in *Z. bungeanum* were identified and classified using the RepeatMasker program v4.0.6^65^. The LTR family classification criterion was defined based on 5′ LTR sequences of the same family sharing at least 80% identity over at least 80% of their length. The expansion history of transposons was estimated by computing the divergence of the transposon *Copia* from the corresponding consensus sequence in the repeat library according to the RepeatMasker output and then calculating the percentage of transposons at different divergence levels.

### LTR-RT analysis

Long terminal repeat retrotransposons (LTR-RTs) were identified using LTR_retriever. We identified a total of 53,470 intact LTR-RTs (the output file with the name “.pass.list”). Then, we extracted the internal regions of all intact LTR-RTs and conducted BLASTX searches into the nonredundant LTR-RT library (.LTRlib.fa). By analyzing the best hits from all intact LTR-RTs to the nonredundant LTR-RT library, the internal regions of all intact LTR-RTs can map up to 3300 LTR-RTs in the nonredundant LTR-RT library.

### Protein-coding gene prediction

We used *de novo* protein homology and RNA-Seq approaches for protein-coding gene prediction. In detail, Genscan v1.0^66^, Augustus v2.5.5^67^, GlimmerHMM v3.0.1^68^, GeneID v1.3, and SNAP^69^ were used to perform *de novo* gene prediction; the alignment of the homologous peptides from *Arabidopsis thaliana* (The Arabidopsis Information Resource), *Oryza sativa* (Phytozome v12.1), and *Citrus reticulata* (http://citrus.hzau.edu.cn/orange/index.php) to our assemblies was used to identify homologous genes with GeMoMa v1.4.2^70^; the RNA-Seq reads were assembled into contigs and the *de novo* assembly yielding unigenes was performed using Trinity; and the resulting unigenes were aligned to the repeat-masked assemblies using BLAT^71^. Subsequently, the gene structures of the BLAT alignment results were modeled using PASA^72^, and the protein-coding regions were identified using TransDecoder v3.0.1 (https://github.com/TransDecoder/TransDecoder/) and GeneMarkS-T^73^. Finally, consensus gene models were generated by integrating *de novo* predictions, protein alignments, and transcript data using EVidenceModeler^74^. Annotation of the predicted genes was performed by BLAST searches against a series of nucleotide and protein sequence databases, including KOG^75^, KEGG^76^, NCBI-NR, and TrEMBL^77^, with an *E*-value cutoff of 1e-5. Gene Ontology (GO) for each gene was assigned by Blast2GO^78^ against the NCBI database.

### Noncoding RNA prediction

Noncoding RNAs play important roles in a variety of processes and include the genes encoding ribosomal RNAs (rRNAs), transfer RNAs (tRNAs), and microRNAs (miRNAs). The rRNA fragments were identified by aligning the rRNA template sequences against the Pfam database v32.0^79^ using BLAST with an *E-value* of 1e-10 and identity cutoff of 95% or more. The tRNAScan-SE algorithms^80^ with default parameters was applied to predict tRNA genes. The miRNA genes were predicted using INFERNAL v1.1^81^ against the Rfam database v14.0^82^ with a cutoff score of 30 or more. The minimum cutoff score was based on the settings that yielded a false-positive rate of 30 bits.

### Comparative genomics analyses

Protein sequences of *Z. bungeanum*, *Citrus sinensis*, *Arabidopsis thaliana*, *Amborella trichopoda*, *Piper nigrum*, *Zea mays*, *Oryza sativa*, *Papaver somniferum*, *Vitis vinifera*, *Dimocarpus longan*, *Brassica napus*, *Gossypium hirsutum*, *Arachis hypogaea*, *Cucumis sativus*, *Sesamum indicum*, *Capsicum annuum*, and *Nicotiana tabacum* were used for all BLASTP analyses. The results were analyzed using OrthoMCL software^83^ with an MCL inflation of 1.5 to identify gene family clusters. Single-copy gene clusters shared by all 17 species were used to construct a phylogenetic tree using PhyML v3.0^84^. The divergence time was estimated using the MCMCtree implemented in PAML package v4.9^85^. Calibration times were obtained from the TimeTree database (http://www.timetree.org/). Homologous blocks were detected using Mcscan v1.1^86^. The *K*_*s*_ values of the blocks were calculated using the HKY model^87^. According to the divergence time between *Z. bungeanum* and *C. sinensis* derived from the phylogenetic tree (Fig. 2A, 35.3 MYA), the synonymous substitution rate is 3.92 × 10^−9^ synonymous substitutions yr^−1^ (T = *Ks*/2λ and λ = 0.277/2 × 35.3 = 3.92E-9). The *Zanthoxylum*-specific WGD event date was obtained based on the synonymous (*K*_*s*_) substitutions calculation with λ = 3.92E-9.

Expansion and contraction of OrthoMCL-derived gene clusters was determined using CAFÉ v2.1 and was based on changes in gene family size in the inferred phylogenetic history. KEGG and GO annotations of the gene family were completed by aligning the genes to the KEGG database and NCBI nonredundant database using BLASTP with an *E* value of 1e-5. Blast2GO was used to obtain the associated GO terms. The enrichment score was defined as a hypergeometric test value.

### Synteny analysis

The genome synteny between and within species was analyzed via all-against-all BLASTP searches of protein sequences (with an *E*-value cutoff of 1e-5). Collinear blocks containing at least 10 genes (-s 10) and a maximum of 25 gaps (genes) between two proximal orthologs within a block (-m 25) were identified using Mcscan v1.1^86^. Synteny was searched for by comparing the *Z. bungeanum* genome with the genomes of *C. sinensis* and *V. vinifera*.

### Karyotype evolution analysis of Rutaceae

We performed collinearity analysis for each species within the set containing *Z. bungeanum*, *Xanthoceras sorbifolia*^88^, *C. sinensis*^21^ and *Arabidopsis*^89^ and *Vitis vinifera*^29^ using MCScanX^86^, and the syntenic blocks were identified based on all-versus-all BLAST alignments included in the JCVI package^90^ with default parameters. The distribution of seven ancestral eudicot chromosomal lineages for each chromosome in each species was depicted by the syntenic blocks between the ancestral chromosomes of grape^29^ as described in Bolot et al.^91^ and Murat et al.^92^ and those of the detected species. Speciation event dates were obtained based on the synonymous (*K*_*s*_) substitutions calculation (divergence time = *K*_*s*_/2 × r) with r = 6.5 × 10^−9^ (ref. ^93^).

### Coexpression analysis

Based on quality scores, the clean reads from the transcriptome data obtained from pericarps at seven developmental stages were trimmed using the quality trimming program Btrim^94^ and aligned to the *Z. bungeanum* reference assembly using TopHat v2.21^95^. Cufflinks v2.2.1^95^ was used to assemble the mapped reads for each sample. We used the fragments per kilobase of exon model per million mapped reads (FPKM) as the normalized gene expression level. We constructed a coexpression network using the cluster function in MATLAB. First, 2,752 metabolic genes (average FPKM > 5) were selected based on the KEGG annotation. The standard of FPKM > 5 was selected because the expression profile of genes with low expression is susceptible to sequencing errors. Second, based on the Spearman correlation between genes, the 2,752 metabolic genes were clustered into five subnetwork modules (Fig. 4C and Fig. S16). KEGG enrichment analysis was conducted for each module to understand the relationship between the enriched pathways and gene expression patterns. The *p values* were calculated by a hypergeometric test and adjusted using the Benjamini–Hochberg procedure.

### Comparison of transcriptomes between *Z. bungeanum* and *C. sinensis*

Gene expression in *C. sinensis* was referenced from a previous study^96^ in which transcriptome sequencing of the pericarps was performed at 90, 120, 150, 180, and 210 days after full bloom. To determine the gene expression differences between *Z. bungeanum* and *C. sinensis* pericarps, we first identified 14,675 orthologous genes between the two species. Then, according to gene expression levels with equal medians in the 14,675 orthologs of the two species, the gene expression of *C. sinensis* was normalized by dividing by 4.78.

### Analysis of gene family expansion

The protein families that expanded in *Z. bungeanum* compared to *C. sinensis* were considered to be expanded in *Zanthoxylum* (*p* < 0.05). The KEGG annotations of *C. sinensis* and *A. thaliana* were downloaded from the KEGG website (https://www.genome.jp/kegg/). KEGG annotation of *Z. bungeanum* was performed using the KEGG Automatic Annotation Server (KAAS) platform. The acyl-ACP thioesterases and acetyltransferases in *Z. bungeanum*, *C. sinensis*, and *A. thaliana* were predicted using hmmsearch in conjunction with the acetyltransferase and thioesterase family hmm models PF01643 and PF02458 (*E*-value < 1e-6) from Pfam^79,97^. Then, we tested whether the gene number of the two gene families in one plant was significantly higher than that in another plant by comparing the background gene number between the two plants. The *p values* were calculated by a hypergeometric test and adjusted using the Benjamini–Hochberg procedure.

### Annotation and analysis of terpene synthases

The TPSs in the *Z. bungeanum* genome were predicted using hmmsearch in conjunction with the terpene synthase family hmm model PF03936 (*E*-value < 1e-6) from Pfam^78,96^. To analyze the evolution of the TPS gene family in *Z. bungeanum*, *C. sinensis*, and *A. thaliana*, the 158 (70 + 55 + 33) TPS proteins were further classified into 10 TPS families and 41 TPS subfamilies (Table S21) based on three criteria: (1) the proteins in a family or subfamily had relatively closer phylogenetic relationships in the phylogenetic tree constructed by the alignment of all TPS proteins; (2) the identity between two protein sequences in a family was higher than 45%; and (3) the identity between two protein sequences in a subfamily was higher than 60%.

### Accession numbers

The *Z. bungeanum* genome, annotation, and raw data are deposited in NCBI under BioProject ID PRJNA524242 and accession number SKCR00000000.

## Supplementary information

Supplemental tables for manuscript

Supplemental text and figures for manuscript

Data Set 1
