# Supplementary material for: Zanthoxylum-specific whole genome duplication and recent activity of transposable elements in the highly repetitive paleotetraploid Z. bungeanum genome
Source: Hortic Res. 2021 Sep 3;8:205. doi: 10.1038/s41438-021-00665-1 (PMC8417289; doi:10.1038/s41438-021-00665-1)
Supplement: Supplementary file 1 — Supplemental tables for manuscript [file 41438_2021_665_MOESM1_ESM.doc]

**Supplemental Tables**

**Table S1 Statistics of PacBio reads**

| **Classes** | **Statistics** |
| --- | --- |
| **Read number** | 45,671,445 |
| **Total base (bp)** | 429,555,939,058 |
| **Read_N50 (bp)** | 14,456 |
| **Mean length (bp)** | 9,405 |
| **Maximum length (bp)** | 242,857 |

**Table S2 Statistics of Illumina sequencing**

| **Library** | **Data (Gb)** | **Depth (×)** | **Q20 (%)** | **Q30 (%)** |
| --- | --- | --- | --- | --- |
| 270 bp_1 | 30.12 | 8.37 | 96.78 | 93.04 |
| 270 bp_2 | 34.09 | 9.47 | 96.70 | 92.86 |
| 270 bp_3 | 32.07 | 8.91 | 96.65 | 92.75 |
| 270 bp_4 | 29.65 | 8.24 | 96.53 | 92.47 |
| 270 bp_5 | 19.00 | 5.28 | 96.52 | 92.49 |
| 270 bp_6 | 31.11 | 8.64 | 96.46 | 92.38 |
| 270 bp_7 | 37.77 | 10.49 | 96.52 | 92.49 |

**Table S4 Protein-coding gene prediction**

| **Method** | **Software** | **Gene number** |
| --- | --- | --- |
| *Ab initio* | Genscan | 72,220 |
|  | Augustus | 81,302 |
|  | GlimmerHMM | 136,640 |
|  | GeneID | 116,941 |
|  | SNAP | 115,759 |
| Homology-based | GeMoMa | 80,594 |
|  |  | 97,478 |
|  |  | 77,491 |
| RNAseq | PASA | 79,052 |
|  | TransDecoder | 196,915 |
|  | GeneMarkS-T | 123,097 |
| Integration | EVM | 74,307 |

**Table S5 Annotation of protein-coding genes**

| **Database** | **Annotated number** | **Percentage (%)** |
| --- | --- | --- |
| GO | 44,811 | 60.31% |
| KEGG | 28,995 | 39.02% |
| KOG | 41,392 | 55.70% |
| TrEMBL | 67,819 | 91.27% |
| NR | 73,261 | 98.59% |
| All | 73,633 | 99.09% |

**Table S6 CEGMA estimation**

| **Species** | **Number of 458 CEGs* present in assembly** | **% of 458 CEGs present in assemblies** | **Number of 248 highly conserved CEGs present** | **% of 248 highly conserved CEGs present** |
| --- | --- | --- | --- | --- |
| *Z. bungeanum* | 448 | 97.82% | 228 | 91.94% |

**Table S7 BUSCO estimation**

| **Species** | **Complete BUSCOs** | **Complete and single-copy BUSCOs** | **Complete and duplicated BUSCOs** | **Fragmented BUSCOs** | **Missing BUSCOs** |
| --- | --- | --- | --- | --- | --- |
| *Z. bungeanum* | 2270 | 488 | 1782 | 10 | 46 |

**Table S8 Statistics of gene family clustering from 17 species**

| **Species** | **Total gene** | **One Copy^1^** | **Multi Copy^2^** | **Unigene^3^** | **Other gene^4^** | **Cluster^5^** | **Un-**  **Cluster^6^** | **Total family^7^** | **Unique family^8^** |
| --- | --- | --- | --- | --- | --- | --- | --- | --- | --- |
|
| *A. trichopoda* | 26,846 | 4,628 | 2,883 | 4,578 | 6,256 | 18,345 | 8,501 | 11,917 | 1,104 |
| *P. nigrum* | 62,672 | 2,170 | 12,319 | 17,311 | 12,302 | 44,102 | 18,570 | 15,695 | 4,786 |
| *B. napus* | 56,055 | 2,145 | 13,028 | 8,185 | 16,920 | 40,278 | 15,777 | 17,546 | 2,209 |
| *C. sativus* | 24,035 | 4,077 | 4,851 | 1,287 | 8,159 | 18,374 | 5,661 | 12,724 | 421 |
| *D. longan* | 39,282 | 3,838 | 6,419 | 8,492 | 14,629 | 33,378 | 5,904 | 14,675 | 1,173 |
| *A. hypogaea* | 41,518 | 3,574 | 6,778 | 10,869 | 10,795 | 32,016 | 9,502 | 13,491 | 1,970 |
| *G. hirsutum* | 38,784 | 2,568 | 11,443 | 3,841 | 14,720 | 32,572 | 6,212 | 14,983 | 1,128 |
| *S. indicum* | 23,589 | 3,231 | 7,523 | 952 | 10,138 | 21,844 | 1,745 | 12,789 | 276 |
| *Z. mays* | 39,591 | 2,834 | 9,350 | 6,339 | 13,266 | 31,789 | 7,802 | 15,731 | 1,906 |
| *C. sinensis* | 29,067 | 4,034 | 5,296 | 3,050 | 12,145 | 24,525 | 4,542 | 14,835 | 705 |
| *C. annuum* | 35,845 | 3,576 | 6,999 | 4,939 | 12,156 | 27,670 | 8,175 | 14,515 | 1,004 |
| *O. sativa* | 35,775 | 3,819 | 5,819 | 3,584 | 10,612 | 23,834 | 11,941 | 15,125 | 1,249 |
| *N. tabacum* | 61,410 | 344 | 20,678 | 7,171 | 26,871 | 55,064 | 6,346 | 16,373 | 1,502 |
| *A. thaliana* | 27,412 | 3,507 | 6,839 | 1,039 | 12,408 | 23,793 | 3,619 | 15,472 | 351 |
| ***Z. bungeanum*** | **74,307** | **341** | **30,839** | **4,498** | **33,576** | **69,254** | **5,053** | **16,528** | **1,693** |
| *V. vinifera* | 29,927 | 3,858 | 6,005 | 2,055 | 10,527 | 22,445 | 7,482 | 13,941 | 695 |
| *P. somniferum* | 62,883 | 385 | 18,273 | 19,531 | 20,257 | 58,446 | 4,437 | 14,709 | 3,019 |

**Note:** ^1^only one gene coming from one species within a family (the family includes 17 species); ^2^at least two genes coming from one species within a family (the family includes 17 species); ^3^genes within a family only coming from one species (the family includes one species); ^4^one or multiple genes coming from one species within a family (the family includes less than 17 species); ^5^genes involving in clustering; ^6^genes without involving in clustering; ^7^family number; ^8^unique family number

**Table S9 KEGG functional enrichment analysis of *Z. bungeanum*-specific gene families (*P* < 0.05)**

| #Kegg_pathway | ko_id | Cluter  frequency | Genome  frequency | *P*-value |
| --- | --- | --- | --- | --- |
| Ubiquitin mediated proteolysis | ko04120 | 1.09% | 0.59% | 9.43E-09 |
| Ribosome | ko03010 | 1.93% | 1.48% | 4.24E-06 |
| Protein processing in endoplasmic reticulum | ko04141 | 1.22% | 0.96% | 0.000103 |
| Phagosome | ko04145 | 0.64% | 0.42% | 0.000247 |
| Photosynthesis - antenna proteins | ko00196 | 0.24% | 0.10% | 0.000356 |
| C5-Branched dibasic acid metabolism | ko00660 | 0.16% | 0.04% | 0.000409 |
| Terpenoid backbone biosynthesis | ko00900 | 0.40% | 0.24% | 0.00166 |
| Peroxisome | ko04146 | 0.53% | 0.38% | 0.00233 |
| Biosynthesis of unsaturated fatty acids | ko01040 | 0.20% | 0.11% | 0.00984 |
| Spliceosome | ko03040 | 0.89% | 0.80% | 0.0101 |
| RNA polymerase | ko03020 | 0.24% | 0.16% | 0.0241 |
| Valine, leucine and isoleucine biosynthesis | ko00290 | 0.16% | 0.10% | 0.0431 |

**Table S10 KEGG functional enrichment analysis of the expanded gene families in *Z. bungeanum* genome (*P* < 0.05)**

| #Kegg_pathway | ko_id | Cluter  frequency | Genome  frequency | *P*-value |
| --- | --- | --- | --- | --- |
| Starch and sucrose metabolism | ko00500 | 4.16% | 2.90% | 2.24E-26 |
| Plant hormone signal transduction | ko04075 | 5.62% | 4.14% | 7.02E-26 |
| Cyanoamino acid metabolism | ko00460 | 1.13% | 0.60% | 3.83E-23 |
| Phenylpropanoid biosynthesis | ko00940 | 3.31% | 2.39% | 1.29E-17 |
| Stilbenoid, diarylheptanoid and gingerol biosynthesis | ko00945 | 0.86% | 0.49% | 6.03E-15 |
| Ribosome | ko03010 | 5.71% | 4.57% | 1.13E-14 |
| Plant-pathogen interaction | ko04626 | 2.91% | 2.20% | 9.37E-12 |
| Isoquinoline alkaloid biosynthesis | ko00950 | 0.61% | 0.34% | 2.01E-11 |
| Amino sugar and nucleotide sugar metabolism | ko00520 | 2.34% | 1.81% | 1.43E-08 |
| Glucosinolate biosynthesis | ko00966 | 0.25% | 0.12% | 6.32E-08 |
| Linoleic acid metabolism | ko00591 | 0.31% | 0.17% | 2.26E-07 |
| Galactose metabolism | ko00052 | 1.04% | 0.73% | 2.87E-07 |
| Flavonoid biosynthesis | ko00941 | 0.90% | 0.63% | 8.29E-07 |
| alpha-Linolenic acid metabolism | ko00592 | 0.97% | 0.69% | 9.77E-07 |
| mRNA surveillance pathway | ko03015 | 2.52% | 2.03% | 1.05E-06 |
| Phenylalanine metabolism | ko00360 | 0.85% | 0.60% | 2.15E-06 |
| ABC transporters | ko02010 | 0.57% | 0.38% | 1.44E-05 |
| Circadian rhythm - plant | ko04712 | 0.85% | 0.62% | 2.54E-05 |
| Ether lipid metabolism | ko00565 | 0.51% | 0.36% | 1.57E-04 |
| Limonene and pinene degradation | ko00903 | 0.26% | 0.15% | 1.63E-04 |
| Tryptophan metabolism | ko00380 | 0.54% | 0.39% | 3.03E-04 |
| Diterpenoid biosynthesis | ko00904 | 0.52% | 0.37% | 4.06E-04 |
| Sesquiterpenoid and triterpenoid biosynthesis | ko00909 | 0.28% | 0.18% | 9.93E-04 |
| Glycolysis / Gluconeogenesis | ko00010 | 2.00% | 1.74% | 2.85E-03 |
| Anthocyanin biosynthesis | ko00942 | 0.06% | 0.03% | 3.87E-03 |
| Cysteine and methionine metabolism | ko00270 | 1.64% | 1.43% | 0.007316 |
| Lysine degradation | ko00310 | 0.48% | 0.37% | 0.0083318 |
| Acridone alkaloid biosynthesis | ko01058 | 0.06% | 0.02% | 0.0085548 |
| Tropane, piperidine and pyridine alkaloid biosynthesis | ko00960 | 0.41% | 0.32% | 0.0134302 |
| Alanine, aspartate and glutamate metabolism | ko00250 | 0.81% | 0.68% | 0.0149978 |
| Cutin, suberine and wax biosynthesis | ko00073 | 0.39% | 0.31% | 0.0233626 |
| Tyrosine metabolism | ko00350 | 0.68% | 0.57% | 0.0237761 |
| Fructose and mannose metabolism | ko00051 | 0.91% | 0.78% | 0.0283325 |
| Synthesis and degradation of ketone bodies | ko00072 | 0.16% | 0.11% | 0.0308812 |

| #Kegg_pathway | ko_id | Cluter_  frequency | Genome_  frequency | *P*-value |
| --- | --- | --- | --- | --- |
| Proteasome | ko03050 | 1.06% | 0.87% | 2.74E-07 |
| mRNA surveillance pathway | ko03015 | 2.26% | 2.01% | 1.06E-05 |
| Carbon fixation in photosynthetic organisms | ko00710 | 1.07% | 0.92% | 4.33E-05 |
| Pyruvate metabolism | ko00620 | 1.32% | 1.16% | 0.000109 |
| Endocytosis | ko04144 | 2.57% | 2.36% | 0.000282 |
| AGE-RAGE signaling pathway in diabetic complications | ko04933 | 0.28% | 0.22% | 0.000939 |
| One carbon pool by folate | ko00670 | 0.34% | 0.28% | 0.002814 |
| Arginine and proline metabolism | ko00330 | 0.87% | 0.77% | 0.004569 |
| Plant hormone signal transduction | ko04075 | 4.30% | 4.09% | 0.004906 |
| Glycerophospholipid metabolism | ko00564 | 1.31% | 1.20% | 0.009033 |
| Arachidonic acid metabolism | ko00590 | 0.18% | 0.14% | 0.009108 |
| Other types of O-glycan biosynthesis | ko00514 | 0.10% | 0.07% | 0.014194 |
| Phenylalanine, tyrosine and tryptophan biosynthesis | ko00400 | 0.73% | 0.65% | 0.015263 |
| Fatty acid metabolism | ko01212 | 1.04% | 0.95% | 0.01968 |
| Protein export | ko03060 | 0.78% | 0.71% | 0.019815 |
| Porphyrin and chlorophyll metabolism | ko00860 | 0.63% | 0.56% | 0.021556 |
| Ether lipid metabolism | ko00565 | 0.40% | 0.35% | 0.02221 |
| Glycolysis / Gluconeogenesis | ko00010 | 1.82% | 1.72% | 0.031174 |
| Glycosphingolipid biosynthesis - globo series | ko00603 | 0.25% | 0.21% | 0.033606 |
| Biosynthesis of unsaturated fatty acids | ko01040 | 0.37% | 0.32% | 0.034989 |
| RNA transport | ko03013 | 2.56% | 2.45% | 0.048272 |
| Terpenoid backbone biosynthesis | ko00900 | 0.80% | 0.74% | 0.053525 |

**Table S12 KEGG functional enrichment analysis of the WGD duplicated genees in *Z. bungeanum* genome** **(*P* < 0.05)**

***Table S13 Genome size and repeat content of Z. bungeanum and C. sinensis***

| **Species** | **Assemble Length** | **Repeats identified** | ***Gypsy*** | ***Copia*** |
| --- | --- | --- | --- | --- |
| *Z. bungeanum* | 4.23 Gb | 89.14% | 29.00% | 43.04% |
| *C.* *sinensis* | 367 Mb | 20.50% | 9.77% | 7.84% |

**Table S14 Annotation of repeat sequences in *Z. bungeanum*** genome

| **Classification** | **Length (bp)** | **Copy number** | **Percentage of genome (%)** |
| --- | --- | --- | --- |
| Class I: Retrotransposon elements | 3,577,869,595 | 4,087,097 | 84.48 |
| LTR-Retrotransposon | 3,034,335,027 | 2,461,787 | 72.04 |
| *Copia* | 1,822,716,648 | 1,433,948 | 43.04 |
| *Gypsy* | 1,228,229,823 | 1,085,996 | 29.00 |
| LARD | 700,289,950 | 1,249,981 | 16.54 |
| DIRS | 225,779,059 | 182, 935 | 5.33 |
| TRIM | 4,156,191 | 8,607 | 0.1 |
| LINE | 30,503,298 | 69,566 | 0.72 |
| SINE | 6,741 | 1,093,263 | 0.03 |
| PLE | 11,333,232 | 15,866 | 0.27 |
| TRIM | 10,353,801 | 19,186 | 0.24 |
| Class II: DNA transposon elements | 172,192,983 | 341,386 | 4.19 |
| TIR | 166,580,797 | 342,744 | 3.93 |
| Helitron | 7,420,247 | 14,931 | 0.18 |
| Maverick | 798,067 | 1,257 | 0.02 |
| MITE | 1,836,294 | 5,865 | 0.04 |
| Other repeats |  |  |  |
| Potential Host Gene | 13,270,047 | 46,787 | 0.31 |
| Simple_repeats | 2,605,387 | 4,633 | 0.06 |
| Unknown | 183,707,040 | 418,970 | 4.34 |
| Total Content | 3,775,116,687 | 4,926,808 | 89.14 |

**Table S16 The significantly expanded gene families and pathways in *Z. bungeanum* compared to *C. sinensis***

| **Families and Pathways** | **Zbun** | **Zbun_BG** | **Csin** | **Csin_BG** | ***P*-value (Zbun-Csin)** |
| --- | --- | --- | --- | --- | --- |
| PF01643_Acyl-ACP thioesterase | 33 | 47479 | 4 | 18905 | 0.009 |

**Table S20 Comparative analysis of TPS subfamilies in *Z. bungeanum*, *C. sinensis* and *A. thaliana***

| **Family** | **Atha** | **Atha_BG** | **Zbun** | **Zbun_BG** | **Csin** | **Csin_BG** | ***P*-value (Zbun-Atha)** | ***P*-value (Csin-Atha)** |
| --- | --- | --- | --- | --- | --- | --- | --- | --- |
| PF03936_TPS_0001 | 6 | 33 | 31 | 70 | 20 | 55 | 0.0079073 | 0.05638 |
| PF03936_TPS_0011 | 0 | 33 | 23 | 70 | 26 | 55 | 3.35E-05 | 2.44E-07 |

**Table S22 The abbreviation of enzymes involved in sanshool biosynthesis**

| **Abbr** | **Function** |
| --- | --- |
| Acc | Acetyl-CoA carboxylase |
| FabD | [acyl-carrier-protein] S-malonyltransferase |
| FabH | 3-oxoacyl-[acyl-carrier-protein] synthase III |
| FabF | 3-oxoacyl-[acyl-carrier protein] reductase |
| FabZ | 3-hydroxyacyl-[acyl-carrier-protein] dehydratase |
| FabI | enoyl-[acyl-carrier protein] reductase I |
| FATB | fatty acyl-ACP thioesterase B |
| FATA | fatty acyl-ACP thioesterase A |
| FAB2 | acyl-[acyl-carrier-protein] desaturase |
| FAD3 | acyl-lipid omega-3 desaturase |
| SLD | sphingolipid 8-(E)-desaturase |
| FAD2 | omega-6 fatty acid desaturase |
| DEGS; | sphingolipid 4-desaturase |
| FAD6 | acyl-lipid omega-6 desaturase |
| SCD | stearoyl-CoA desaturase |
| ACSL | long-chain acyl-CoA synthetase |
| ilvH | acetolactate synthase I/III small subunit |
| ilvC | ketol-acid reductoisomerase |
| ilvD | dihydroxy-acid dehydratase |
| ilvE | branched-chain amino acid aminotransferase |
| VDC | Valine decarboxylase |
| PyDC | Pyridoxal-dependent decarboxylase |
| ADC | Adenosylmethionine decarboxylase |
| PDC | Phosphatidylserine decarboxylase |
| LDC | Possible lysine decarboxylase |
| BAHD_AT | BAHD acyltransferase |
| GNAT | Gcn5-related N-acetyltransferase |
