# Supplementary material for: Zanthoxylum-specific whole genome duplication and recent activity of transposable elements in the highly repetitive paleotetraploid Z. bungeanum genome
Source: Hortic Res. 2021 Sep 3;8:205. doi: 10.1038/s41438-021-00665-1 (PMC8417289; doi:10.1038/s41438-021-00665-1)
Supplement: Supplementary file 2 — Supplemental text and figures for manuscript [file 41438_2021_665_MOESM2_ESM.doc]

Supplement for

***Zanthoxylum-*specific whole genome duplication and recent activity of transposable elements in the highly repetitive paleotetraploid *Z. bungeanum* genome**

Shijing Feng^1,4†^, Zhenshan Liu^2†^, Jian Cheng^3†^, Zihe Li^5†^, Lu Tian^1,4^, Min Liu^6^, Tuxi Yang^1,4^, Yulin Liu^1,4^, Yonghong Liu^1,4^, He Dai^6^, Zujun Yang^8^, Qing Zhang^7^, Gang Wang^7^, Jisen Zhang^7*^, Huifeng Jiang^3*^and Anzhi Wei^1,4*^

*^1^**College of Forestry, Northwest A&F University, Yangling 712100, Shaanxi, China.*

*^2^College of Life Science, Northwest A&F University, Yangling 712100, Shaanxi, China.*

*^3^Key Laboratory of Systems Microbial Biotechnology, Tianjin Institute of Industrial Biotechnology, Chinese Academy of Sciences, Tianjin 300308, China.*

*^4^Research Centre for Engineering and Technology of Zanthoxylum State Forestry Administration, Yangling 712100, Shaanxi, China*

*^5^School of Ecology and Environment, Northwestern Polytechnical University, Xi’an, Shanxi, 710072, China.*

*^6^Biomarker Technologies Corporation, Beijing, China.*

*^7^Center for Genomics and Biotechnology, Haixia Institute of Science and Technology, Fujian Provincial Key Laboratory of Haixia Applied Plant Systems Biology, College of Life Sciences, Fujian Agriculture and Forestry University, Fuzhou 350002, China.*

*^8^Center for Information in Biology, College of Life Science and Technology, University of Electronic Science and Technology of China, 610054, Chengdu, China.*

^†^These authors contributed equally to this work.

^*^Correspondence and requests for materials should be addressed to Z.J. ([zjisen@126.com](mailto:zjisen@126.com)), H.J. (email: [jiang_hf@tib.cas.cn](mailto:jiang_hf@tib.cas.cn)) or to A.W. (email: [weianzhi@126.com](mailto:weianzhi@126.com)).

# 1.1 The history of *Zanthoxylum bungeanum*

Chinese pepper (*Zanthoxylum bungeanum* Maxim), known as “huajiao” in China, is widely used as a spice because of the distinctive tingling taste caused by alkamides and a traditional Chinese herbal medicine for the treatment of abdominal pain, toothache, dyspepsia, vomiting, ascariasis, etc., as recorded in many classic monographs of medicine including *ShenNongBenCaoJing* (ShenNong’s Classic of Materia Medica, the earliest Traditional Chinese Medicine monograph written between about 200 and 250 AD), *Mingyi Bielu*, *Zhenglei Bencao* and *Bencao Gangmu* (Compendium of Materia Medica) and the Pharmacopoeia of the People’s Republic of China^1-3^.

*Z. bungeanum* is believed to be native to China, and the first description of this plant can be traced back to *Shijing* (Classic of Poetry) 2,600 years ago. Ancient Chinese in the period of pre-Qin Dynasty always regarded the fruits as a precious token and used for worshiping ancestors and praying for more children and a good harvest, with this being the earliest utilization of *Z. bungeanum* in folk life. The main distribution of wild *Z. bungeanum* during the period of pre-Qin Dynasty covered the north Sichuan, south Gansu, southwest Shaanxi, south Shanxi, southeast Henan and the mountain regions of Hunan and Hubei, according to *Shijing*, *Fanzi Jiran* (Master Fan's Questions to Jiran, an ancient agricultural treatise), *Shan Hai Jing* (Classic of Mountains and Seas), Chu state poet Qu Yuan and many fruits excavated in these regions. Afterwards, with the improvement of market consumed, the origin of cultivation of *Z. bungeanum* occurred in Sichuan where a number of improved variety resources were available, at least 1,500 years ago in Jin Dynasty, as recorded in *Mingyi Bielu and Qimin Yaoshu* (Essential Techniques for the Welfare of the People, an ancient Chinese agricultural text written by the Northern Wei Dynasty official Jia Sixie). This was evident from the famous story that the Jin Dynasty royalty Wang Kai vied for the honor of being the richest with the fruits of *Z. bungeanum* as wealth. The Sichuan pepper has been a tribute for the past royal emperorship since Tang Dynasty. From then on to Ming Dynasty, the cultivation regions of *Z. bungeanum* extended to most provinces in China and the current distribution pattern gradually formed.

So far, numerous varieties of “huajiao” have been cultivated during the long-term domestication process, such as “Dahongpao”, “Xiaohongpao”, “Huangjinjiao”, “Yuexigongjiao”, and so on^4^. Distinct regional demands for different cultivars reflect local idiosyncrasies in consumer tastes: the cultivars of *Z. bungeanum* distributed within the tropical and the subtropical regions south of Qinling Mountains contain more numb-taste components but fewer leaf glandular punctate than that north of Qinling Mountains. Nowadays, *Z. bungeanum* is commercially cultivated on more than 1.7 million hectares of land on a national-wide scale, and 3.5 million tons of dried pericarp were produced annually (China economic and social development statistical database) with $12.0 per kilogram (https://www.huajiao.cn/), accounting for 4.0 billion dollars. *Z. bungeanum* is a drought-tolerant economic tree with a vital role in the livelihoods of millions of people in mountainous regions of Sichuan, Shaanxi, Yunnan, Guizhou, Gansu, etc., and in several other countries and regions including Japan, Korea and India.

# 1.2 Experimental procedures

### 1.2.1 Plant materials

For genome sequencing, we collected fresh and healthy leaves from an elite cultivar, ‘DaHongPao’, of *Z. bungeanum* grown in the Experimental Station of *Zanthoxylum* in Fengxian, which is located in the Shaanxi province of China. Leaf tissue was used for genome library preparation, and samples from five different tissues (roots, leaves, young stems, flowers and fruits) were used for RNA sequencing to enable genome annotation. In order to investigate the potential molecular mechanism associated with *Z. bungeanum* fruit traits, samples of fresh pericarps at seven fruit developmental stages were collected from 12 individual plants at 10, 30, 40, 50, 60, 70, and 80 days post-anthesis. Fresh and healthy tissues were immediately transferred into liquid nitrogen after collection and stored at ˗80 °C in the laboratory until DNA and RNA extraction.

### 1.2.2 Chromosome preparation

The protocol for mitotic metaphase chromosomes was modified slightly from that of a previous report^5^. Excised leaf primordia less than 5 mm from adult plants were treated with nitrous oxide gas for 2.5 h under pressure under 1.1 MPa. Treated root tips were fixed in ice-cold 90% acetic acid for 10 min and stored in 70% ethanol at -20°C until use. After washing in water on ice, the section of root tips containing dividing cells was dissected and digested in 20 L of 1% pectolyase Y23 (Yakult Pharmaceutical, Japan) and 2% cellulose Onozuka R-10 (Yakult Pharmaceutical) solution for 120 min at 37°C. After digestion, the root sections were washed once in 70% ethanol and once in 100% ethanol. The meristematic region of root samples was carefully broken using a needle and was vortexed at maximum speed in 100% ethanol for 30s at room temperature to separate cells. The cells were collected by centrifugation and re-suspended in a 90% acetic acid solution. The cell suspension was dropped onto glass slides in a box lined with wet paper.

### 1.2.3 Sequential fluorescence in situ hybridization (FISH)

The fluorescence in situ hybridization (FISH) protocol for identifying individual chromosomes was mainly based on our recent study^6^. In brief, the oligo-nucleotide probes Oligo-Telo (TTAGGGTTAGGGTTAGGGTTAGGG), Oligo-(GAA)_7_, and Oligo-5SrDNA (GTACTACTCTCGCCCAAGCACGCTTAACTTCGGAGTTC) were synthesized by Shanghai Invitrogen Biotechnology Co., Ltd. (Shanghai, China). In addition, we also conducted the hybrid experiment using four 18SrDNA sequences as probes. The oligonucleotide probe for 18SrDNA were as follows: 18SrDNA-1(GTAGCCGTTTCTCAGGCTCCCTCTCCGG), 18SrDNA-2 (ACCGCG

GCTGCTGGCACCAGACTTGCCC), 18SrDNA-3 (CTGAAACTTAAAGGAATTG

ACGGAAGG), 18SrDNA-4 (CCACCAACTAAGAACGGCCATGCACCACC). The synthetic oligonucleotides Oligo-Telo, Oligo-5SrDNA and Oligo-18SrDNA were 5ʹ end-labeled with 6-carboxyfluorescein (FAM), for green signal, and 6-carboxytetramethylrhodamine (Tamra), for red signal, respectively. The probe solution (20 ng/L in 2 × SSC and 1 × TE buffer, pH 7.0) was denatured for 5 min in boiling water and then placed on ice. A 6 μL probe solution was used for each slide. Hybridization was performed overnight at 37 ℃ in a humid chamber. After hybridization, the slides were washed using 2 × SSC and then mounted with Vectashield mounting medium containing 1.5 μg/mL 4′, 6-diamidino-2-phenylindole (DAPI; Vector Laboratories, Burlingame, CA, USA). Images were captured using an Olympus BX-51 microscope equipped with a DP-70 CCD camera.

### 1.2.4 Genome size estimation

Tender leaves were collected from the sequenced plant and analyzed using a flow cytometer to estimate the genome size. Approximately 20 mg of young leaves were placed in the center of a plastic Petri dish and chopped with a new razor blade in 1.0 mL of ice-cold nuclei isolation buffer (45 mM MgCl_2_·6H_2_O, 20 mM MOPS, 30 mM sodium citrate, 1% (w/v) PVP40, 0.2% (v/v) Tritonx-100, 10 mM Na_2_EDTA, 20 μL/mL β-mercaptoethanol, pH 7.0, stored at -20 °C and thawed to 4 °C). The homogenate was mixed by pipetting up and down several times to avoid air bubbles, and then filtered through a 42 μm nylon mesh into a labeled sample tube. Subsequently, the stock solution of the DNA fluorochrome was added to the sample tube, and the tube was gently shaken. Simultaneously, 50 μg/mL each of propidium iodide (PI) and RNase were added. The samples were incubated on ice (a few minutes to 1 h) before analysis with occasional shaking. Finally, the relative fluorescence of the stained nuclei was measured using a BD FACSCaliber flow cytometer (Becton, Dickinson and Company, Franklin Lakes, NY, USA) to estimate the genome size. The nuclear DNA content of samples (G1 phase) was estimated according to the equation: G_unknown_ = ((G1_PI_fluor_unknown_)/(G1_PI_fluor_standard_)) × G_standard_, where G_unknown_ and G_standard_ refer to the genome size of *Z. bungeanum* and reference species (maize and barley in this study), respectively, and G1_PI_fluor_unknown_ and G1_PI_fluor_standard_ refer to the channel number of PI fluorescence of *Z. bungeanum* and reference species during G1 phase, respectively. Maize as standard: (210.08/115.78) × 2.3 Gb = 4.17 Gb; Pea as standard: (211.27/200.54) × 4.45 Gb = 4.68 Gb. The average genome size was (4.17 Gb + 4.68 Gb)/2 = 4.43 Gb.

we further performed *k*-mer analysis with Illumina sequencing short reads to estimate the genome size. Initially, we generated the 21-mer occurrence distribution of sequencing reads from short libraries (≤500 bp) using Jellyfish (version 2.1.3)^7^. We then calculated the genome size based on the formula: genome size = *k*-mer_number/*k*-mer_depth, where the *k*-mer_number refers to the total number of *k*-mers, and *k*-mer_depth is the depth of the main peak in the k-mer frequency distribution. Therefore, we estimated the genome size of *Z. bungeanum* to be 180,885,352,531/44 ≈ 4.11 Gb.

### 1.2.5 PacBio sequencing

An improved CTAB method was used to extract the genomic DNA. The modified CTAB extraction buffer included 0.1 M Tris-HCl, 0.02 M EDTA, 1.4 M NaCl, 3% (w/v) CTAB, and 5% (w/v) PVP K40. Beta-mercaptoethanol was added to the CTAB extraction buffer to ensure DNA integrity and quality. Genomic DNA was sheared using a g-TUBE device (Covaris Inc., Woburn, MO, USA), into 20 kbp fragments. The sheared DNA was purified and concentrated using Agencourt Ampure XP beads (Beckman Coulter Inc., Pasadena, CA, USA) and further used for single-molecule real-time (SMRT) bell preparation according to the manufacturer’s protocol (Pacific Biosciences, Menlo Park, CA, USA; 20 kb template preparation kit) using BluePippin size selection protocol (Sagescience, Beverly, MA, USA). After size selection, the isolated SMRT bell fractions were purified using Ampure XP beads, after which they were used for primer (V3) and polymerase (2.0) binding according to the manufacturer’s binding calculator (Pacific Biosciences). Single-molecule sequencing was performed on a PacBio Sequel system and only the subreads equal to or longer than 500 bp were used for subsequent genome assembly.

### 1.2.6 Illumina sequencing

We constructed seven libraries with 270 bp insert fragment for *Z. bungeanum* following the Illumina’s protocol (Illumina, San Diego, CA, USA), as follows: (i) genomic DNA was fragmented using a focused-ultrasonicator (Covaris, Woburn, MA, USA); (ii) the DNA ends were polished, and an adenine was added to the ends of the fragments; (iii) DNA adaptors (Illumina) with a single “T” overhang at the 3ʹ end were ligated to the DNA fragments; (iv) the ligation products were run on 2% agarose gels, and the bands corresponding to each insert size were excised; and (v) the insert size was measured using Agilent 2100, and the concentration of the library was quantified using the Q-PCR method. Finally, these libraries were sequenced on a HiSeq 2500 system with a PE150 strategy, following the manufacturer’s instructions (Illumina). The sequencing adaptors and contaminated reads (mitochondrial, bacterial, viral sequences, etc.) of the raw Illumina reads were removed by alignment to the NCBI-NR database using BWA v0.7.13^8^ with default parameters. FastUniq v1.1^9^ was used to remove the duplicated read pairs, and low-quality reads were filtered satisfying the following conditions: 1) reads with ≥10% unidentified nucleotides (N), 2) reads with >10 nucleotides aligned to the adapter, allowing ≤10% mismatches, and 3) reads with >50% bases having Phred quality <5. Finally, we generated a total of 213.81 Gb clean Illumina reads for *Z. bungeanum*.

### 1.2.7 Hi-C sequencing

According to the Hi-C procedure, nuclear DNA from the leaves of *Z. bungeanum* was cross-linked and then cut with the restriction enzyme *Dpn* II, leaving pairs of distally located but physically interacting DNA molecules attached to one another. The sticky ends of these digested fragments were biotinylated and then ligated to each other to form chimeric circles. Biotinylated circles, which are chimeras of the physically associated DNA molecules from the original cross-linking, were enriched, sheared, and sequenced using the Illumina HiSeq X Ten platform with 150 bp paired-end reads. As a result, we obtained a total of 486.7 Gb clean Illumina reads.

### 1.2.8 Genome assembly

The full PacBio long reads were converted to fasta format. First, we used the NextDenovo (v2.3) ([https://github.com/Nextomics/Next](https://github.com/Nextomics/Next Denovo)Denovo) to generate a draft genome assembly with default parameters for Pacbio reads only. We then used NextPolish (v2.0)^10^ to polish the draft genome with both long and short reads to obtain the corrected genome. This was followed by processing using purge_dups to purge the haplotigs and error fragments. This process yielded a total of 16,879 contigs, with contig N50 of 410.1 Kb and total length of ~4.23 Gb. Contigs were clustered with hierarchical clustering of the Hi-C data. To anchor scaffolds onto chromosomes, the Hi-C sequencing data were aligned to the assembly by BWA (aln mode) using the default parameters^8^, and valid contacts were detected. In total, 224,908,615 valid interaction read pairs were used for Hi-C scaffolding. Based on the valid Hi-C interaction read pairs, 16,615 contigs were clustered into 68 pseudochromosomes using ALLHiC^11,12^, of which 16,611 contigs with a total length of 4,124,904,629 bp were ordered and oriented within each group. The percentage gap in the final assembly was only 0.04%.

### 1.2.9 Genome quality assessment

The completeness of the assembly was checked by mapping 2,270 benchmarking universal single-copy orthologs (BUSCOs) and 458 core eukaryotic genes (CEGs) to the genomes using BUSCO v3.0.2b^13^ and CEGMA v2.5^14^, respectively, which showed that the majority of the *Z. bungeanum* genome was successfully reconstructed, as more than 97% BUSCOs and 97% CEGs could be detected. Additionally, we used the LTR assembly index (LAI)^15^ to evaluate the completeness of the assembly. The LAI index refers to the amount of assembled intact-LTR, which can be used to evaluate genomic regions with highly repetitive sequences.

### 1.2.10 Repeated sequence prediction

The repeat components in *Z. bungeanum* assembly were first estimated by building a *de novo* repeat library employing the programs LTR-FINDER^16^, MITE-Hunter^17^, RepeatScout v1.0.5^18^, and PILER-DF^19^, and the output results were merged together and classified using PASTEClassifier v1.0^20^. This *de novo* constructed database together with the Repbase database v20.01^21^ were used to create the final repeat library. Repeat sequences in *Z. bungeanum* were identified and classified using the RepeatMasker program v4.0.6^22^. The LTR family classification criterion was defined by which 5′ LTR sequences of the same family would share at least 80% identity over at least 80% of their length. The expansion history of transposons was estimated by computing the divergence of the transposon *Copia* from the corresponding consensus sequence in the repeat library according to the RepeatMasker output and then calculating the percentage of transposons at different divergence levels.

### 1.2.11 LTR-RTs analysis

Long terminal repeat retrotransposons (LTR-RTs) were identified using the LTR_retriever. We identified a total of 53,470 intact LTR-RTs (the output file with the name “.pass.list”). Then, we extracted the internal regions of all intact LTR-RTs and conducted blastx searches into the non-redundant LTR-RTs library (.LTRlib.fa). By analyzing the best hits from all intact LTR-RTs to the non-redundant LTR-RTs library, the internal regions of all intact LTR-RTs can map up to 3300 LTR-RTs in the non-redundant LTR-RTs library. Therefore, all 53,470 intact LTR-RTs were classified into 3,300 LTR-RT classes, and the number of LTR-RTs in each class was counted.

### 1.2.12 Protein-coding gene prediction

We used *de novo* protein homology and RNA-Seq approaches for protein-coding gene prediction. In detail, Genscan v1.0^23^, Augustus v2.5.5^24^, GlimmerHMM v3.0.1^25^, GeneID v1.3, and SNAP^26^ were used to perform *de novo* gene prediction; the alignment of the homologous peptides from *Arabidopsis thaliana* (The Arabidopsis Information Resource), *Oryza sativa* (Phytozome v12.1), and *Citrus reticulata* (http://citrus.hzau.edu.cn/orange/index.php) to our assemblies were used to identify homologous genes with GeMoMa v1.4.2et^27^; the RNA-Seq reads were assembled into contigs, and *de novo* assembly yielded unigenes, using Trinity, and the resulting unigenes were aligned to the repeat-masked assemblies using BLAT^28^. Subsequently, the gene structures of BLAT alignment results were modeled using PASA^29^, and the protein-coding regions were identified using TransDecoder v3.0.1 (https://github.com/TransDecoder/TransDecoder/) and GeneMarkS-T^30^, respectively. Finally, consensus gene models were generated by integrating *de novo* predictions, protein alignments, and transcript data using EVidenceModeler^31^. In total, 74,307 protein-coding genes were identified. Annotation of the predicted genes was performed by BLAST searches against a series of nucleotide and protein sequence databases, including KOG^32^, KEGG^33^, NCBI-NR, and TrEMBL^34^ with an *E*-value cutoff of 1e-5. Gene ontology (GO) for each gene was assigned by Blast2GO^35^ against the NCBI database.

### 1.2.13 Non-coding RNAs prediction

Non-coding RNAs play important roles in a variety of processes, such as in the genes encoding ribosomal RNAs (rRNAs), transfer RNAs (tRNAs), and microRNAs (miRNAs). The rRNA fragments were identified by aligning the rRNA template sequences against Pfam database v32.0^36^ using BLAST with an *E-value* of 1e-10 and identity cutoff of 95% or more. The tRNAScan-SE algorithms^37^ with default parameters were applied to predict tRNA genes. The miRNA genes were predicted using INFERNAL v1.1^38^ against the Rfam database v14.0^39^ with a cutoff score of 30 or more. The minimum cutoff score was based on the settings that yielded a false-positive rate of 30 bits.

### 1.2.14 Comparative genomics analyses

Protein sequences of *Z. bungeanum*, *Citrus sinensis*, *Arabidopsis thaliana*, *Amborella trichopoda*, *Piper nigrum*, *Zea mays*, *Oryza sativa*, *Papaver somniferum*, *Vitis vinifera*, *Dimocarpus longan*, *Brassica napus*, *Gossypium hirsutum*, *Arachis hypogaea*, *Cucumis sativus*, *Sesamum indicum*, *Capsicum annuum*, and *Nicotiana tabacum* were used for all BLASTP analyses. The results were analyzed using OrthoMCL software^40^ with an MCL inflation of 1.5 to identify gene family clusters. Single-copy gene clusters shared by all ten species were used to construct a phylogenetic tree using PhyML v3.0^41^. The divergence time was estimated using the MCMCtree implemented in PAML package v4.9^42^. Calibration times were obtained from the TimeTree database (<http://www.timetree.org/>). Homologous blocks were detected using Mcscan v1.1^43^. The *K_s_* values of the blocks were calculated using the HKY model^44^. According to the the divergence time between *Z. bungeanum* and *C. sinensis* derived from the phylogenetic tree (Figure 2B, 35.3 MYA), the synonymous substitution rate is 3.92×10^-9^ synonymous substitutions yr^-1^ (T = *Ks*/2λ and λ = 0.277/2×35.3 = 3.92E-9). The *Zanthoxylum*-specific WGD event date was obtained base on the synonymous (*K_s_*) substitutions calculation with λ=3.92E-9.

Expansion and contraction of OrthoMCL-derived gene clusters were determined using CAFÉ v2.1 and were based on changes in gene family size in the inferred phylogenetic history. KEGG and GO annotations of the gene family were completed by aligning the genes to the KEGG database and NCBI non-redundant database using BlastP with an *E* value of 1e−5, respectively. BLAST2GO was used to obtain the associated GO terms. The enrichment score was defined as a hypergeometric test value.

### 1.2.15 Synteny analysis

The genome synteny between and within species was analyzed via all-against-all BLASTP searches of protein sequences (with an *E*-value cut-off of 1e-5). Collinear blocks containing at least 10 genes (-s 10) and a maximum of 25 gaps (genes) between two proximal orthologs within a block (-m 25) were identified using Mcscan v1.1^43^. Synteny was searched for by comparing the *Z. bungeanum* genome with the genomes of *C. sinensis* and *V. vinifera*.

### 1.2.16 Karyotype evolution analysis of Rutaceae

We performed collinearity analysis for each two of the species within *Z.* *bungeanum*, *Xanthoceras sorbifolia*^45^, *C. sinensis*^46^ and *Arabidopsis*^47^ and *Vitis vinifera*^48^ using MCScanX^43^, and the syntenic blocks were identified based on all-versus-all Last alignments included in the JCVI package^49^ with default parameters. The distribution of seven eudicots ancestral chromosomal lineage in each chromosomes of the species were depicted by the syntenic blocks between the ancestral chromosomes represented by the blocks of grape^48^ as described in Bolot *et al*.^50^ and Murat *et al*.^51^ and detected species. Speciation event dates were obtained base on the synonymous (*K_s_*) substitutions calculation (divergence time= *K_s_* / 2 × r) with r=6.5E-9^52^.”

### 1.2.17 Transcriptome analyses

Frozen tissues obtained from pericarps at seven developmental stages were ground with a mortar and pestle. Total RNA was isolated using TRIzol reagent (Invitrogen, Carlsbad, CA, USA), followed by treatment with RNase-free DNase I (Promega, Madison, WI, USA) according to the manufacturers’ protocols. RNA quality was assessed using an Agilent 2100 Bioanalyzer. Illumina RNA-Seq libraries were prepared for 21 samples and sequenced on a HiSeq 2500 system with a PE150 strategy following the manufacturer’s instructions (Illumina). Three biological replicates were analyzed for each developmental stage.

Based on their quality scores, the clean reads were trimmed using the quality trimming program Btrim^53^, and aligned to the *Z. bungeanum* reference assembly using TopHat v2.21^54^. Cufflinks v2.2.1^54^ was used to assemble the mapped reads for each sample. We used the fragments per kilobase of exon model per million mapped fragments (FPKM) as the normalized gene expression level.

### 1.2.18 Co-expression analysis

We constructed a co-expression network using the cluster function in MATLAB. First, 2,752 metabolic genes (average FPKM > 5) were selected based on the KEGG annotation. The standard of FPKM > 5 was selected because the expression profile of low-expression genes is susceptible to sequencing errors. Then, based on the Spearman correlation between genes, the 2,752 metabolic genes were clustered into five subnetwork modules (Figure 4C and Figure S17) as follows: 1) the expression fluctuates throughout pericarp development; 2) the expression decreases sharply at the beginning of pericarp development, then gradually increases at later periods; 3) the expression increases throughout pericarp development; 4) the expression increases during the former periods, but decreases at later periods; and 5) the expression decreases throughout pericarp development. Among them, modules 3, 4, and 5 explained approximately 90% of metabolic genes, thereby revealing the principal expression pattern in pericarp development. KEGG enrichment analysis was conducted for each module to understand the relationship between the enriched pathways and gene expression patterns. The gene number of a metabolic pathway in a module was compared to that of the 2,752 metabolic genes. The *p*-values were calculated by a hypergeometric test and adjusted using the Benjamini–Hochberg procedure.

### 1.2.19 Comparison of transcriptomes between *Z. bungeanum* and *C. sinensis*

Gene expression in *C. sinensis* was referenced from a previous study^55^ in which transcriptome sequencing of the pericarps were performed at 90, 120, 150, 180, and 210 days after full bloom. To determine the gene expression between *Z. bungeanum* and *C. sinensis* pericarps, we first identified 14,675 orthologous genes between the two species. Then, according to the equal medians of gene expression in the 14,675 orthologs of the two species, the gene expression of *C. sinensis* was normalized by dividing by 4.78.

### 1.2.20 Analysis of gene family expansion

The protein families that expanded in *Z. bungeanum* compared to *C. sinensis* were considered to be the gene expansion of *Zanthoxylum* (*p* < 0.05). The KEGG annotations of *C. sinensis* and *A. thaliana* were downloaded from the KEGG website (<https://www.genome.jp/kegg/>). KEGG annotation of *Z. bungeanum* was performed using the KEGG Automatic Annotation Server (KAAS) platform.

The Acyl-ACP thioesterases and acetyltransferases in *Z. bungeanum*, *C. sinensis*, and *A. thaliana* were predicted using hmmsearch in conjunction with the acetyltransferase and thioesterase family hmm models PF01643 and PF02458 (*E*-value < 1e-6) from Pfam^36,56^. Then, we tested whether the gene number of the two gene families in one plant was significantly higher than that in another plant by comparing the background gene number between the two plants. The *p*-values were calculated by a hypergeometric test and adjusted using the Benjamini–Hochberg procedure.

### 1.2.21 Annotation and analysis of terpene synthases

The TPSs in the *Z. bungeanum* genome were predicted using hmmsearch in conjunction with the terpene synthase family hmm model PF03936 (*E*-value < 1e-6) from Pfam^36,56^. A total of 70 TPS genes were predicted in the *Z. bungeanum* genome (Table S20). Similarly, we annotated 55 and 33 TPS genes in *C. sinensis* and *A. thaliana*, respectively (Table S22).

To analyze the evolution of the TPS gene family in *Z. bungeanum*, *C. sinensis*, and *A. thaliana*, the 158 (70 + 55 + 33) TPS proteins were further classified into 10 TPS families and 41 TPS subfamilies (Table S22) based on three criteria: (1) the proteins in a family or subfamily had relatively closer phylogenetic relationships in the phylogenetic tree constructed by the alignment of all TPS proteins; (2) the identity between two protein sequences in a family was higher than 45%; and (3) the identity between two protein sequences in a subfamily was higher than 60%. Among them, the 70 TPS genes in *Z. bungeanum* were classified into 8 TPS families and 18 TPS subfamilies.

### 1.2.22 Extraction, Isolation, and purification of compounds

The pericarps of *Z. bungeanum* were shattered by a universal highspeed smashing machine (FW-200, Beijing Zhongxing Weiye Instrument Co., Ltd., China) and homogenized by 40 and 60-mesh screen. The dried ground powder (2.0 kg) was soaked overnight thrice in 95% ethanol (2.5 L each) with 2 weeks, ultrasonication and water-bath (45℃) for 3 to 4 hours per day. Subsequently, the combined extract was digested in methanol for a week. The combined filtrates were concentrated in *vacuo*. The structure of purified compounds was identified by NMR and mass spectrum. The separation procedure of the crude extract was as follows:


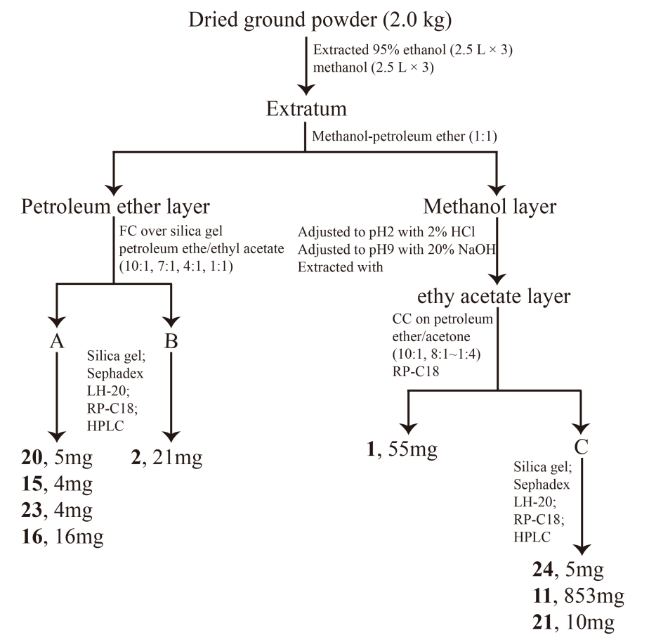


### 1.2.23 Extraction and HPLC analysis of hydroxyl-β-sanshool

Hydroxyl-β-sanshool was extracted from seven pericarp developmental stages of the *Z. bungeanum* cultivar ‘DaHongPao’ and used for transcriptome sequencing according to the method described by Ke *et al*.^57^. In brief, 0.2 g of freeze-dried tissue was ground in liquid nitrogen and extracted with 10 mL methanol using an ultrasonic cleaner for 20 min at 30 °C. The mixtures were shaken well and centrifuged at 10,000 rpm for 5 min. Subsequently, the supernatants were filtered through a 0.22 μm membrane filter before HPLC analysis. The hydroxyl-β-sanshool content in the extracts was measured using an Agilent 1260 series HPLC-DAD system (Agilent, USA). A Zorbax SB-C18 column (4.6 mm × 250 mm, 5 μm) was used, and the samples were eluted at 35 °C at a flow rate of 0.5 mL/min. The detection wavelength was 278 nm. The filtered sample (2 μL) was injected into the HPLC system for analysis. The mobile phase consisted of acetonitrile (A) and deionized water (B), and the gradient elution was as follows: 0 min, 35% A; 5–10 min, 35%–40% A; 25–50 min, 45%–65% A; 55–60 min, 90%–35% A.

# 1.3 Identification of reported alkamide compounds

**Compound 1**: C_16_H_25_NO_2_E; SI-MS (positive) *m/z*: 286.242[M+Na]^+^；^13^C NMR (126 MHz, MeOD) δ 168.0(C-1), 144.6(C-3), 132.4(C-6), 132.0(C-7), 131.8(C-10), 131.7(C-9), 130.4(C-8), 129.6(C-11), 123.9(C-2), 71.0(C-2'), 50.4(C-1'), 32.2(C-4), 31.7(C-6), 26.9(C-3',4'), 18.3(C-12)；^1^H NMR (500 MHz, MeOD) δ 6.79 (dt, J = 15.4, 6.6 Hz, 1H, H-3), 6.14 – 5.98 (m, 5H, H-2,7,8,9,10), 5.66 (tt, J = 13.4, 6.7 Hz, 2H, H-6,11), 3.25 (s, 2H, H-1'), 2.29 (ddd, J = 16.0, 10.2, 4.1 Hz, 4H, H-4,5), 1.74 (d, J = 7.3 Hz, 3H, H-12), 1.17 (s, 6H, H-3',4').

**Compound 3**: ESI-MS（positive）*m/z*: 318.48 [M+Na]+；1H NMR (500 MHz, MeOD) δ 7.26 (dd, J = 15.6, 10.7 Hz, 1H, H-8), 6.80 – 6.73 (m, 1H, H-3), 6.42 – 6.35 (m, 1H, H-10), 6.26 (dd, J = 15.2, 5.3 Hz, 1H, H-9), 6.20 (d, J = 15.6 Hz, 1H, H-7, H-7), 6.01 (d, J = 15.4 Hz, 1H, H-2), 4.35 (p, J = 6.2 Hz, 1H, H-11), 3.23 (d, J = 4.5 Hz, 2H, H-2'), 2.79 (t, J = 7.2 Hz, 2H, H-5), 2.50 – 2.43 (m, 2H, H-4), 1.24 (dd, J = 8.1, 4.0 Hz, 3H, H-12), 1.15 (s, 6H, H-3',4')；13C NMR (126 MHz, MeOD) δ 201.6(C-6), 168.9(C-1), 149.5(C-9), 144.5(C-3), 144.5(C-8), 130.2(C-7), 128.0(C-10), 125.2(C-2), 71.6(C-1'), 68.4(C-11), 51.1(C-2'), 39.4(C-5), 27.4(C-4), 27.3(C-3',4'), 23.2(C-12).

**Compound 4**: ESI-MS（positive）*m/z*: 318.38 [M+Na]^+^；^1^H NMR (500 MHz, MeOD) δ 7.28 (dd, J = 15.6, 10.8 Hz, 1H, H-9), 6.81 (dt, J = 15.2, 7.0 Hz, 1H, H-3), 6.45 (dd, J = 15.7, 10.8 Hz, 1H, H-8), 6.28 (dd, J = 15.3, 5.7 Hz, 1H, H-7), 6.16 (d, J = 15.7 Hz, 1H, H-10), 6.03 (dt, J = 15.3, 1.4 Hz, 1H, H-2), 4.23 (q, J = 6.0 Hz, 1H, H-6), 3.25 (s, 2H, H-1'), 2.35 – 2.29 (m, 2H, H-4), 2.28 (s, 3H, H-12), 1.70 (dt, J = 14.7, 5.9 Hz, 2H, H-5), 1.17 (s, 6H, H-3', 4')；13C NMR (126 MHz, MeOD) δ 201.5(C-11), 169.0(C-1), 148.0(C-7), 145.4(C-9), 145.2(C-3), 131.2(C-10), 129.0(C-8), 125.0(C-2), 71.7(C-6), 71.6(C-2'), 51.1(C-1'), 36.5(C-5), 28.9(C-4), 27.2(C-3',4'), 27.1(C-12).

**Compound 5**: ESI-MS（positive）*m/z*: 318.38 [M+Na]^+^；^1^H NMR (500 MHz, MeOD) δ 6.81 (dt, J = 15.3, 6.9 Hz, 1H, H-3), 6.25 – 6.17 (m, 2H, H-8,10), 6.04 – 6.00 (m, 1H, H-2), 5.77 – 5.62 (m, 2H, H-7,9), 4.27 (p, J = 6.3 Hz, 1H, H-11), 4.10 (q, J = 6.5 Hz, 1H, H-6), 3.25 (s, 2H, H-1'), 2.28 (qt, J = 15.1, 7.5 Hz, 2H, H-2), 1.72 – 1.60 (m, 2H, H-5), 1.23 (d, J = 6.4 Hz, 3H, H-12), 1.18 (s, 6H, H-3',4')；13C NMR (126 MHz, MeOD) δ 169.1(C-1), 145.5(C-3), 138.7(C-10), 137.0(C-7), 131.2(C-8), 129.8(C-9), 124.8(C-2), 72.3(C-6), 71.6(C-2'), 68.8(C-12), 51.1(C-1'), 36.9(C-5), 29.1(C-4), 27.2(C-3',4'), 23.6(C-13).

**Compound 6**: ESI-MS（positive）*m/z*: 316.37 [M+Na]^+^；^1^H NMR (500 MHz, MeOD) *δ* 7.37 – 7.27 (m, 2H, H-8,9), 6.79 (dt, *J* = 15.3, 6.8 Hz, 1H, H-3), 6.65 – 6.49 (m, 1H, H-7), 6.03 (dt, *J* = 15.3, 1.5 Hz, 1H, H-2), 3.24 (s, 2H, H-1'), 2.87 (t, *J* = 7.1 Hz, 2H, H-5), 2.51 (qd, *J* = 7.0, 1.3 Hz, 2H, H-4), 2.33 (s, 3H, H-12), 1.17 (s, 4H, H-3',4')；^13^C NMR (126 MHz, MeOD) *δ* 200.7(C-6), 200.5(C-11), 168.9(C-1), 144.2(C-3), 141.9(C-8), 141.0(C-10), 138.0(C-0), 137.2(C-7), 125.4(C-2), 71.6(C-2'), 51.0(C-1'), 39.9(C-5), 27.5(C-12), 27.2(C-3',4'), 27.1(C-4).

**Compound 7**: ESI-MS（positive）*m/z*: 320.38 [M+Na]^+^；^1^H NMR (500 MHz, MeOD) *δ* 6.79 (dt, *J* = 15.3, 6.8 Hz, 1H, H-3), 6.60 (dd, *J* = 15.05Hz, 1H, H-8), 6.05(dd, *J*=20.0, 13.2Hz, 1H, H-2,7), 5.68 (1H, dd, *J* = 15.05Hz, H-9), 5.43 (dt, *J* = 10.7, 7.5Hz, 1H, H-6),3.92 (t,*J* = 6.4Hz, 1H, H-10), 3.61(p*, J*=6.4Hz,1H, H-11), 3.25(s, 2H, H-1'), 2.39 (dd, *J*=14.4, 6.9Hz, 2H, H-5), 2.28 (dd*, J=*14.6, 7.4Hz, 2H, H-4), 1.17(s, 6H, H-3',4'), 1.11 (d, *J* = 6.4 Hz, 3H, H-12)；^13^C NMR (126MHz, MeOD) *δ* 169.1(C-1), 145.0(C-3), 134.2(C-9), 131.5(C-6), 130.2(C-8), 128.1(C-7), 125.1(C-2), 77.8(C-10), 71.7(C-11), 71.6(C-2'), 51.1(C-1'), 33.1(C-5), 27.5(C-4), 27.1(C-3',4'), 18.9(C-12).

**Compound 8**: ESI-MS（positive）*m/z*: 320.38 [M+Na]^+^；^1^H NMR (500 MHz；MeOD) *δ* 6.80 (1H, dt, *J* = 15.3,6.8Hz, H-3), 6.05(dd, *J*=24.5, 13.3Hz, 1H, H-2,7), 5.76 (dd, *J* = 15.2, 6.6Hz, 1H, H-8), 5.68 (d, *J* = 15.05Hz, 1H, H-9), 5.43 (dt, *J* = 18.2, 7.5Hz, 1H, H-6), 3.96 (t, *J* = 5.7Hz, 1H, H-10), 3.61(p, *J* = 6.4Hz, 1H, H-11), 3.25(s, 2H, H-1'), 2.39 (dd, *J =* 14.4, 7.4Hz, 2H, H-5), 2.30 (dd, *J*=14.6, 6.9Hz, 2H, H-4), 1.14 (d, *J* = 6.4 Hz, 3H, H-12), 1.17(s, 6H, H-3',4')；^13^C NMR (126 MHz，MeOD) *δ* 169.0(C-1), 125.1(C-2), 145.0(C-3), 134.4(C-9), 131.3(C-6), 130.1(C-7), 127.9(C-8), 77.5(C-10), 71.6(C-2'), 71.6(C-11), 51.1(C-1'), 33.1(C-5), 27.5(C-4), 27.2(C-3',4'), 18.7(C-12).

**Compound 10**: ESI-MS（positive）*m/z*: 334.40 [M+Na]^+^；^1^H NMR (500 MHz, MeOD) *δ* 6.87 – 6.80 (m, 1H, H-3), 6.30 – 6.22 (m, 2H, H-7,9), 6.04 (d, *J* = 15.4 Hz, 1H, H-2), 5.74 (dd, *J* = 14.0, 6.8 Hz, 1H, H-8), 5.57 (dd, *J* = 13.7, 7.5 Hz, 1H, H-10), 4.13 (q, *J* = 6.7 Hz, 1H, H-6), 3.85 – 3.79 (m, 1H, H-11), 3.28 – 3.26 (m, 4H, H-13,2'), 2.31 (ddd, *J* = 13.9, 8.3, 6.9 Hz, 2H, H-4), 1.24 (d, *J* = 6.4 Hz, 3H, H-12), 1.20 (s, 6H, H-3',4')；^13^C NMR (126 MHz, MeOD) *δ* 169.2(C-1), 145.4(C-3), 137.7(C-8), 135.8(C-10), 132.6(C-9), 130.7(C-7), 124.9(C-2), 79.0(C-11), 72.2(C-6), 71.6(C-2'), 56.2(C-13), 51.1(C-1'), 36.9(C-5), 29.0(C-4), 27.2(C-3',4'), 21.5(C-12)

**Compound 16**: C_18_H_33_NO_2_; ESI-MS（positive）*m/z*: 318.25 [M+Na]^+^；^13^C NMR (126 MHz, CDCl_3_)*δ*167.7(C-1), 143.9(C-5), 142.1(C-3), 128.3(C-4), 121.3(C-2), 71.1(C-2'), 50.7(C-1'), 33.1(C-6), 32.0(C-12), 29.7(C-8), 29.6(C-9), 29.4(C-10), 29.3(C-11), 28.9(C-7),27.4(C-3',4'), 22.8(C-13), 14.2(C-14)。^1^H NMR (500 MHz, CDCl_3_) *δ* 7.19 (dd, *J* = 14.9, 10.1 Hz, 1H, H-3), 6.15 – 6.02 (m, 2H, H-4,5), 5.81 (d, *J* = 15.0 Hz, 1H, H-2), 3.33 (d, *J* = 6.0 Hz, 2H, H-1'), 2.13 (dd, *J* = 13.9, 6.9 Hz, 2H, H-6), 1.38 (d, *J* = 6.8 Hz, 3H, H-11), 1.24 (d, *J* = 8.9 Hz, 12H, H-7,8,10,11,12,13), 1.22 (s, 6H, H-3',4'), 0.87 (t, *J* = 6.9 Hz, 3H, H-14).

**Compound 20**: C_18_H_29_NO_2_; ^1^H NMR (500 MHz, MeOD) *δ* 7.13 (dd, *J* = 15.1, 10.7 Hz, 1H, H-3), 6.23 (dd, *J* = 15.0, 10.8 Hz, 1H, H-4), 6.14 – 6.07 (m, 1H, H-5), 6.00 (d, *J* = 15.1 Hz, 1H, H-2), 5.41 – 5.33 (m, 3H, H-8,9,11), 5.32 – 5.24 (m, 1H, H-12), 3.26 (d, *J* = 7.1 Hz, 2H, H-1'), 2.79 (dd, *J* = 13.6, 7.8 Hz, 2H, H-10), 2.27 – 2.18 (m, 4H, H-6,7), 2.13 – 2.03 (m, 2H, H-13), 1.18 (s, 6H, H-3',4'), 1.00 – 0.92 (m, 3H, H-14)；^13^C NMR (126 MHz, MeOD) δ 169.5(C-1), 143.3(C-5), 142.4(C-3), 132.7(C-11), 130.2(C-4), 130.0(C-8), 129.7(C-12), 128.2(C-9), 123.2(C-2), 71.7(C-2'), 51.2(C-1'), 33.9(C-6), 27.6(C-7), 27.2(C-3',4'), 26.4(C-10), 21.6(C-13), 14.6(C-14).

**Compound 21**: C_18_H_27_NO_2_; ESI-MS（positive）*m/z*: 312.40 [M+Na]^+^；^13^C NMR (126 MHz, MeOD) *δ* 169.5(C-1), 143.2(C-5), 142.4(C-3), 134.6(C-11), 133.3(C-12), 130.8(C-8), 130.7(C-9), 130.5(C-13), 130.3(C-4), 126.6(C-10), 123.2(C-2), 71.7(C-2'), 51.2(C-1'), 34.0(C-6), 28.1(C-7), 27.2(C-3',4'), 18.4(C-14)。^1^H NMR (500 MHz, MeOD) *δ* 7.13 (dd, *J* = 15.1, 10.7 Hz, 1H, H-3), 6.38 (dd, *J* = 17.9, 7.1 Hz, 1H, H-10), 6.29 – 6.18 (m, 1H, H-4), 6.18 – 6.07 (m, 3H, H-5,11,12), 6.01 (t, *J* = 11.9 Hz, 2H, H-2,9), 5.71 (dt, *J* = 20.7, 6.7 Hz, 1H, H-13), 5.37 (dd, *J* = 18.2, 7.5 Hz, 1H, H-8), 3.26 (s, 2H, H-1'), 2.38 – 2.30 (m, 2H, H-7), 2.27 (dd, *J* = 13.5, 6.6 Hz, 2H, H-6), 1.76 (d, *J* = 6.9 Hz, 3H, H-14), 1.16 (d, *J* = 22.7 Hz, 6H, H-3',4').

## References

1. Chinese Pharmacopoeia Commission. Chinese Pharmacopoeia (in Chinese) Shanghai: Science and Technology Press of Shanghai. pp, 275 (1977).
2. Chinese Pharmacopoeia Commission. Chinese Pharmacopoeia (in Chinese) Shanghai: Science and Technology Press of Shanghai. pp, 149 (2010).
3. Chinese Pharmacopoeia Commission. Chinese Pharmacopoeia (in Chinese) Shanghai: Science and Technology Press of Shanghai. pp, 159-160 (2015).
4. Zhang, M. et al. *Zanthoxylum bungeanum* Maxim. (Rutaceae): A systematic review of its traditional uses, botany, phytochemistry, pharmacology, pharmacokinetics, and toxicology. *Int. J. Mol. Sci.* **18**: 2172 (2017).
5. Zhao, G. et al. The *Aegilops tauschii* genome reveals multiple impacts of transposons. *Nat. Plants* **3**:946-955 (2017).
6. Luo, X., Liu, J., Wang, J., Gong, W., Chen, L. & Wan, W. FISH analysis of *Zanthoxylum* *armatum* based on oligonucleotides for 5S rDNA and (GAA)_6_. *Genome* **61**:699-702 (2018).
7. Marçais, G. & Kingsford, C. A fast, lock-free approach for efficient parallel counting of occurrences of *k*-mers, *Bioinformatics* **27**:764–770 (2011).
8. Li, H. & Durbin, R. Fast and accurate long-read alignment with Burrows-Wheeler Transform. *Bioinformatics* **26**:589-595 (2009).
9. Xu, H., Luo, X., Qian, J., Pang, X., Song, J., Qian, G., Chen, J. & Chen, S. FastUniq: a fast de novo duplicates removal tool for paired short reads. *Plos one* **7**:e52249 (2012).
10. Hu, J., Fan, J., Sun, Z. & Liu, S. NextPolish: a fast and efficient genome polishing tool for long read assembly. *Bioinformatics* **36**: 2253–2255 (2019).
11. Zhang, J. et al. Allele-defined genome of the autopolyploid sugarcane *Saccharum spontaneum* L. *Nat. Genet.* **50**: 1565–1573 (2018).
12. Zhang, X., Zhang, S., Zhao, Q., Ming, R. & Tang, H. Assembly of allele-aware, chromosomal-scale autopolyploid genomes based on Hi-C data. *Nat. Plants* **5**: 833–845 (2019).
13. Simao, F. A., Waterhouse, R. M., Ioannidis, P., Kriventseva, E. V. & Zdobnov, E. M. BUSCO: assessing genome assembly and annotation completeness with single-copy orthologs. *Bioinformatics* **31**:3210-3212 (2015).
14. Parra, G., Bradnam, K. & Korf, I. CEGMA: a pipeline to accurately annotate core genes in eukaryotic genomes. *Bioinformatics* **23**:1061-1067 (2007).
15. Qu, S., Chen, J. & Jiang, N. Assessing genome assembly quality using the LTR Assembly Index (LAI). *Nucleic Acids Res.* **46**: e126 (2018).
16. Xu, Z. & Wang, H. LTR_FINDER: an efficient tool for the prediction of full-length LTR retrotransposons. *Nucleic Acids Res.* **35**: 265-268 (2007).
17. Han, Y. & Wessler, S. R. MITE-Hunter: a program for discovering miniature inverted-repeat transposable elements from genomic sequences. *Nucleic Acids Res.* **38**: e199 (2010).
18. Han, Y. & Wessler, S. R. MITE-Hunter: a program for discovering miniature inverted-repeat transposable elements from genomic sequences. *Nucleic Acids Res.* **38**: e199 (2010).
19. Edgar, R. C. & Myers, E. W. PILER: identification and classification of genomic repeats. intelligent systems in molecular biology **21**: 152-158 (2005).
20. Wicker, T. et al. A unified classification system for eukaryotic transposable elements. *Nat. Revi. Genet.* **8**: 973-982 (2007).
21. Bao, W., Kojima, K. & Kohany, O. Repbase Update, a database of repetitive elements in eukaryotic genomes. *Mobile DNA* **6**: 11-11 (2015).
22. Chen, N. Using repeatMasker to identify repetitive elements in genomic sequences. *Curr. Protoc. Bioinformatics* **25**: 4.10.11-14.10.14 (2004).
23. Burge, C. & Karlin, S. Prediction of complete gene structures in human genomic DNA. *J. Mol. Biol.* **268**:78-94 (1997).
24. Stanke, M. & Waack, S. Gene prediction with a hidden Markov model and a new intron submodel. *Bioinformatics* **19**:ii215-225 (2003).
25. Majoros, W. H., Pertea, M., Salzberg, S. L. TigrScan and GlimmerHMM: two open source ab initio eukaryotic gene-finders. *Bioinformatics* **20**:2878-2879 (2004).
26. Blanco, E., Parra, G. & Guigó, R. Using geneid to identify genes. *Curr. Protoc. Bioinform.* Chapter 4, 4–3 (2007).
27. Keilwagen, J., Wenk, M., Erickson, J. L., Schattat, M. H., Grau, J. & Hartung, F. Using intron position conservation for homology-based gene prediction, *Nucleic Acids Res.* **44**:e89 (2016).
28. Kent, W. J. BLAT—the BLAST-like alignment tool. *Genome Res*. **12**:656-664 (2002).
29. Haas, B. J. et al. Improving the *Arabidopsis* genome annotation using maximal transcript alignment assemblies. *Nucleic Acids Res.* **31**:5654-5666 (2003).
30. Tang, S., Lomsadze, A. & Borodovsky, M. Identification of protein coding regions in RNA transcripts. *Nucleic Acids Res.* **43**:e78 (2015).
31. Haas, B. J. et al. Automated eukaryotic gene structure annotation using EVidenceModeler and the Program to Assemble Spliced Alignments. *Genome Biol.* **9**: 1-22 (2008).
32. Tatusov, R. L. et al. The COG database: an updated version includes eukaryotes. *BMC Bioinformatics* **4**: 41-41 (2003).
33. Kanehisa, M. & Goto, S. KEGG: Kyoto Encyclopedia of Genes and Genomes. *Nucleic Acids Res.* **28**: 27-30 (1999).
34. Boeckmann, B. et al. The SWISS-PROT protein knowledgebase and its supplement TrEMBL in 2003. *Nucleic Acids Res.* **31**: 365-370 (2003).
35. Conesa, A., Gotz, S., Garciagomez, J. M., Terol, J., Talon, M. & Robles, M. Blast2GO: a universal tool for annotation, visualization and analysis in functional genomics research. *Bioinformatics* **21**: 3674-3676 (2005).
36. Finn, R. D. et al. Pfam: the protein families database. *Nucleic Acids Res.* **42**: 222-230 (2014).
37. Lowe, T. M. & Eddy, S. R. tRNAscan-SE: a program for improved detection of transfer RNA genes in genomic sequence. *Nucleic Acids Res.* **25**: 955-964 (1997).
38. Nawrocki, E. P. & Eddy, S. R. Infernal 1.1: 100-fold faster RNA homology searches. *Bioinformatics* **29**: 2933-2935 (2013).
39. Griffiths-Jones, S., Bateman, A., Marshall, M., Khanna, A. & Eddy, S. R. Rfam: an RNA family database. *Nucleic Acids Res.* **31**: 439-441 (2003).
40. Li, L., Stoeckert, C. J., Roos, D. S. OrthoMCL: identification of ortholog groups for eukaryotic genomes. *Genome Res* **13**: 2178-2189 (2003).
41. Guindon, S., Dufayard, J., Lefort, V., Anisimova, M., Hordijk, W. & Gascuel, O. New algorithms and methods to estimate maximum-likelihood phylogenies: assessing the performance of PhyML 3.0. *Systematic Biol.* **59**: 307-321 (2010).
42. Yang, Z. PAML 4: phylogenetic analysis by maximum likelihood. *Mol. Biol. Evol.* **24**: 1586-1591 (2007).
43. Wang, Y. et al. MCScanX: a toolkit for detection and evolutionary analysis of gene synteny and collinearity. *Nucleic Acids Res.* **40**: e49 (2012).
44. Hasegawa, M., Kishino, H. & Yano, T. Dating of the human-ape splitting by a molecular clock of mitochondrial DNA. *J. Mol. Evol.* **22**:160-174 (1985).
45. Liang, Q. et al. The genome assembly and annotation of yellowhorn (*Xanthoceras sorbifolium* Bunge). *GigaScience* **8**: 1-15 (2019).
46. Xu, Q. et al. The draft genome of sweet orange (*Citrus sinensis*). *Nat. Genet.* **45**: 59-66 (2013).
47. Arabidopsis Genome Initiative. Analysis of the genome sequence of the flowering plant *Arabidopsis thaliana*. *Nature* **408**: 796-815 (2000).
48. Jaillon, O. et al. The grapevine genome sequence suggests ancestral hexaploidization in major angiosperm phyla. *Nature* **449**: 463-467 (2007).
49. Tang, H., Bowers, J. E., Wang, X., Ming, R., Alam, M. & Paterson, A. H. Synteny and collinearity in plant genomes. *Science* **320**:486-488 (2008).
50. Bolot, S. et al. The ‘inner circle’ of the cereal genomes. *Curr. Opin. Plant biol*. 12:119-125 (2009).
51. Murat, F., Armero, A., Pont, C., Klopp, C. & Salse, J. Reconstructing the genome of the most recent common ancestor of flowering plants. *Nat. Genet.* **49**: 490-496 (2017).
52. Gaut, B. S., Morton, B.R., McCaig, B. C. & Clegg, M. T. Substitution rate comparisons between grasses and palms: synonymous rate differences at the nuclear gene *Adh* parallel rate differences at the plastid gene *rbc*L. *P. Natl. Acad. Sci. USA* **93**:10274-10279 (1996).
53. Kong, Y. Btrim: a fast, lightweight adapter and quality trimming program for next-generation sequencing technologies. *Genomics* **98**:152-153 (2011).
54. Pollier, J., Rombauts, S., Goossens, A. Analysis of RNA-Seq data with TopHat and Cufflinks for genome-wide expression analysis of Jasmonate-Treated plants and plant cultures. *Methods Mol. Biol.* **1011**: 305-315 (2013).
55. Huang, H. et al. Global increase in DNA methylation during orange fruit development and ripening. *P. Natl. Acad. Sci. USA.* **116**: 1430-1436 (2019).
56. Punta, M. et al. The Pfam protein families database. *Nucleic Acids Res.* **30**: 276-280 (2000).
57. Ke J, Qu Y, Li S, Shen G, Chen A, Luo Q, Liu X, Wu H, Li M, Pu B, et al. (2018) Application of HPLC fingerprint based on acid amide components in Chinese prickly ash (*Zanthoxylum*). *Ind. Crop Prod.* **119**: 267-276.

**2. Supplemental Figures**


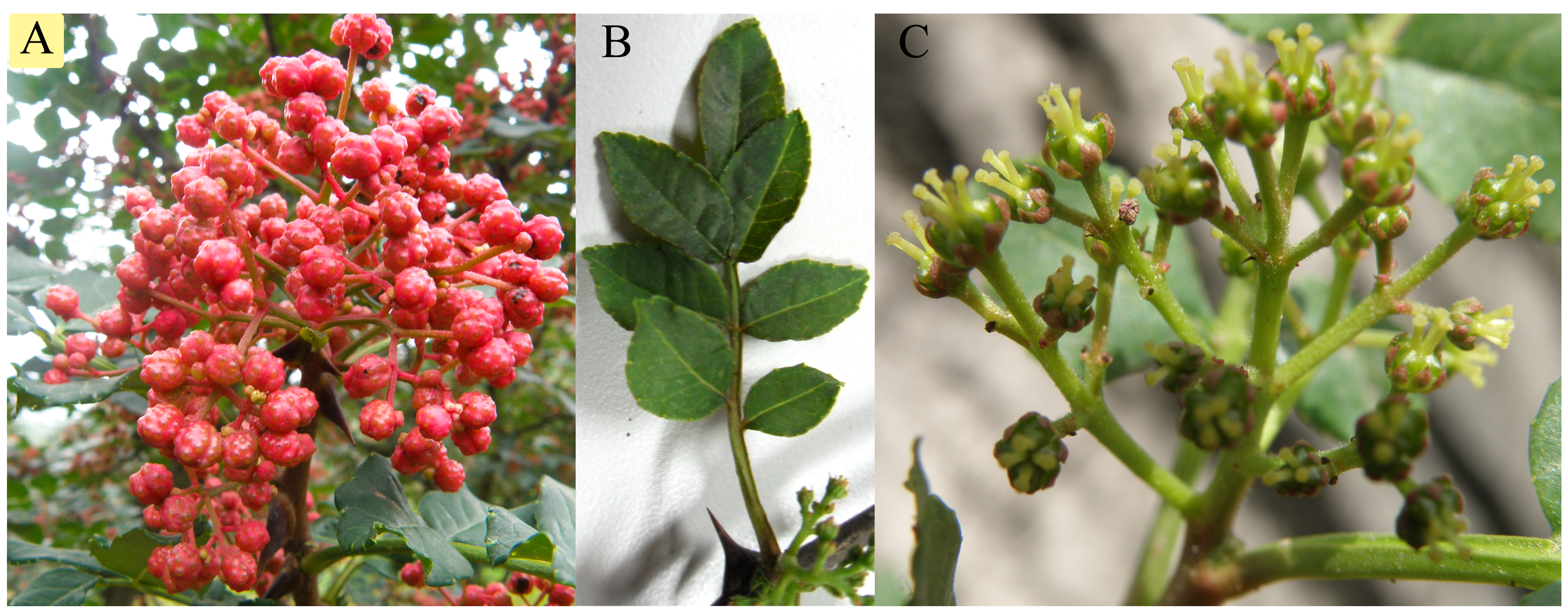


Figure S1 Flower, leaf and fruits of *Z. bungeanum*, which is small deciduous trees or shrubs with short spines on the branches.


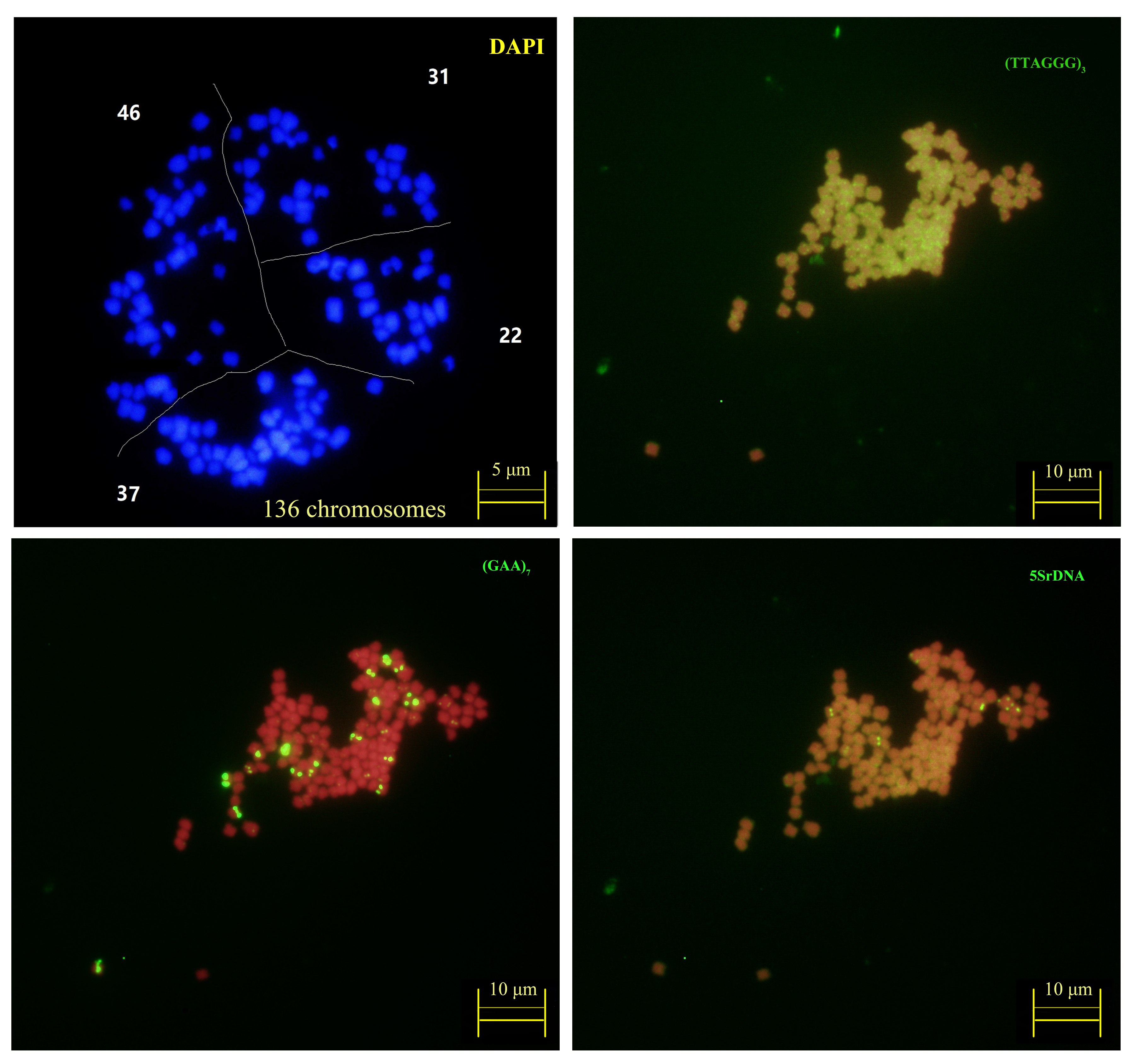


**Figure S2** Karyograms of the *Z. bungeanum* chromosomes. Sequential fluorescent in situ hybridization (FISH) using the probes oligo-nucleotide probes Oligo-Telo (TTAGGG)_3_, Oligo-(GAA)_7_ and Oligo-5SrDNA (GTACTACTCTCGCCCAAGCAC

GCTTAACTTCGGAGTTC). Chromosomes stained with DAPI.


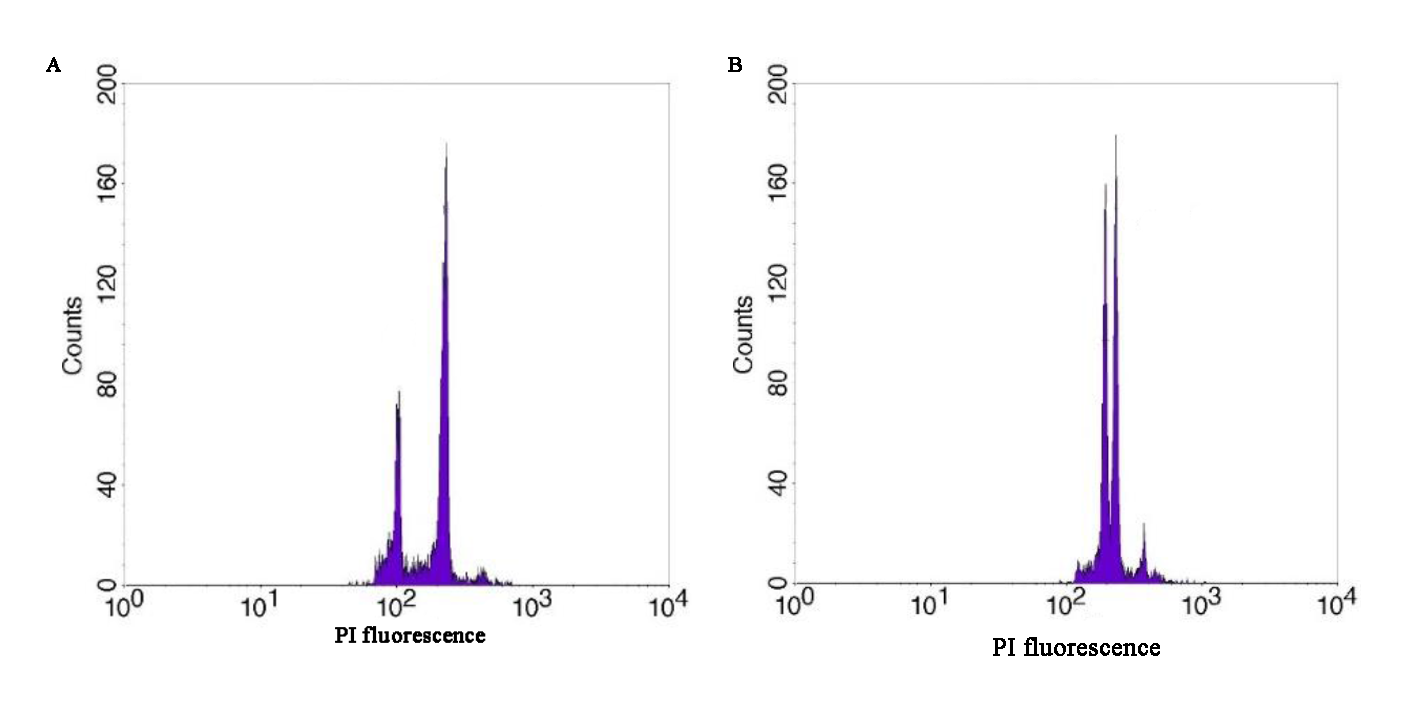


**Figure S3** Estimation of genome size in Chinese peppr by flow cytometry using maize (2.3 Gb / 1C) and pea (4.45 Gb / 1C) as the calibration standard. GenomeSizeunknown = ((G1_PI_fluorunknown)/(G1_PI_fluorstandard)) × GenomeSizestandard, where the GenomeSizeunknown and GenomeSizestandard refer to the genome size of Chinese pepper and reference species (maize and pea in this study), respectively; G1_PI_fluorunknown and G1_PI_fluorstandard refer to the channel number of PI fluorescence of Chinese pepper and reference species (maize and pea in this study) during G1 phase, respectively. (A) Maize as standard: (210.08/115.78) × 2.3 Gb = 4.17 Gb; (B) Pea as standard: (215.27/203.54) × 4.45 Gb = 4.70 Gb, Chinese pepper genome = (4.17 Gb+4.39 Gb)/2= 4.43 Gb. The number of captured cells is more than 5,000, and the vertical axis shows the maximum value of a single row of cells and the X-axis is the fluorescence value.


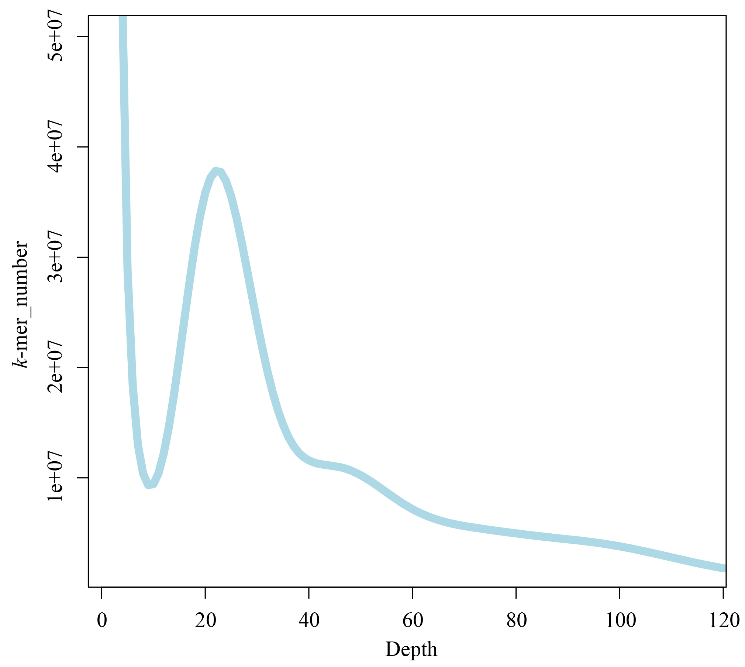


**Figure S4** The *K*-mer distribution of sequencing reads from the *Z. bungeanum* genome. For *k*-mer estimation, following the formula: genome size = k-mer_number/*k*-mer_depth, where the *k*-mer_number refers to the total number of *k*-mers, and *k*-mer_depth is the depth of the main peak in the *k*-mer frequency distribution. The estimated genome size was 180,885,352,531/44 ≈ 4.11 Gb.


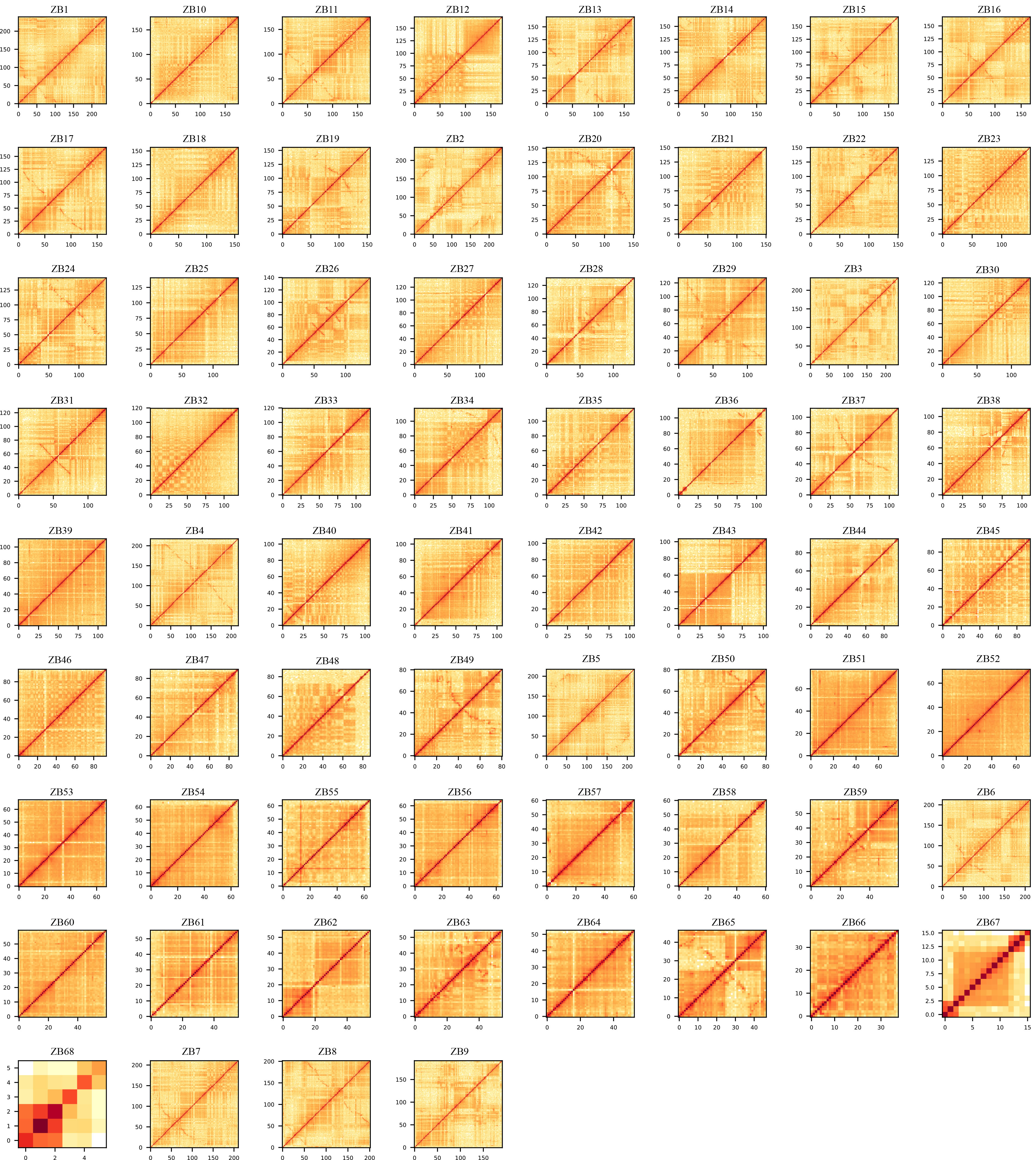


Figure S5 Heatmap showing Hi-C interactions within each supscaffolds.


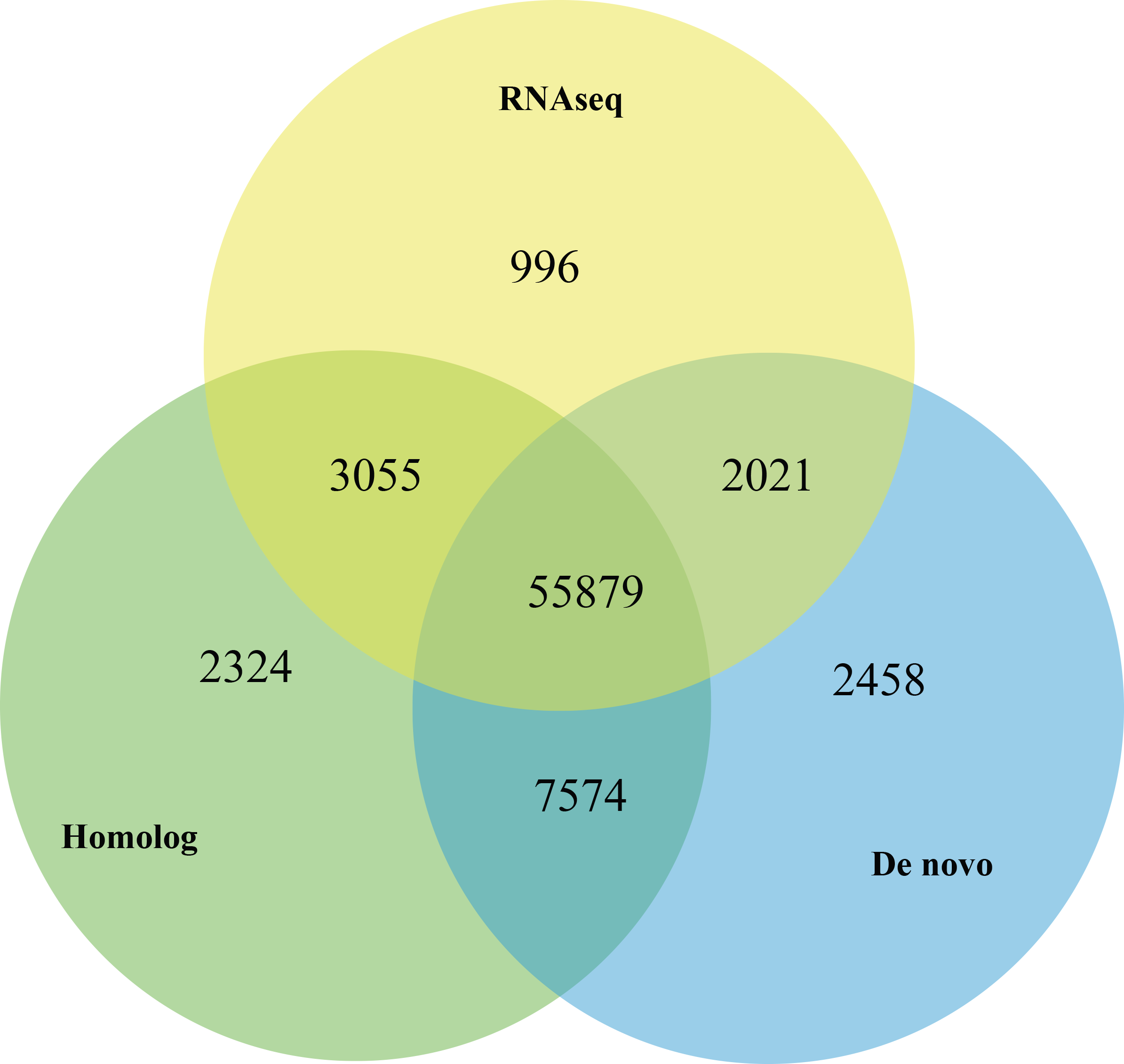


Figure S6 Venn diagram showing number of genes identified by *de novo* assembly, RNA-seq, and by orthology with existing protein sequences.


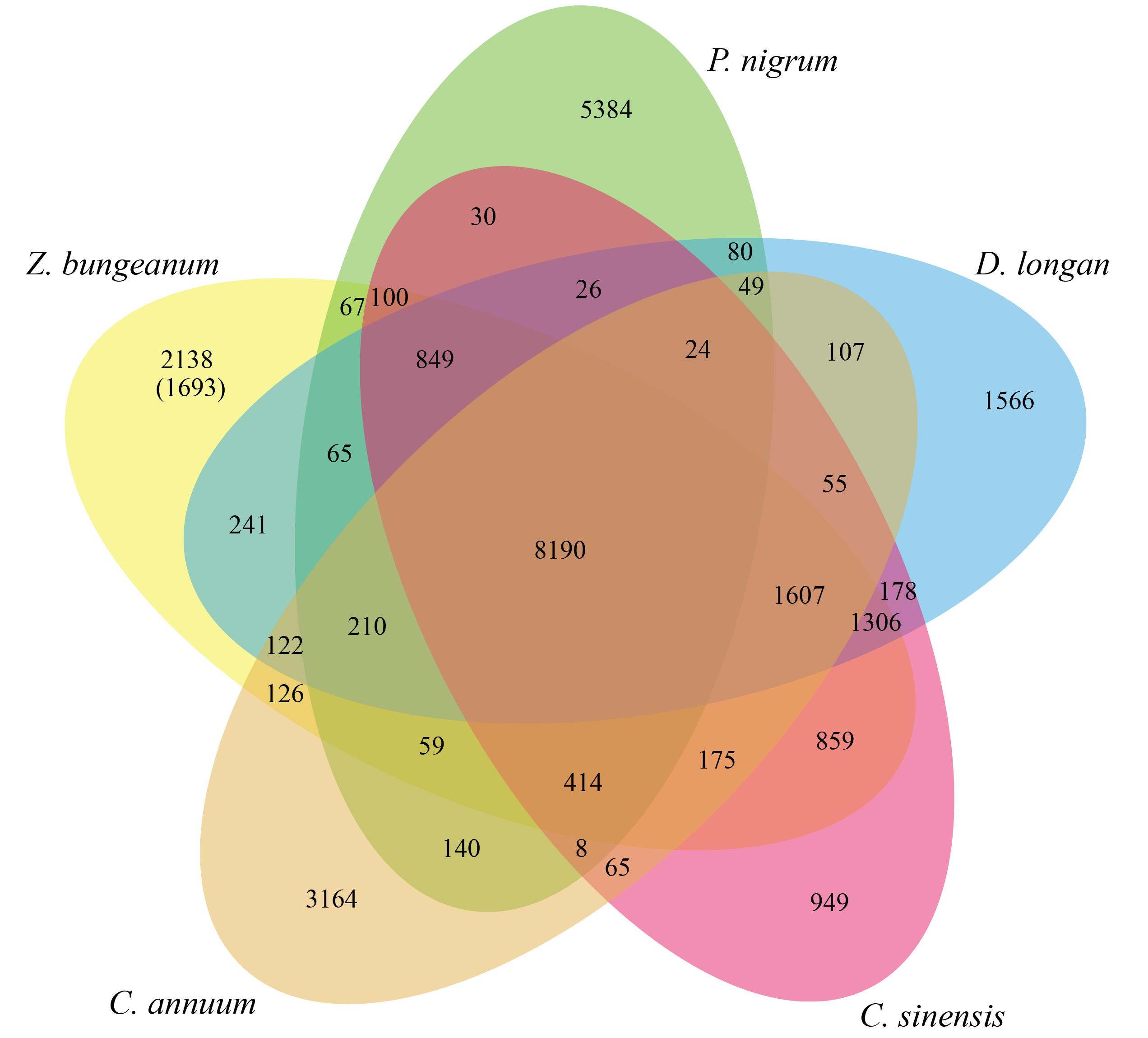


Figure S7 Sharing of gene families by *Z. bungeanum*, *C. annuum*, *P. nigrum* and two other Sapindales plants. The number in parenthesis indicates Chinese pepper-specific gene families among all 17 plants considered.


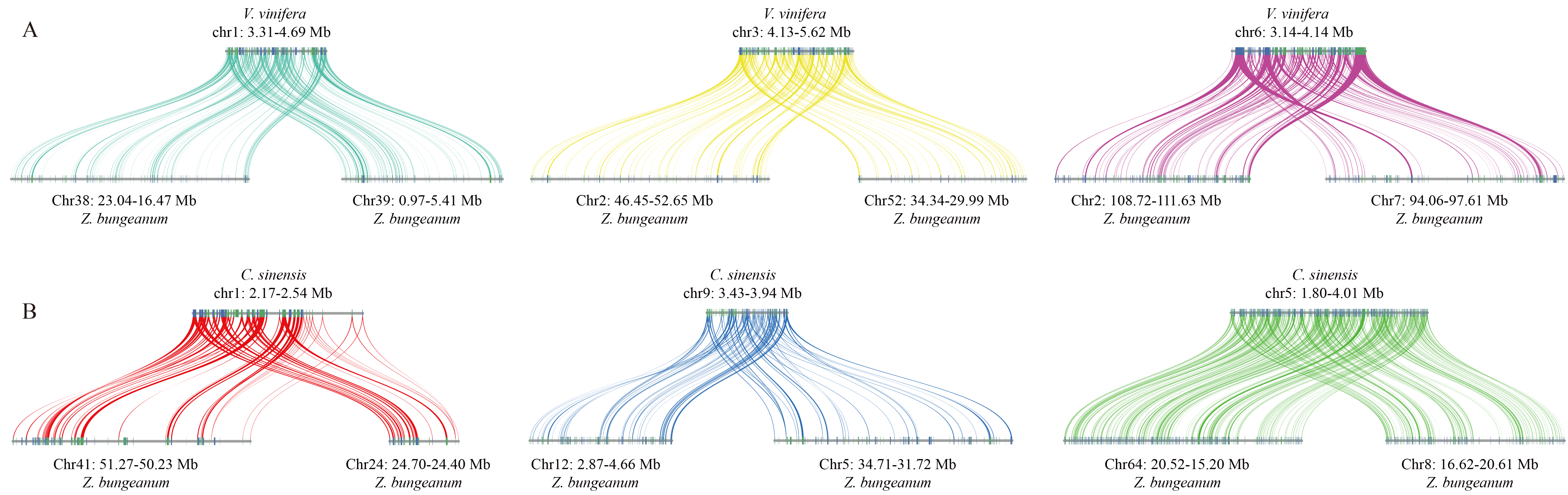


**Figure S8** Example microsynteny analysis indicated that one lineage-specific WGD occurred in *Z. bungeanum*. Rectangles represent predicted gene models, with green and blue colored lines showing relative gene orientations. Colored ribbons connect the matching collinear gene pairs. **(A)** *Z. bungeanum* vs. *V. vinifera* **(B)** *Z. bungeanum* vs. *C. sinensis*.


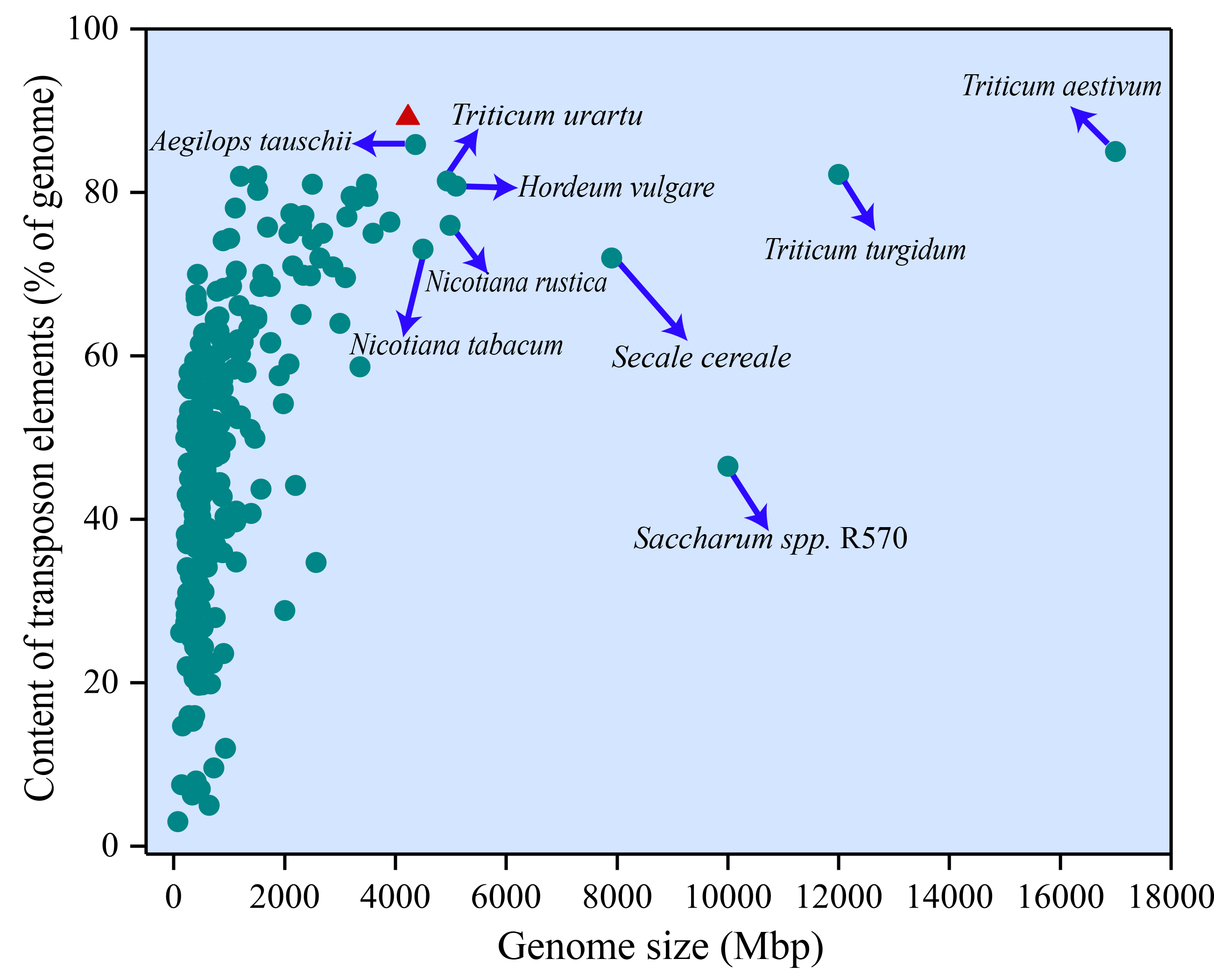


Figure S9 Comparison of the genome size and the content of transposon elements of *Zanthoxylum* with other sequenced plants. The red triangle represents *Z. bungeanum*.


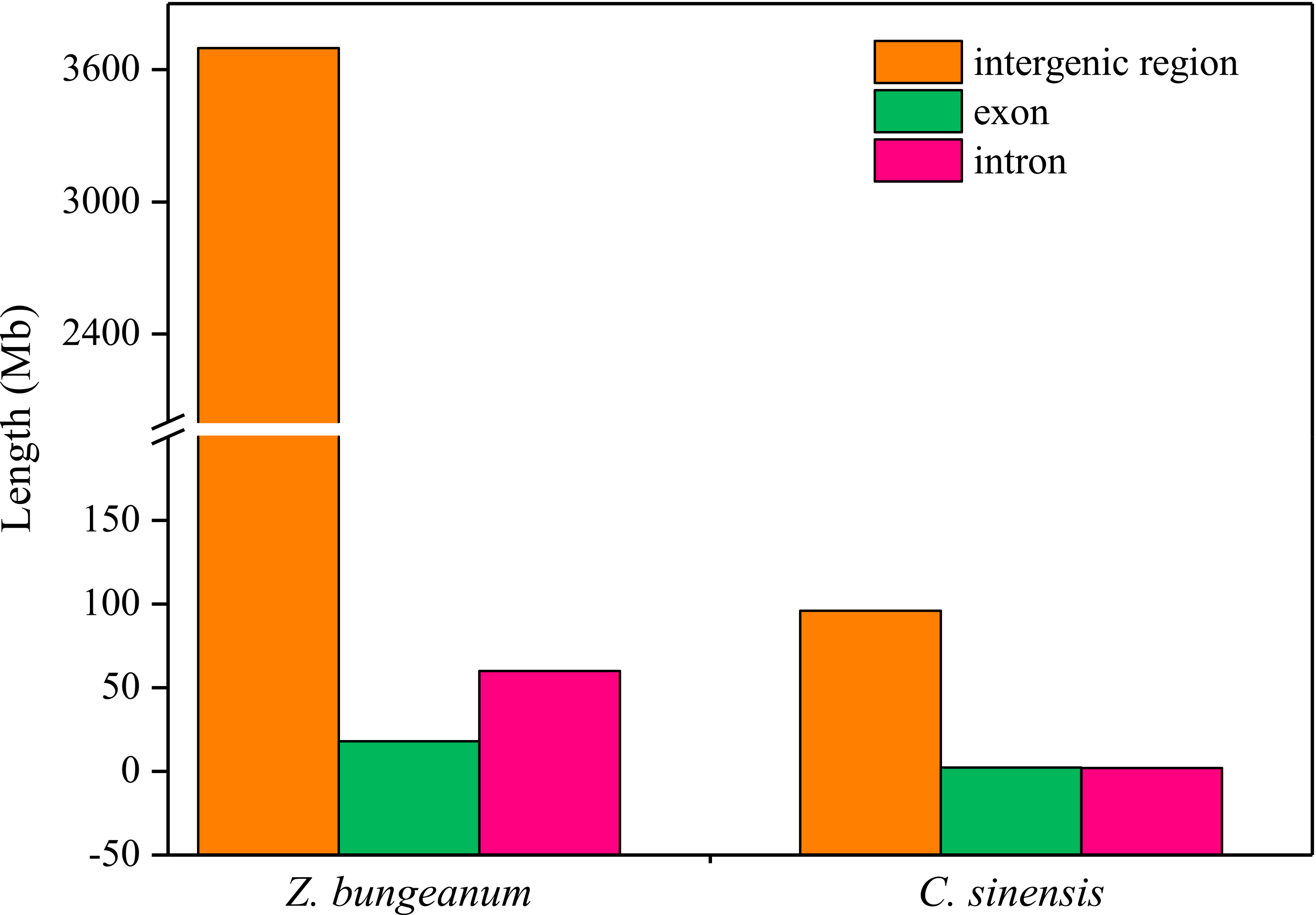


Figure S10 The distribution of repeat elements among the genomes of *Z. bungeanum* and *C. sinensis*.


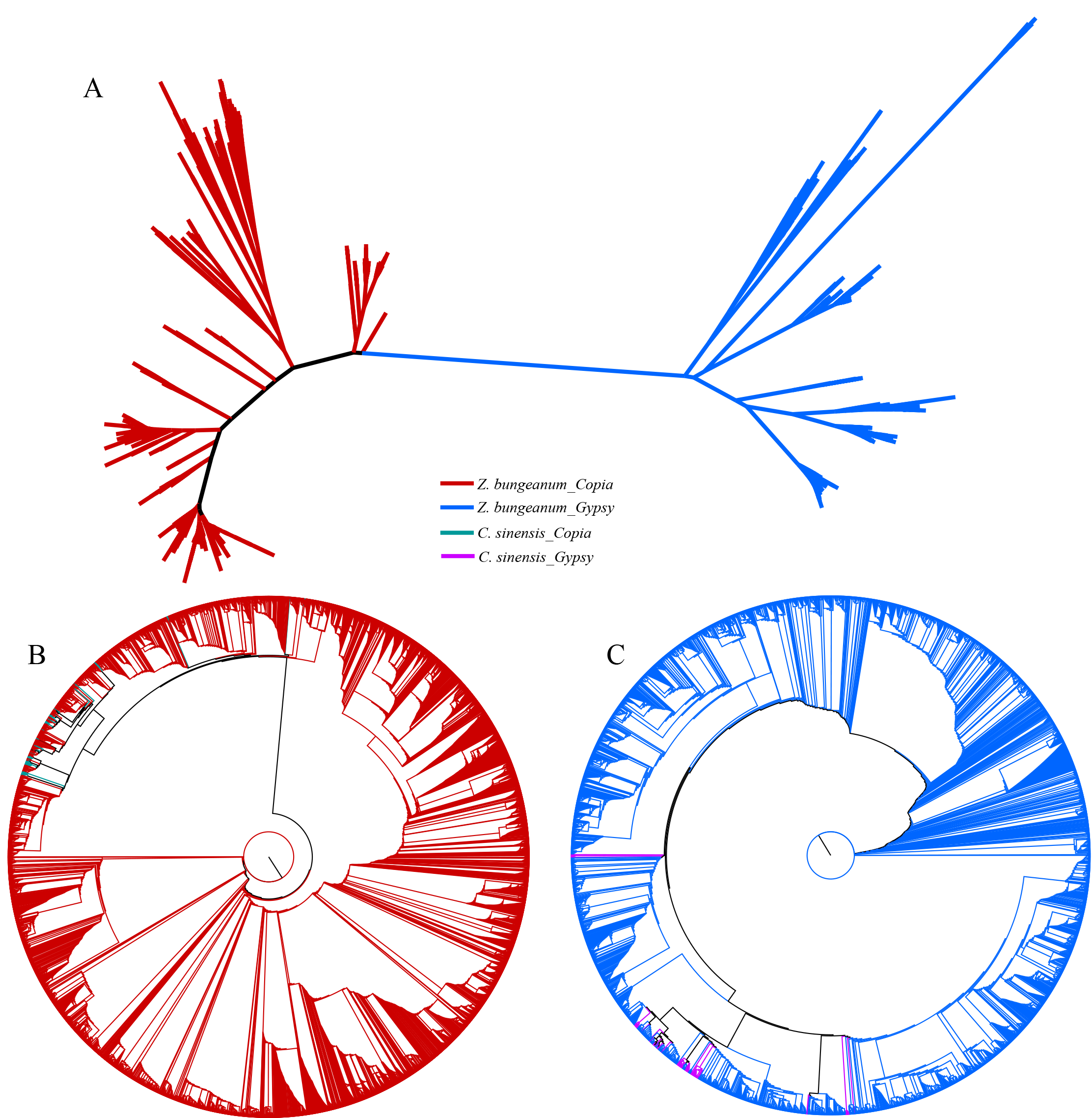


**Figure S11** (A) Phylogenetic trees are shown for *Copia* (red) and *Gypsy* (blue) of *Z. bungeanum*. (B) Phylogenetic trees are shown for *Copia* of *Z. bungeanum* (red) and *C. sinensis* (green). (C) Phylogenetic trees are shown for *Gypsy* of *Z. bungeanum* (blue) and *C. sinensis* (purple).


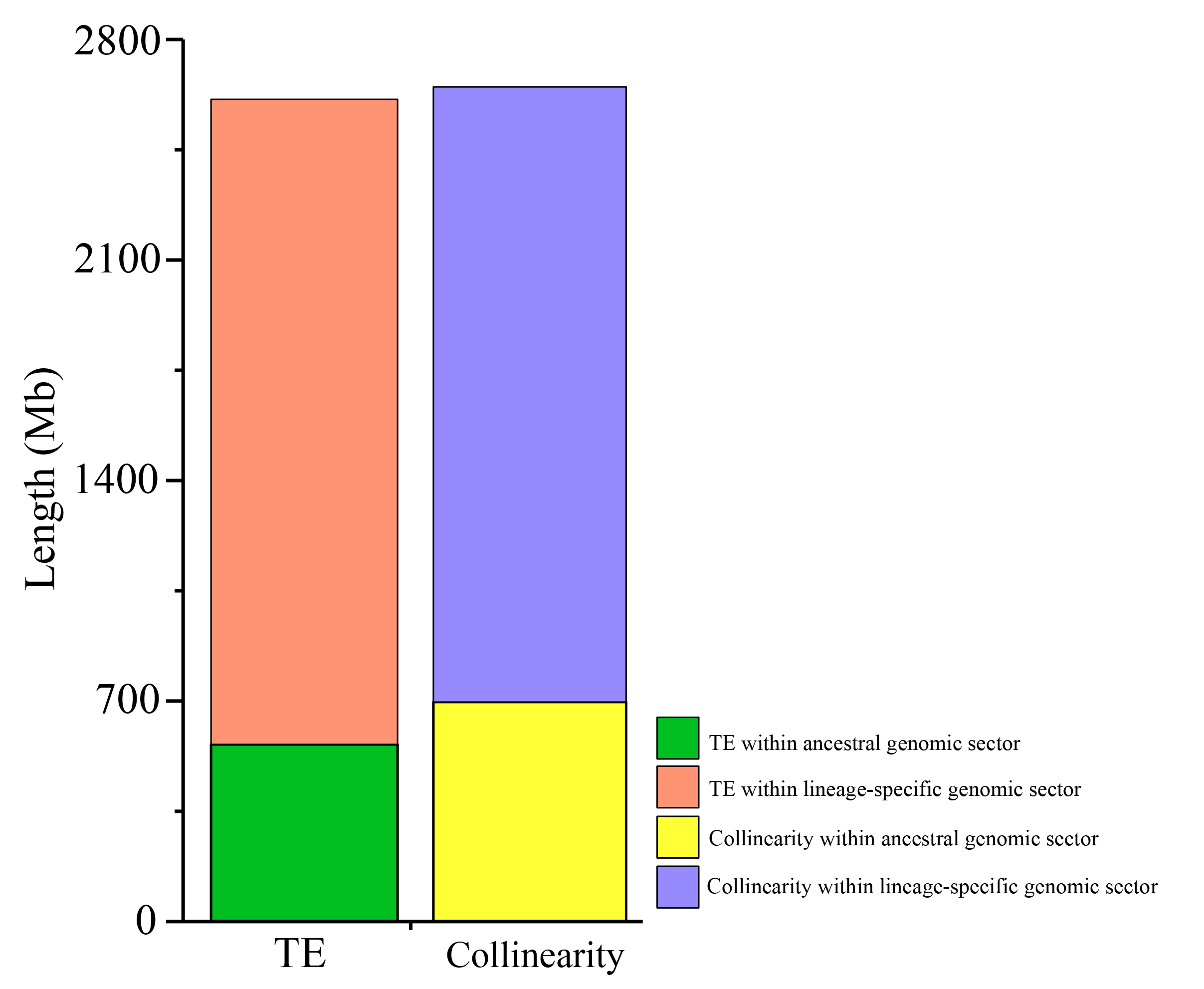


**Figure S12** The percentages of TEs and collinearity within ancestral genomic sector and lineage-specific genomic sector.


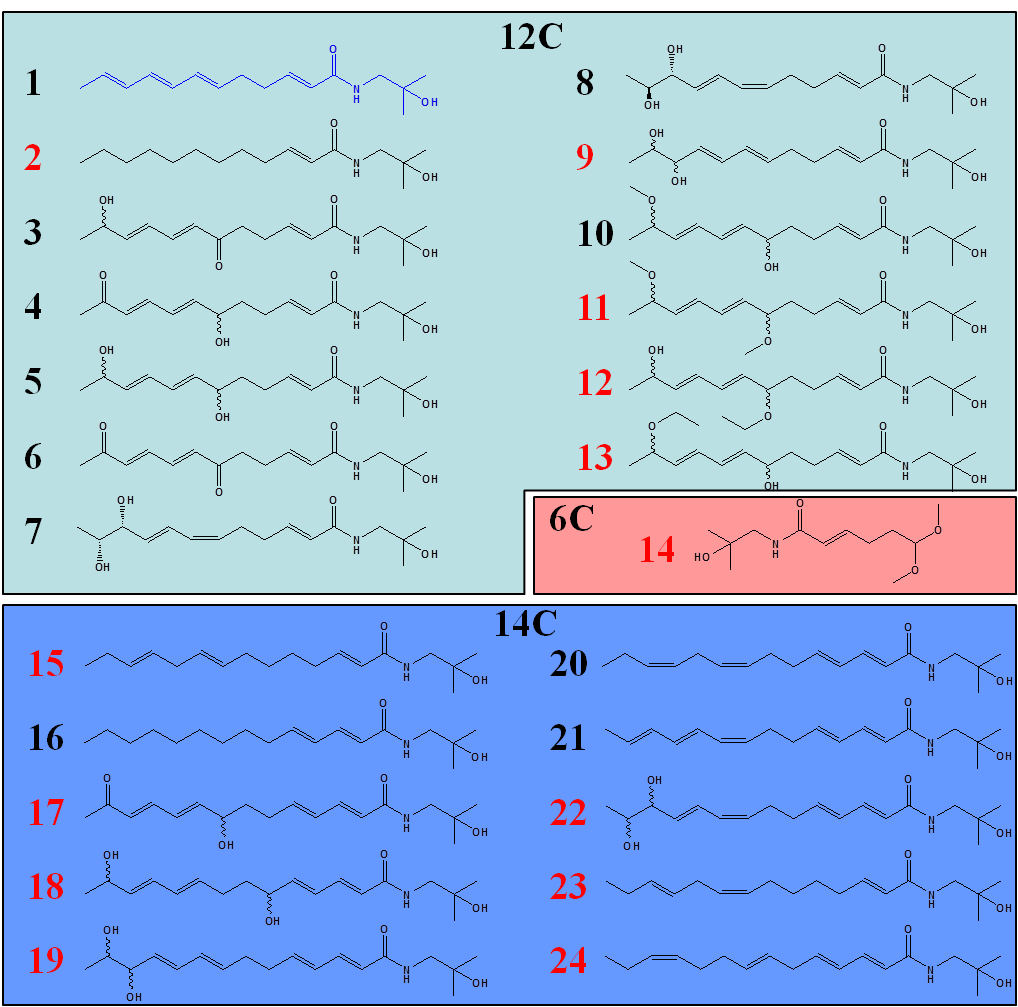


Figure S13 Alkamide from pericarps of *Z. bungeanum.* The red color of labels represented 13 new sanshools first discovered in our study.


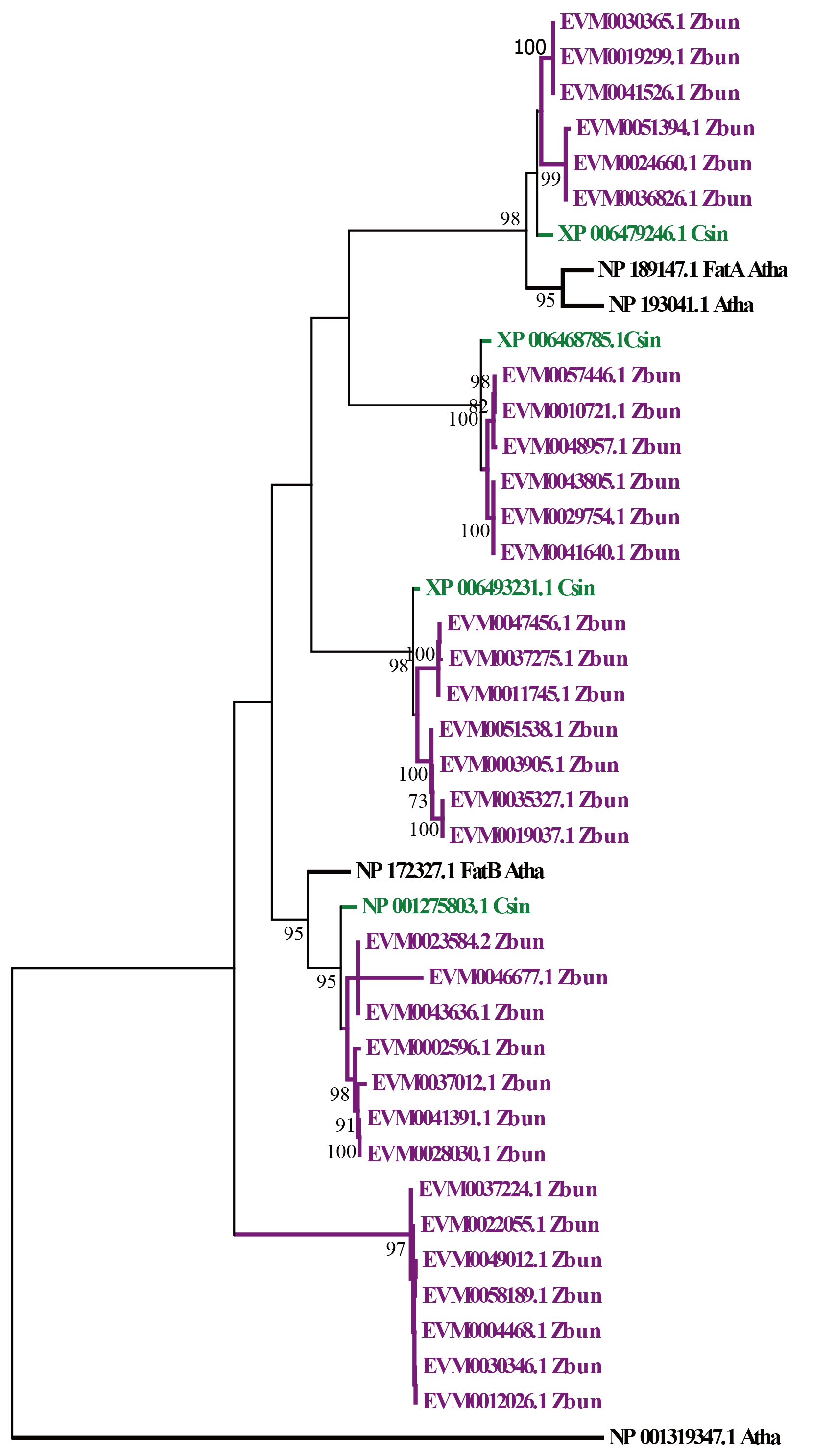


Figure S14 Phylogenetic analysis of the Acyl-ACP thioesterase gene family in *Z. bungeanum* (purple), *C. sinensis* (greeb) and *A. thaliana* (black).


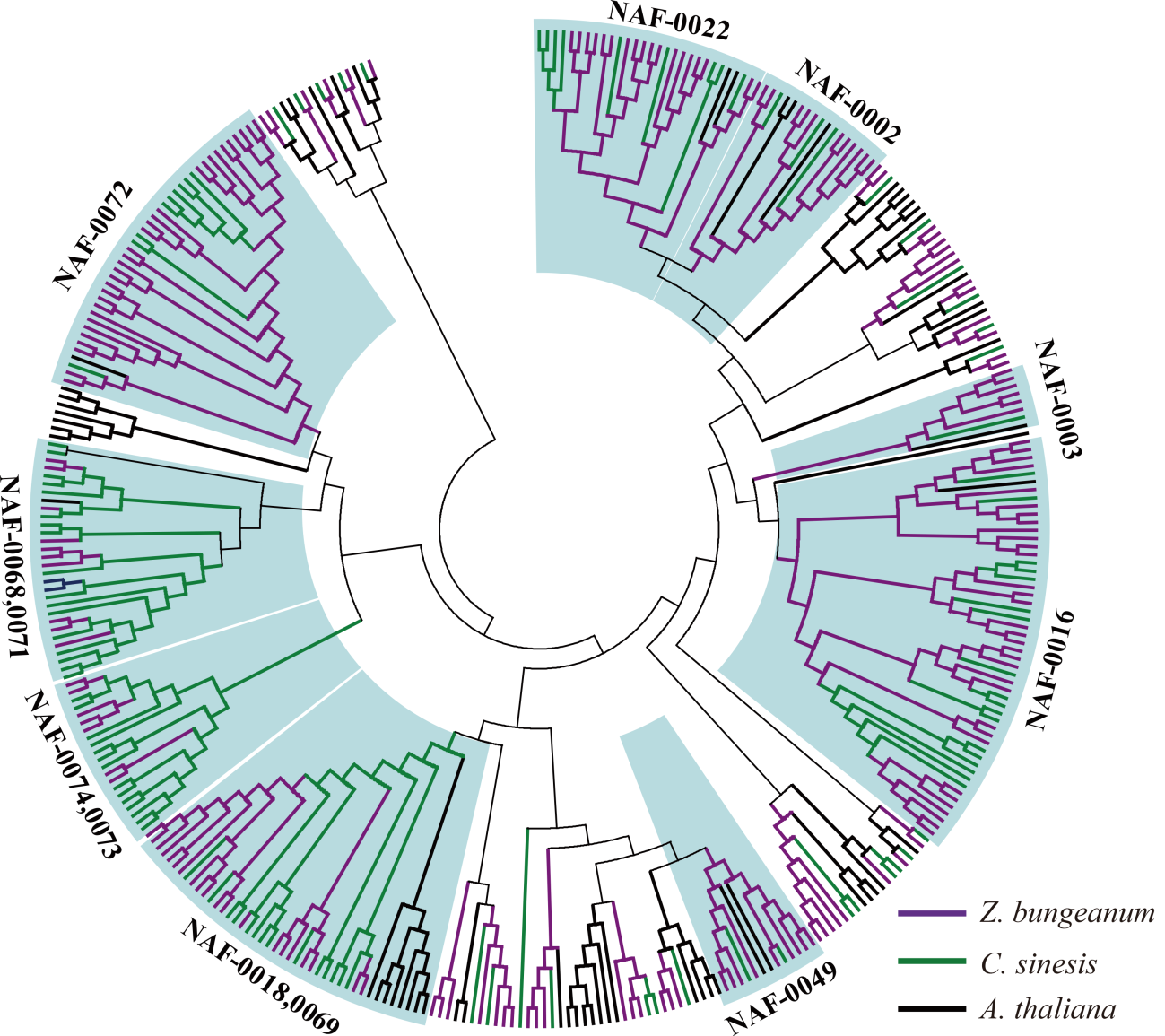


Figure S15 Phylogenetic analysis of the Acetyltransferase (NAF) gene family in *Z. bungeanum* (Purple), *C. sinensis* (Greeb) and *A. thaliana* (Black), which indicated that acetyltransferase is significantly expanded in both *Z. bungeanum* and *C. sinensis*.


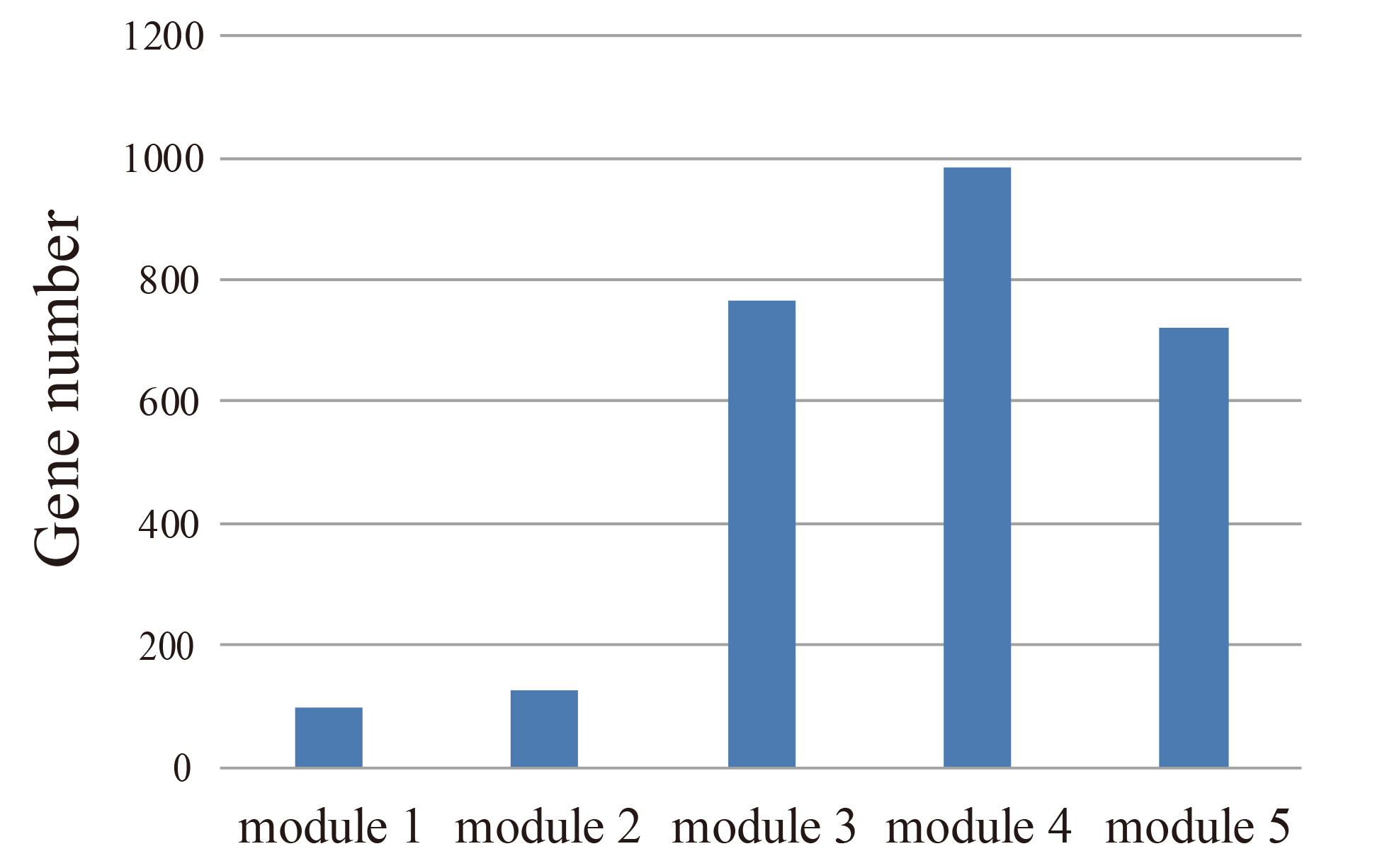


Figure S16 The five modules of 2752 metabolic genes (average FPKM > 5)


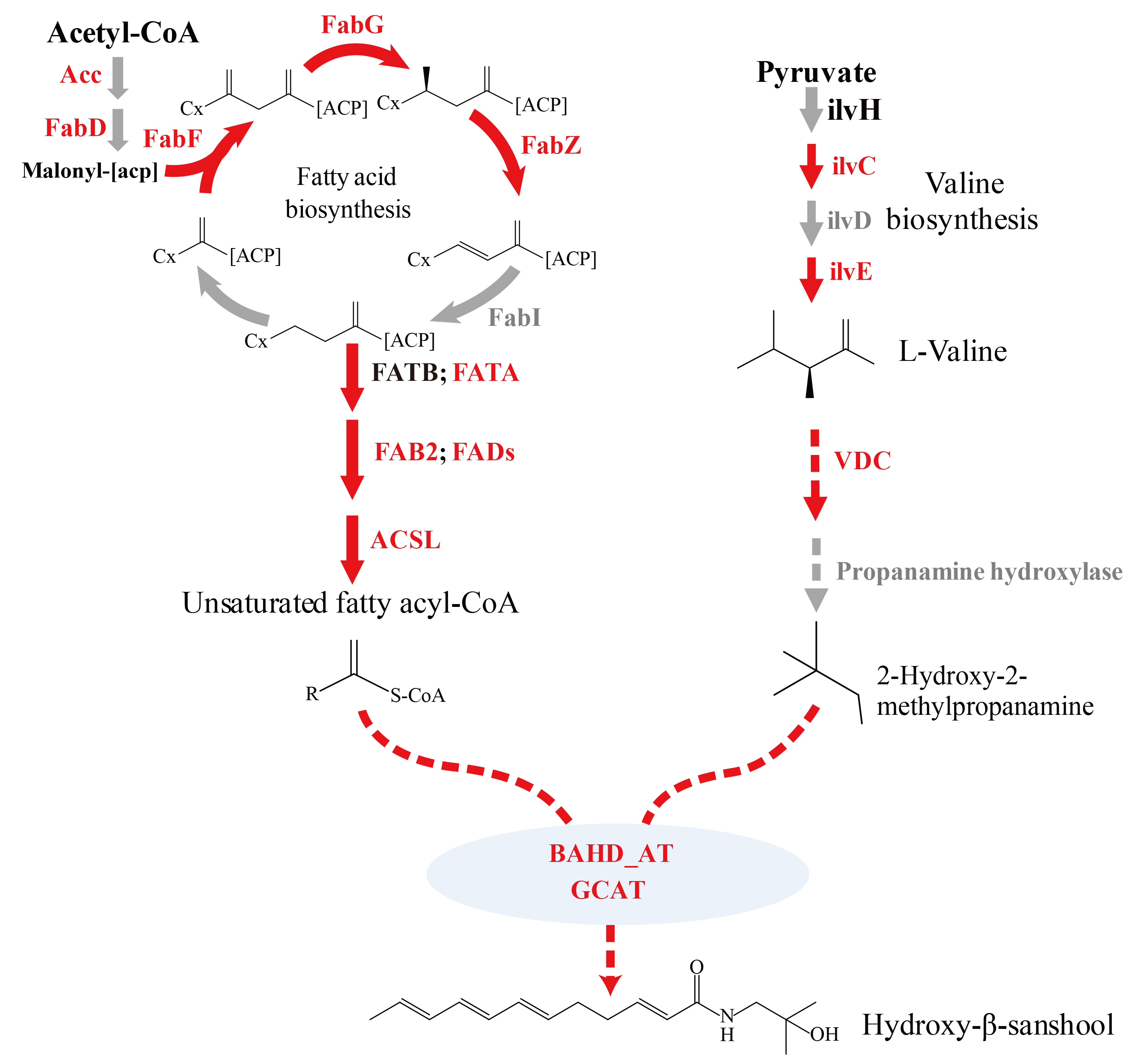


Figure S17 The higher expression level of genes (red) involved in sanshools biosynthesis in *Z. bungeanum*. The gray color represents the genes in *Z. bungeanum* but no ortholog in *C. sinensis*.


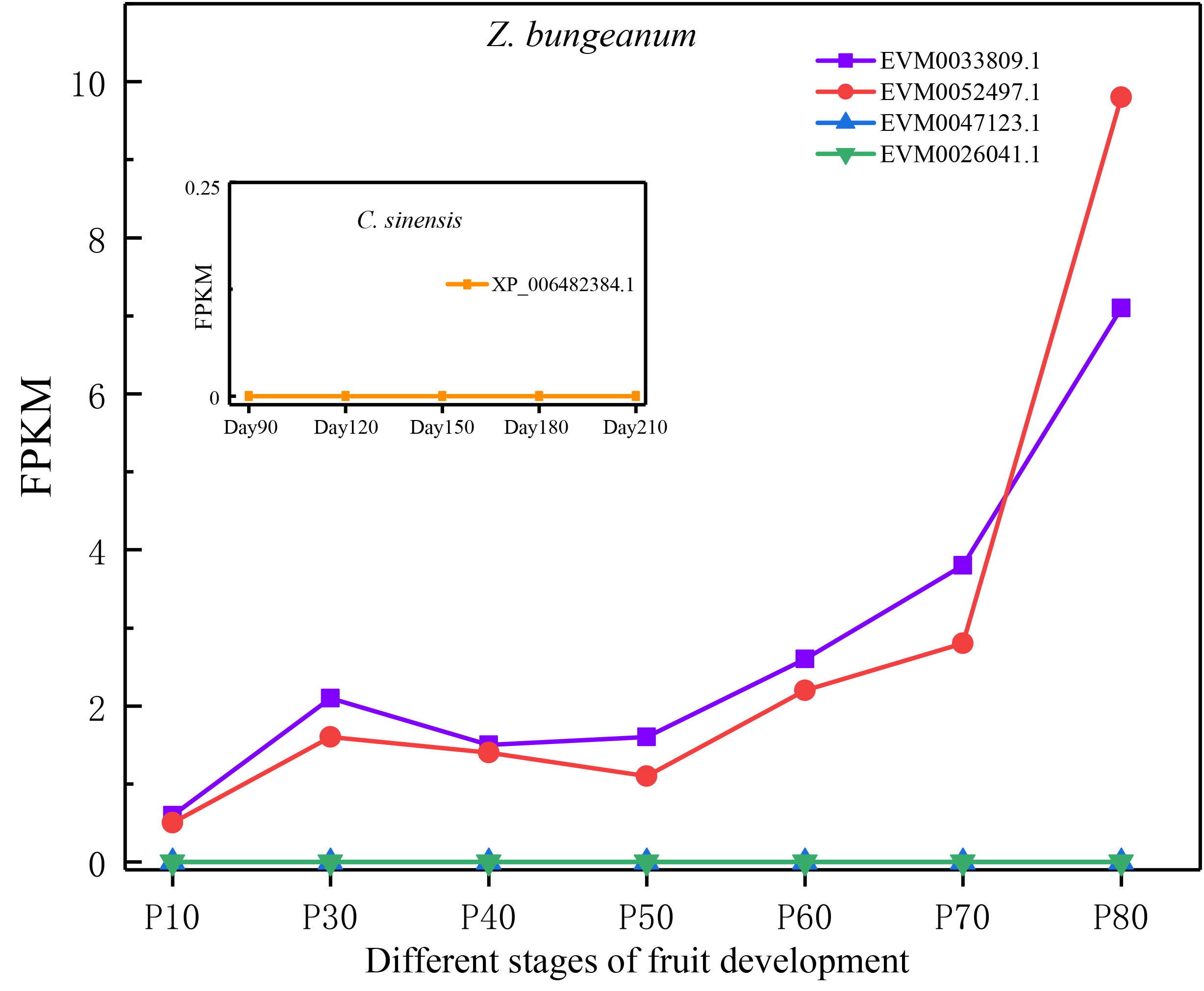


Figure S18 Expression levels of four MYB genes at seven fruit development stages. The gene expression of EVM0033809.1 to Ruby1, XP_006482384.1 in *C. sinensis* was performed at the peels 90, 120, 150, 180 and 210 days after full bloom.


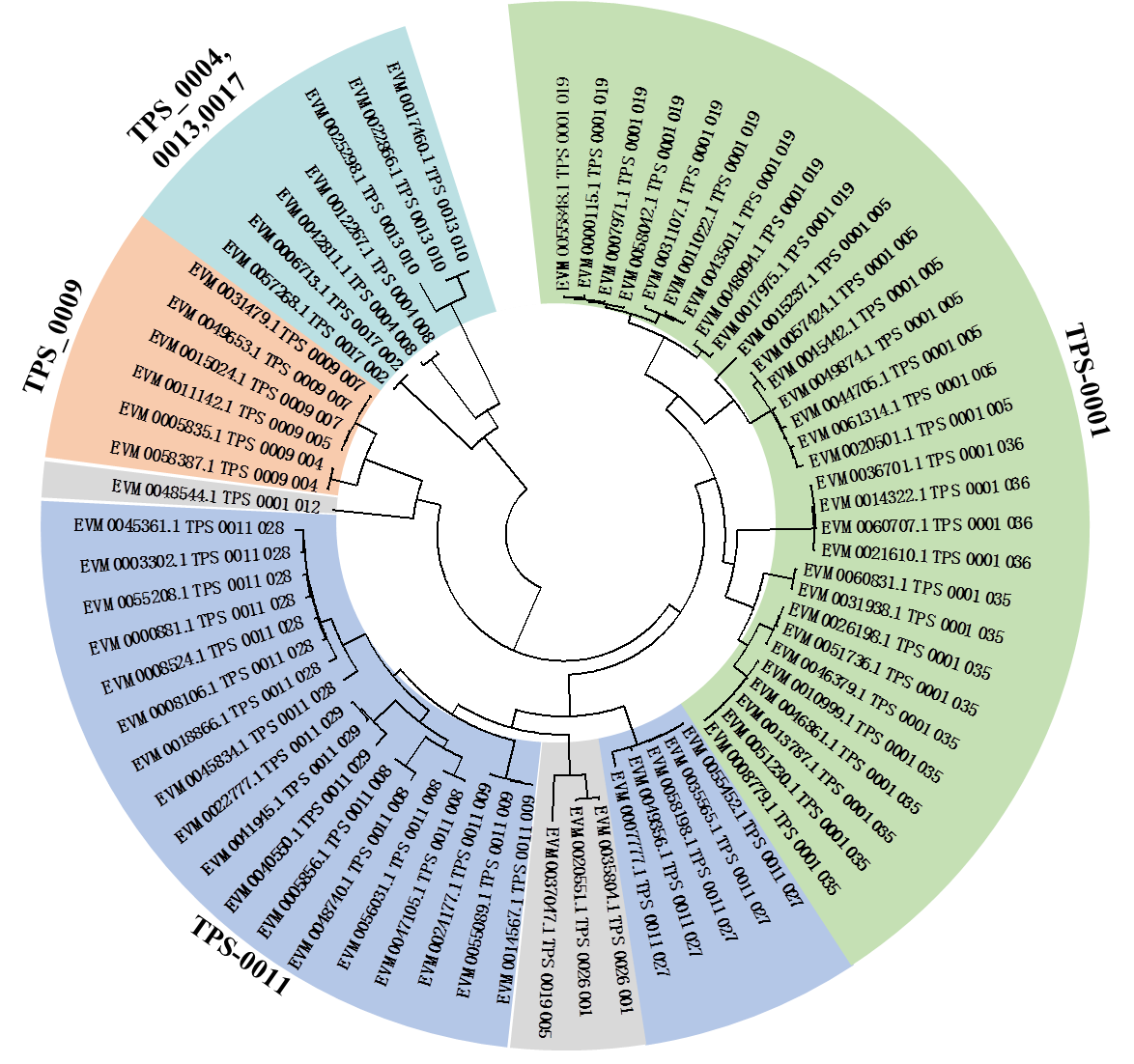


Figure S19 Phylogeneitc tree of 70 TPS genes in *Z. bungeanum*


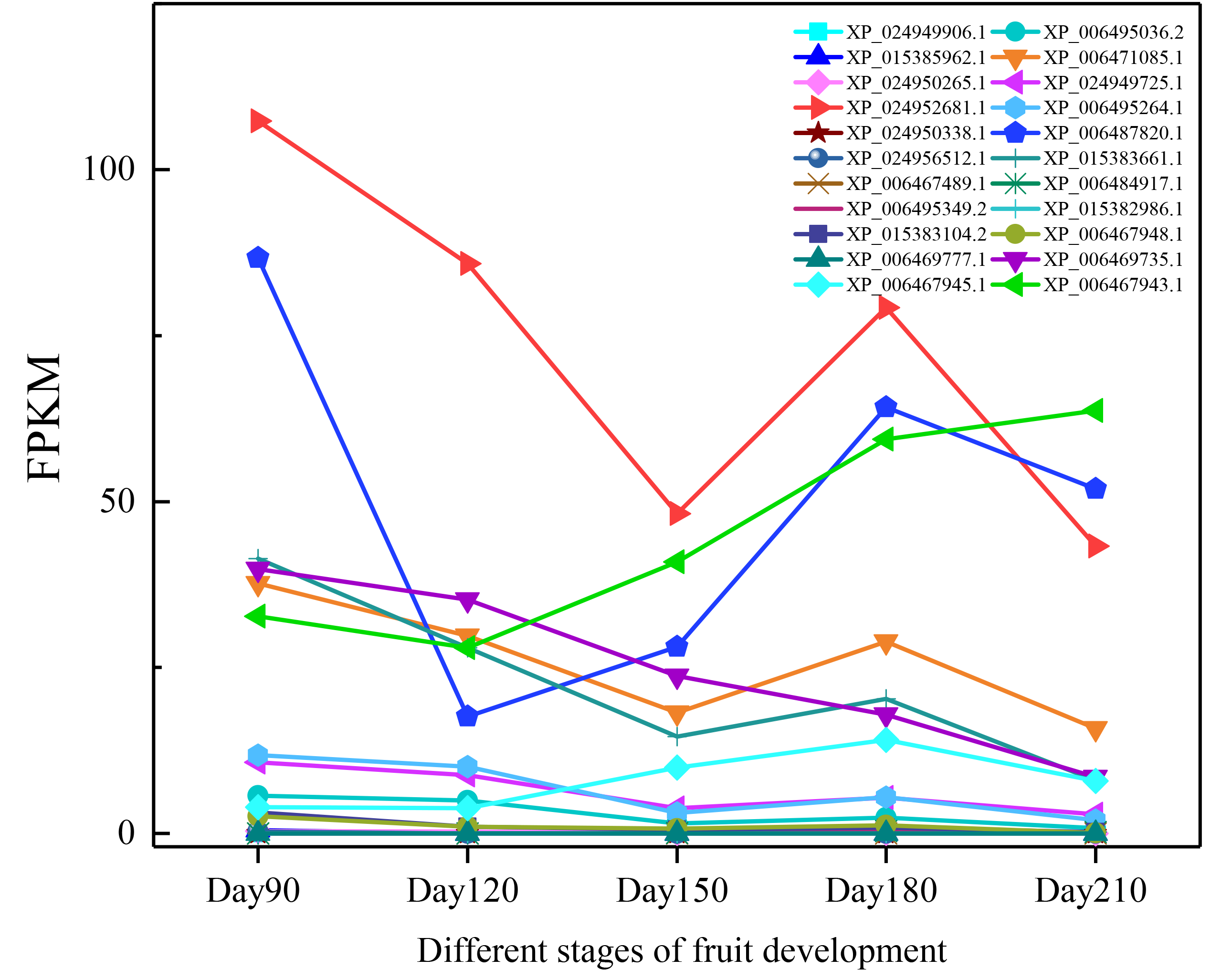


Figure S20 Expression levels of 21 monoterpenoid synthases in *C. sinensis* genome at five fruit developmental periods.
